# Lithium Promotes Longevity through GSK3/NRF2-Dependent Hormesis

**DOI:** 10.1016/j.celrep.2016.03.041

**Published:** 2016-04-07

**Authors:** Jorge Iván Castillo-Quan, Li Li, Kerri J. Kinghorn, Dobril K. Ivanov, Luke S. Tain, Cathy Slack, Fiona Kerr, Tobias Nespital, Janet Thornton, John Hardy, Ivana Bjedov, Linda Partridge

**Affiliations:** 1 Institute of Healthy Ageing and Department of Genetics, Evolution and Environment, University College London, Darwin Building, Gower Street, London WC1E 6BT, UK; 2 Max Planck Institute for Biology of Ageing, Joseph-Stelzmann Strasse 9-b, 50931 Köln, Germany; 3 Department of Molecular Neuroscience, Institute of Neurology, University College London, Queen Square, London WC1N 3BG, UK; 4 European Molecular Biology Laboratory, European Bioinformatics Institute (EMBL-EBI), Wellcome Trust Genome Campus, Hinxton, Cambridge CB10 1SD, UK; 5 UCL Cancer Institute, Paul O’Gorman Building, 72 Huntley Street, London WC1E 6BT, UK

**Keywords:** GSK-3, NRF-2, aging, xenobiotic stress, Keap1, dietary restriction, triglycerides

## Abstract

The quest to extend healthspan via pharmacological means is becoming increasingly urgent, both from a health and economic perspective. Here we show that lithium, a drug approved for human use, promotes longevity and healthspan. We demonstrate that lithium extends lifespan in female and male *Drosophila*, when administered throughout adulthood or only later in life. The life-extending mechanism involves the inhibition of glycogen synthase kinase-3 (GSK-3) and activation of the transcription factor nuclear factor erythroid 2-related factor (NRF-2). Combining genetic loss of the NRF-2 repressor Kelch-like ECH-associated protein 1 (Keap1) with lithium treatment revealed that high levels of NRF-2 activation conferred stress resistance, while low levels additionally promoted longevity. The discovery of GSK-3 as a therapeutic target for aging will likely lead to more effective treatments that can modulate mammalian aging and further improve health in later life.

## Highlights

• Lithium extends *Drosophila* lifespan independent of sex and genetic background
• Lithium reduces triglycerides and confers stress-resistance
• Genetic or pharmacological inhibition of GSK-3 activates NRF-2
• NRF-2 activation is required for the longevity effects of lithium

## Graphical Abstract

**Figure:**
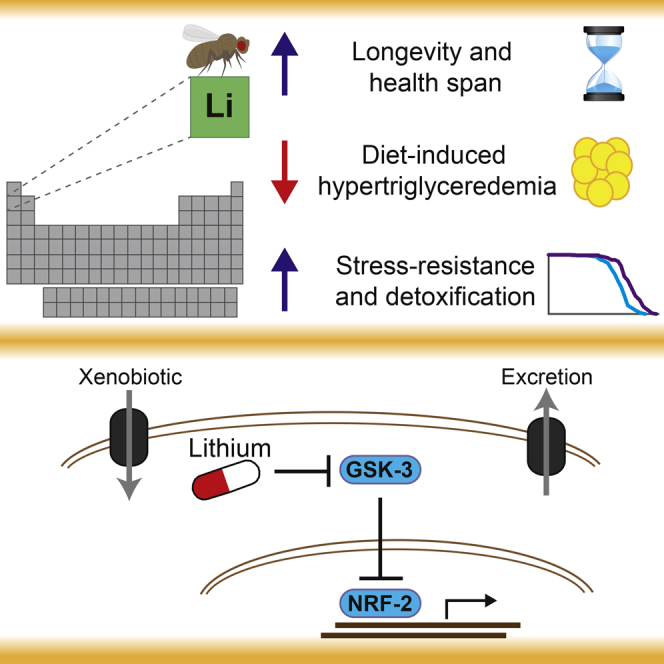

## Teaser

The mood stabilizer lithium has been shown to extend lifespan in organisms ranging from yeast to flies. Castillo-Quan et al. show that lithium promotes longevity through GSK-3 inhibition and subsequent NRF-2 activation, suggesting that GSK3 is a possible drug target that might affect aging.

## Introduction

Lithium is the most commonly prescribed drug for the treatment of bipolar disorder. It also improves disease phenotypes in animal models of many clinical conditions including Alzheimer disease, depression, and stroke (Chiu and Chuang, 2010). The effects of lithium on aging have been documented in yeast and *Caenorhabditis elegans*, with lithium extending lifespan (McColl et al., 2008, Zarse et al., 2011, Tam et al., 2014, Sofola-Adesakin et al., 2014). The effects of lithium on *Drosophila* aging have previously been inconclusive, with demonstration of both positive and negative effects on survival (Matsagas et al., 2009, Zhu et al., 2015). Moreover, lithium concentration in the drinking water of a large Japanese population has been associated with reduced all-cause mortality (Zarse et al., 2011), suggesting that lithium may be a bona fide anti-aging drug. However, the mechanisms by which lithium acts in humans remain poorly understood.

In vitro studies have reported that lithium can protect against several forms of oxidative and xenobiotic stressors (Lai et al., 2006, Schäfer et al., 2004), but in vivo evidence for such protective effects of lithium is lacking. Longevity has been extensively correlated with resistance to stress (Minois, 2000, Rattan, 2008, Calabrese et al., 2011, Epel and Lithgow, 2014). Transcriptomic analysis of interventions known to extend lifespan have identified particular genes likely to be involved in stress resistance (McElwee et al., 2007, Steinbaugh et al., 2012). Upregulation of the transcription factor cap’n’collar C (CncC, an NRF-2 homolog) has been shown not only to confer resistance to toxic compounds, but also to promote longevity in *C*. *elegans* and flies (Tullet et al., 2008, Sykiotis and Bohmann, 2008, Ewald et al., 2015). In flies and mammals, NRF-2/CncC is negatively inhibited through cytosolic sequestration and proteasomal degradation by the canonical Keap1 (Hayes and Dinkova-Kostova, 2014, Pitoniak and Bohmann, 2015). However, a second emerging upstream regulator of NRF-2/CncC is GSK-3, a well-documented target of lithium (Jope, 2003, Hayes and Dinkova-Kostova, 2014, Cuadrado, 2015, Hayes et al., 2015, Blackwell et al., 2015). GSK-3 regulates NRF-2 by phosphorylation and nuclear exclusion, an effect that is evolutionarily conserved from invertebrates to mammals (Salazar et al., 2006, An et al., 2005). Interestingly, GSK-3 inhibition has been shown to phenocopy the effects of lithium for protection against xenobiotic stress in vitro (Lai et al., 2006, Schäfer et al., 2004).

Activation of NRF-2/CncC produces hormetic effects on lifespan, such that at low level NRF-2/CncC activity extends lifespan while higher levels of activation limit it (Mattson, 2008, Maher and Yamamoto, 2010). Interestingly a hormetic signature was recently reported for the survival of a mammalian cell line treated with lithium (Suganthi et al., 2012), suggesting that lithium and GSK-3 inhibition could influence animal lifespan and stress resistance through activation of NRF-2.

Here we show that lithium supplementation in the diet can modulate longevity, stress resistance, and metabolism in *Drosophila* through the inhibition of GSK-3. Correspondingly, genetic downregulation of GSK-3 and lithium treatment are epistatic, suggesting a common molecular pathway. We also show that lithium and the genetic inhibition of GSK-3 promote xenobiotic stress resistance and lifespan extension through the activation of a transcriptional response mediated by CncC/NRF-2. Furthermore, lithium protects against a high-sucrose diet and acts through mechanisms that only partially overlap with those mediating lifespan extension by dietary restriction (DR). These findings demonstrate an alternative genetic and pharmacological target for the promotion of longevity and stress resistance, and emphasize the potential of pharmacological inhibitors of GSK-3 as viable anti-aging treatments.

## Results

### Lithium Extends Healthy Lifespan in *Drosophila*

**Figure 1:**
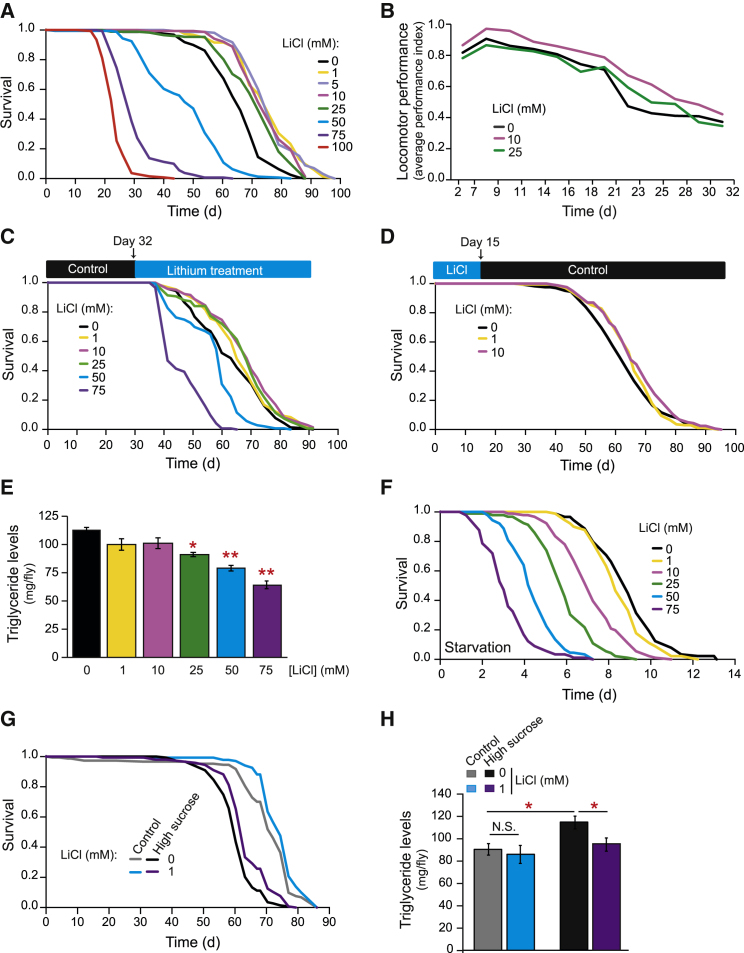
Lithium Regulated Longevity and Metabolism in *Drosophila* (A) Lithium extended lifespan of *w*^*Dah*^*Drosophila* females (n = 160 flies per condition) at concentrations between 1 and 25 mM (+16% and +18% median and maximum lifespan extension; p < 0.001), but resulted in a dose-dependent reduction in lifespan at concentrations between 50 and 100 mM (p < 0.001). (B) Lithium treated female *w*^*1118*^ flies showed a significant improvement and protection against age-related locomotor decline (p < 0.01, two-way ANOVA for 10 mM). (C) Lithium extended lifespan of aged, 32-day-old female *w*^*Dah*^ flies at concentrations from 1 to 25 mM (30 days later than in Figure 1A): 1 mM extended median lifespan by 5% (4 days) and maximum lifespan by 13% (8 days; p < 0.05); 10 and 25 mM lithium increased median lifespan by 9% (6 days); 10 mM increased maximum lifespan by 4.5% (3.5 days); wherease 25 mM lengthened it by 8% or 6 days (p < 0.01); and 50 and 75 mM significantly shortened lifespan (p < 0.01). n = 150 flies per condition. (D) Brief treatment with lithium for 15 days early in adulthood extended lifespan of female *w*^*Dah*^ flies (p < 0.05 for 1 mM and p < 0.01 for 10 mM; n = 150 flies per condition). (E) Lithium induced a dose-dependent reduction in triglyceride levels. Bars represent means of six replicas of five flies per condition ± SEM. ^∗^p < 0.01, ^∗∗^p < 0.001. (F) Female *w*^*Dah*^ flies pre-treated with lithium for 15 days were subsequently sensitive to starvation in a dose-dependent manner (n = 90 flies per condition). (G) Lithium treatment significantly extended the lifespan of *w*^*1118*^ female flies exposed to a four times higher sucrose concentration (2g/L; p < 0.001; n = 120 flies per condition). (H) The increase of triglycerides observed on a high-sucrose diet was completely blocked after 15 days of treatment with 1 mM lithium. Bars represent means of six replicas of five *w*^*1118*^ female flies per condition ± SEM. ^∗^p < 0.01.

To assess the role of lithium in *Drosophila* aging, we treated adult female flies with lithium chloride (LiCl) by supplementation in their food. Lithium treatment in the range of 1 to 25 mM resulted in lifespan extension, whereas higher doses (50–100 mM) shortened lifespan (Figure 1A). These effects of lithium treatment on lifespan extension were also observed in an independent genetic background (Figure S1A) and in males (Figure S1B). Thus, lithium treatment extended *Drosophila* lifespan independently of genetic background and sex.

To ensure that the increased lifespan observed with lithium supplementation was dependent on the addition of lithium itself, we treated flies with equivalent molar concentrations of sodium chloride (NaCl) and found no lifespan extension (Figures S1C and S1D). Thus, the pro-longevity effect of LiCl is specific to lithium and not its chloride counterion.

Interestingly, we observed that, unlike with many other genetic and pharmacological interventions (e.g., DR, insulin/IGF downregulation, rapamycin, or trametinib treatment), lithium did not reduce fecundity at life-extending doses or compromise feeding behavior (Figures S1E and S1F). Moreover, it delayed locomotor decline at two concentrations that extend lifespan (Figure 1B). Thus, lithium promotes healthspan in adult *Drosophila* with limited side effects.

### Lithium Extends Lifespan in Mid-life or with Short-Term Treatment in Young Flies

To limit the side effects of long-term use, a drug that improves lifespan and healthspan will ideally do so with late-onset administration (Castillo-Quan et al., 2015, Longo et al., 2015). We therefore assessed the effect of commencing lithium treatment at older ages. Flies were switched onto food containing a range of lithium concentrations (1–75 mM) at 32 days of age (Figure 1C). Lower doses (1–25 mM) of lithium extended lifespan, whereas higher doses (50 and 75 mM) significantly reduced lifespan, similar to the dose-dependent effects we observed in younger flies.

We also tested whether transient lithium treatment early in life could increase lifespan. We therefore exposed young flies to 1 or 10 mM lithium for 15 days and then switched them to control food for the remainder of their lifespans. Early treatment with these doses of lithium extended lifespan (Figure 1D). Lithium treatment early in life, and for a transient period, can therefore increase survival later in life.

### Lithium Alters Lipid Metabolism and Promotes Survival under a High-Sugar Diet

Genetic and environmental interventions that extend lifespan often induce abnormalities in carbohydrate and lipid metabolism (Barzilai et al., 2012, Wang et al., 2014, Lamming et al., 2013). We therefore examined the effects of lithium on whole body trehalose, glycogen, and triglyceride levels. Following 15 days of lithium treatment, and over a wide range of lithium concentrations, we were unable to detect a significant change in the levels of either trehalose or glycogen (Figures S1G and S1H). However, we observed a dose-dependent reduction in whole body triglycerides, the main lipid storage in flies (Ballard et al., 2008, Skorupa et al., 2008) (Figures 1E and S1I). In keeping with the lowered triglyceride levels (Ballard et al., 2008, Ulgherait et al., 2014), lithium treatment reduced survival under starvation conditions in a dose-dependent manner (Figures 1F and S1J). Moreover, lithium also extended lifespan under dietary conditions that promote triglyceride accumulation (Skorupa et al., 2008). Flies fed a high-sucrose diet were short lived and lithium was able to partially rescue this defect (Figure 1G) while completely blocking the increase in triglycerides observed with a sucrose-rich diet (Figure 1H). Therefore, lithium can extend lifespan under obesogenic dietary conditions.

### Lithium and DR Extend Lifespan via Partially Overlapping Mechanisms

**Figure 2:**
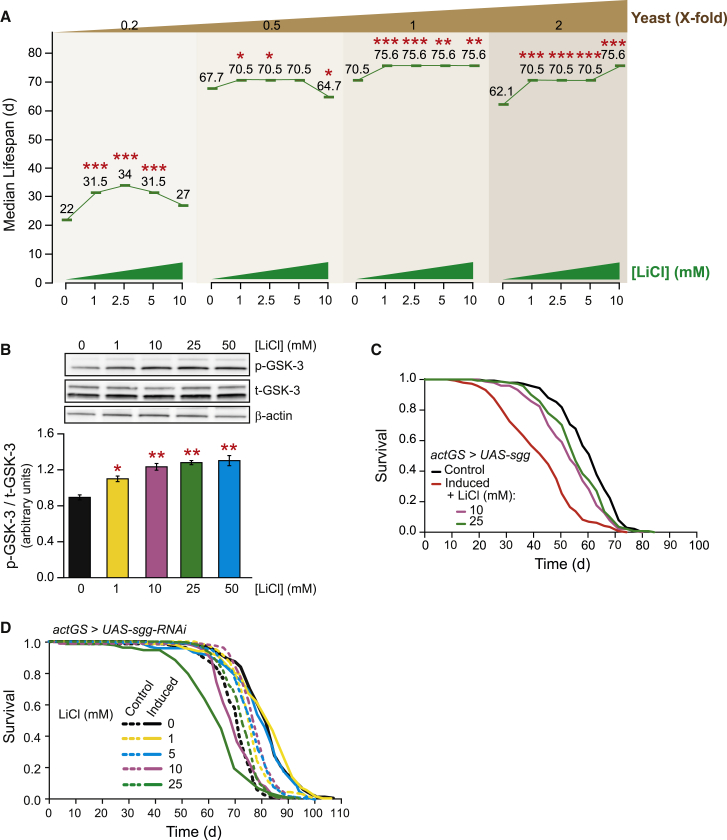
Lithium Extended Lifespan beyond Dietary Restriction by Inhibiting Sgg/GSK-3 (A) Median lifespans at different lithium concentrations (0, 1, 2.5, 5, or 10 mM) are plotted for four different yeast concentrations (0.2×, 0.5×, 1.0×, and 2.0× yeast): 1–5 mM lithium extended lifespan under all dietary conditions tested. Although 10 mM lithium prolonged life at 1.0× and 2.0×, it showed no effect at 0.2× and significantly shortened lifespan at 0.5× yeast. ^∗^p < 0.05, ^∗∗^p < 0.01, ^∗∗∗^p < 0.001, from 0 lithium; n = 160 flies per condition. Complete survival curves are shown in Figures S2A–S2D. (B) Lithium treatment for 15 days significantly increased the inhibitory phosphorylation of Sgg/GSK-3 in a dose-dependent manner. Bars represent means of triplicates of ten flies per biological repeat ± SEM, ^∗^p < 0.05, ^∗∗^p < 0.01. (C) Ubiquitous overexpression of wild-type *sgg* significantly shortened lifespan (p < 0.001) and this was partially rescued by lithium treatment at two concentrations (10 and 25 mM; p < 0.001). See Figure S2E for the interaction of *sgg(S9A)* and lithium treatment on lifespan. (D) Ubiquitous RNAi-mediated downregulation of *sgg* extended lifespan (p < 0.001) and no further extension occurred when the flies were treated with 1 or 5 mM lithium (p > 0.05), whereas 10 mM lithium treatment restored the lifespan to control levels (p > 0.05), and 25 mM was significantly toxic (p > 0.05). See Figure S2F for lifespan extension obtained with an independent RNAi line.

We next investigated whether lithium treatment was acting as a DR mimetic. DR is a well-established anti-aging intervention that extends healthy lifespan in diverse species (de Cabo et al., 2014, Fontana and Partridge, 2015), and some pharmacological and genetic interventions that extend lifespan have features of DR mimetics (Ingram and Roth, 2015, de Cabo et al., 2014). To determine whether lithium and DR extend lifespan by similar mechanisms, we assessed whether lithium could extend lifespan beyond the maximum achievable by DR. To maximize lifespan under DR, we varied the yeast concentration in the food while maintaining a constant concentration of sucrose (Bass et al., 2007), resulting in a typical tent-shaped response, with peak lifespan at food containing a 1.0 yeast concentration (Figures 2A and S2A–S2D). If lithium treatment and DR share overlapping pathways, then lithium would not be able to further extend lifespan already maximized by DR (Gems et al., 2002, Castillo-Quan et al., 2015). All lithium doses tested significantly extended median lifespan in both the yeast condition that maximized lifespan (1.0 yeast; Figures 2A and S2C) and under full feeding (2.0 yeast; Figures 2A and S2D), with greatest extension of median lifespan with 10 mM lithium under full feeding. However, under reduced yeast concentrations that shorten lifespan (0.2 and 0.5 yeast), 10 mM lithium either significantly reduced lifespan (Figure S2B) or did not confer a significant lifespan benefit (Figure S2A). Cox proportional hazards analysis showed a significant interaction between lithium and yeast concentrations for lifespan (interaction term p < 0.0001). The extension of lifespan from lithium increased with the level of yeast in the fly diet, suggesting partially overlapping mechanisms to those of DR.

### Lithium Extends Lifespan through Inhibition of GSK-3

A well-known target of lithium is GSK-3 (Phiel and Klein, 2001, Jope, 2003, Eldar-Finkelman and Martinez, 2011). We therefore evaluated the phosphorylation status of the fly ortholog of GSK-3, Shaggy (Sgg), in response to lithium treatment. Lithium addition to the fly medium resulted in a dose-dependent increase in the inhibitory phosphorylation (Serine 9 or S9) of Sgg (Figure 2B). To evaluate the role of Sgg in lithium-mediated lifespan extension, we directly manipulated its activity in adult flies. Ubiquitous overexpression of wild-type or constitutively active Sgg (SggS9A) significantly reduced lifespan by ∼30% and 50%, respectively (Figures 2C and S2E). This reduction in lifespan was almost completely reversed by lithium treatment. Furthermore, RNAi-mediated reduction in *sgg* expression using two independent dsRNA-expressing transgenes significantly increased lifespan (Figures 2D and S2F). Importantly, lithium was unable to further increase the lifespan of these *sgg* RNAi knockdown mutants flies (Figure 2D). Taken together, these findings suggest that Sgg/GSK-3 inhibition and lithium treatment increase lifespan by acting on the same downstream targets.

### Lithium Activates the Cap’n’Collar C/NRF-2 Transcription Factor

**Figure 3:**
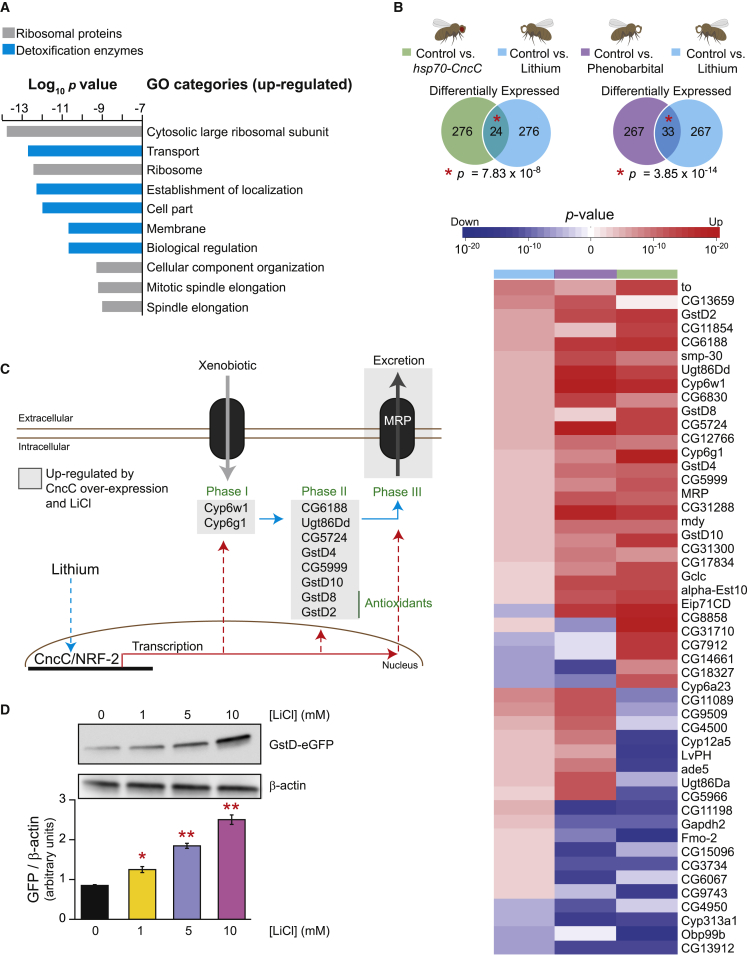
Lithium Activated a Transcriptional Response Similar to that of CncC/NRF-2 (A) Ten most significantly upregulated GO categories induced by lithium treatment of *w*^*1118*^ female flies. See Figure S3A for downregulated GO categories. (B) Lithium treatment of *w*^*1118*^ females flies induced a transcriptional response that significantly overlapped with that induced by *cncC* overexpression (p = 7.83 × 10^−8^) or phenobarbital treatment (p = 3.85 × 10^−14^) (Misra et al., 2011). Heatmap showing the 57 genes most significantly changed by lithium or phenobarbital treatment and overexpression of *cncC*. (C) Genes upregulated by lithium treatment mapped to the three phases of the xenobiotic detoxification pathway in flies. (D) Lithium treatment of *w*^*Dah*^ female flies upregulated Gst-D protein levels. Bars represent means of triplicates of ten flies per condition ± SEM. ^∗^p < 0.05, ^∗∗^p < 0.01.

To identify downstream mediators of lifespan extension by lithium and of GSK-3 inhibition, we analyzed the genome-wide transcript profiles of lithium-treated flies using microarrays. Genes encoding ribosomal proteins were among the most upregulated (Figure 3A) and downregulated (Figure S3A) gene ontology (GO) categories in lithium-treated flies. This transcriptional response could underlie the translational repression following lithium treatment that has been previously observed in fission yeast and *Drosophila* heads (Sofola-Adesakin et al., 2014). In addition, five GO terms for genes encoding enzymes in the detoxification pathway were also in the ten most upregulated categories (Figure 3A).

The responses to xenobiotics and oxidative stress in *Drosophila* are regulated by the transcription factors dFOXO, CncC, and DHR96 (Salih and Brunet, 2008, Sykiotis and Bohmann, 2010, Tullet, 2015, Hoffmann and Partridge, 2015, Blackwell et al., 2015). We therefore assessed whether the transcriptional responses to activation of these transcription factors overlapped with that of lithium treatment. The transcriptomic response to lithium did not overlap with that of dFOXO-dependent or -independent transcriptional regulation downstream of IIS (Figures S3B and S3C) (Alic et al., 2011). Furthermore, although we detected a significant overlap in the transcriptional signatures of lithium and DHR96 (King-Jones et al., 2006), they did not share the same directionality (Figure S4). However, we found a significant overlap (Figure 3B) between the genes that were upregulated by lithium and *cncC* overexpression (Misra et al., 2011), but not between genes downregulated by both treatments (Figure S5A), suggesting that lithium might activate a CncC transcriptional response downstream of GSK-3. The barbiturate phenobarbital activates CncC and induces a similar transcriptional response to that of *cncC* overexpression (Misra et al., 2011). We therefore analyzed the overlap between the transcriptional profiles induced by lithium and phenobarbital treatment, and again found a significant overlap (Figure 3B) between upregulated, but not downregulated, genes (Figure S5B). The genes upregulated in common between lithium treatment, phenobarbital treatment and *cncC* overexpression (Figures 3B and S5C) encoded enzymes that participate in all three phases of xenobiotic metabolism (Figure 3C). To further confirm the activation of CncC by lithium, we used a previously generated CncC reporter that responds to both chemical and genetic inducers of CncC (Sykiotis and Bohmann, 2008). Flies carrying the GstD-eGFP CncC reporter showed a dose-dependent increase in GFP expression with increasing concentrations of lithium (Figure 3D). Taken together, our results suggest that lithium activates CncC to upregulate the expression of genes in the detoxification pathway.

### Lithium Induces Lifespan-Extension, Hormesis, and Protection against Xenobiotics via CncC-Dependent Mechanisms

**Figure 4:**
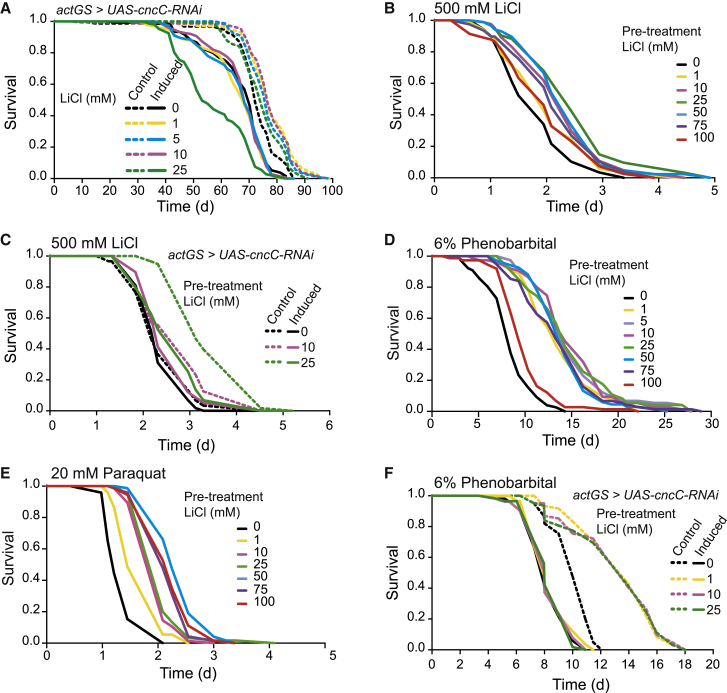
Lithium-Induced Xenobiotic Resistance and Longevity Were Mediated by CncC (A) Ubiquitous knockdown of *cncC* blocked lifespan extension by lithium. (B) Pre-treatment with increasing concentrations of lithium protected against a subsequent toxic dose of lithium (500 mM; p < 0.01 for doses from 10 to 100 mM; p < 0.05 for 1 mM). (C) Ubiquitous downregulation of *cncC* blocked the protective effect of 10 mM lithium pre-treatment against a subsequent toxic dose and partially blocked the protective effect of 25 mM lithium pre-treatment. (D) 1 to 100 mM lithium pre-treatment protected against a 6% phenobarbital (p < 0.001 for all doses). (E) Lithium pre-treatment (for 15 days) protected against the herbicide paraquat in a dose-dependent manner (p < 0.001 for all doses, with maximal protection at 50 mM). (F) RNAi-mediated downregulation of *cncC* completely blocked the protective effect of lithium against phenobarbital.

We next assessed whether CncC activity is required for the pro-longevity effects of lithium. Ubiquitous, RNAi-mediated knockdown of *cncC* expression blocked the lifespan extension of 1 to 10 mM lithium, but was detrimental to survival in flies treated with 25 mM lithium, the highest dose that extends lifespan under basal conditions, albeit to a lesser extent (Figure 4A). Thus, lithium treatment requires CncC activity to confer its longevity benefits.

Because CncC/NRF-2 can induce hormesis (Mattson, 2008, Maher and Yamamoto, 2010), we assessed whether lithium can also do this. To test for a hormetic effect of lithium at low doses, we pre-treated flies with a range of concentrations of lithium and then challenged them with a toxic dose of 500 mM. Most pre-treatment doses of lithium induced subsequent resistance to the toxic dose (Figure 4B). To assess whether the hormetic response of lithium was mediated by CncC, we knocked down expression of *cncC* using RNAi, and treated the flies with 1–25 mM lithium. Reduction in *cncC* expression completely blocked the hormetic response induced by 10 mM lithium pre-treatment, and significantly reduced the effect of 25 mM lithium (Figure 4C).

We next assessed the ability of lithium pre-treatment to protect against other xenobiotics. Flies pre-treated with increasing concentrations of lithium ranging from 1 to 100 mM were significantly resistant to a toxic concentration of phenobarbital, with lithium doses between 1 and 75 mM almost doubling survival (Figure 4D). Lower doses of lithium also protected against a toxic dose of the anti-malarial drug, chloroquine (Figure S5D; 1–10 mM), and the pesticide paraquat (Figure 4E). Thus, low to intermediate concentrations of lithium protect against xenobiotic toxicity. To determine the role of CncC activity in lithium-mediated protection against phenobarbital, we used RNAi to knock down expression of *cncC*, which sensitized the flies to phenobarbital and completely abrogated the protection against phenobarbital afforded by lithium supplementation (Figure 4F). Thus, CncC is at least partly responsible for the hormetic effect induced by low-level treatment with lithium.

**Figure 5:**
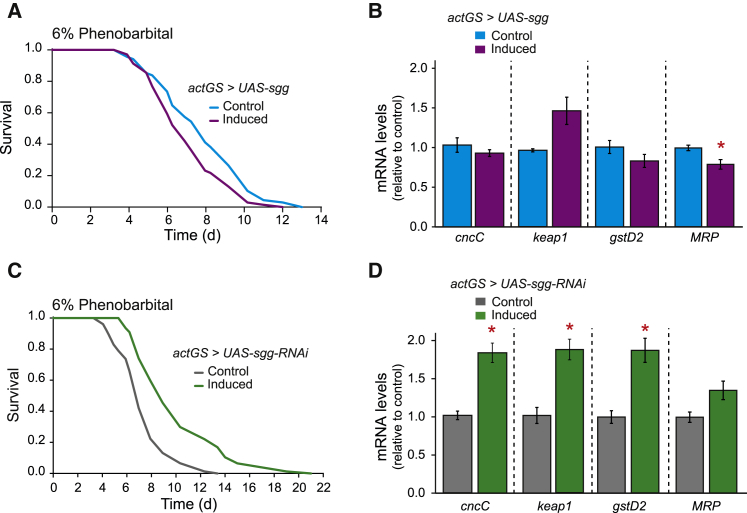
Reduced Activity of GSK-3 Increased Resistance to Xenobiotics (A) Ubiquitous overexpression of wild-type *sgg* significantly (p < 0.05) reduced survival under xenobiotic stress with phenobarbital. n = 75 flies per condition. (B) Overexpression of wild-type *sgg* significantly reduced multidrug-resistance like protein 1 (*MRP*) mRNA levels (p < 0.05, paired t test), whereas non-significant trends were detected for glutathione S transferase D2 (*gstD2*) and *cncC* mRNA levels (p > 0.05). A non-significant increase of *keap1* mRNA levels was observed. (C) RNAi-mediated knockdown of *sgg* protected against phenobarbital stress (p < 0.001). n = 75 flies per condition. (D) Knockdown of *sgg* increased mRNA levels of *cncC*, *keap1* and *gstD2* (p < 0.05), while a non-significant increase was observed for *MRP* mRNA levels.

To confirm that Sgg, upstream of CncC, is also necessary for the resistance to xenobiotic stress (Blackwell et al., 2015, Cuadrado, 2015, Hayes et al., 2015), we assessed the effect of ubiquitous overexpression of wild-type *sgg* or the constitutively active Sgg(S9A) on xenobiotic resistance. Both significantly sensitized flies to phenobarbital (Figures 5A and S5E). We confirmed that *sgg* or *sgg(S9A)* overexpression regulated CncC by showing significantly lower levels of *MRP* and *keap1* (Figures 5B and S5F), both CncC target genes. Correspondingly, RNAi-mediated knockdown of *sgg* resulted in resistance to phenobarbital (Figure 5C), and paraquat (Figure S5G). An increase of mRNA levels of *cncC*, *keap1*, and *gstD2* confirmed that CncC was active in *sgg* knockdown flies (Figure 5D). Thus, increased Sgg activity sensitizes against xenobiotic stressors, whereas its inhibition protects against them.

### Lifespan and Stress Resistance Depend on the Degree of Activation of CncC by Keap1 and Lithium Treatment

**Figure 6:**
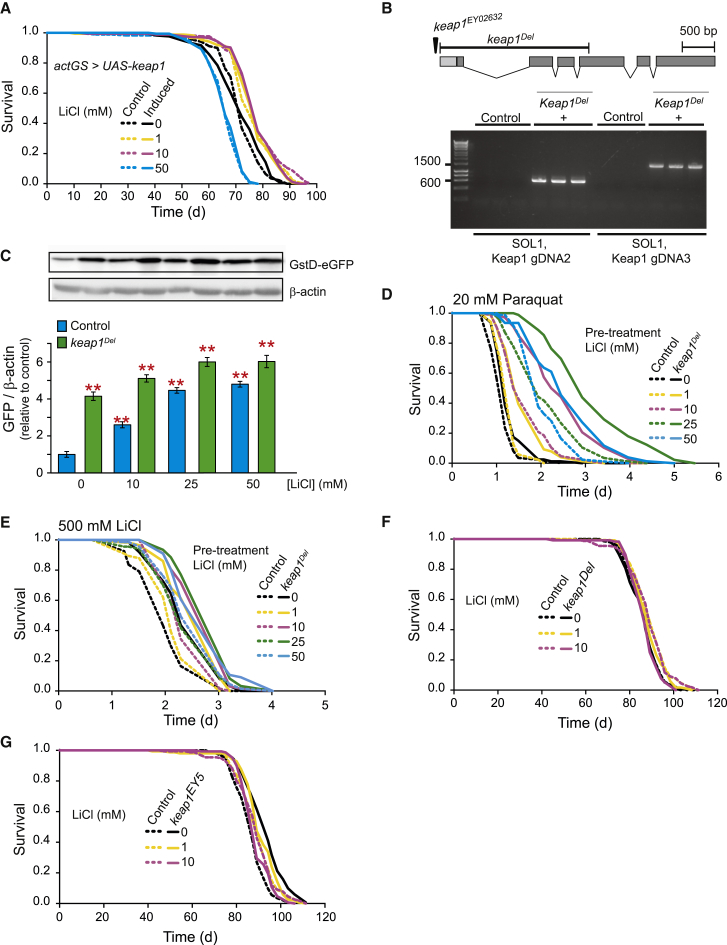
Higher Activation Levels of CncC Promote Xenobiotic Resistance but Not Lifespan (A) Overexpression of *keap1* did not prevent the lifespan-modulatory effects of lithium treatment. n = 150 flies per condition. (B) Schematic of the *keap1* gene showing the portion deleted in the *keap1*^*Del*^ mutant (top) and agarose gel showing start and end of P-element disrupting *keap1* coding sequence in the *keap1*^*Del*^ mutant (bottom). (C) Combination of heterozygous deletion of *keap1* and lithium treatment showed a greater activation of CncC than on their own. Bars represent means of four replicas of five flies per repeat ± SEM. ^∗∗^p < 0.01. (D) Deletion of *keap1* in flies treated with lithium showed greater protection against paraquat than either treatment on its own, with maximal effects observed at 25 mM (p < 0.001). (E) The *keap1* deletion protected against toxic concentrations of lithium (500 mM), and this protection was augmented with lithium pre-treatment (p < 0.01). (F) Deletion of *keap1* did not extend lifespan: 1 mM lithium (p < 0.05), but not 10 mM (p > 0.05), treatment of *keap1* flies resulted in a small but significant extension. n = 150 flies per condition. (G) *keap1*^*EY5*^ mutant flies showed significant lifespan extension (p < 0.001), that was dose-dependently abolished (p > 0.05) by lithium, likely as a result of over-activation of CncC. n = 150 flies per condition.

In addition to activating CncC by repressing Sgg/GSK-3, lithium could potentially increase CncC activity by inhibiting its canonical repressor Keap1 (Cuadrado, 2015, Pitoniak and Bohmann, 2015). Hence, we analyzed the interaction between lithium treatment and Keap1. Overexpression of Keap1, which inhibits CncC activity in vivo (Sykiotis and Bohmann, 2008), was unable to prevent the lifespan-extending properties of lithium (Figure 6A), suggesting that the longevity effect of lithium treatment is independent of Keap1. Next, we analyzed the interaction of loss of Keap1 and lithium treatment. We generated a deletion of the *keap1* coding sequence by P-element-mediated male recombination using a previously described P-element insertion line (Sykiotis and Bohmann, 2008) (Figure 6B). The *keap1* deletion (*keap1*^*Del*^) was homozygous lethal, but activated CncC 4-fold in the heterozygous state, as measured by the CncC reporter (Figure 6C). Lithium treatment of the *keap1*^*Del*^ flies further activated CncC (Figure 6C). We next tested whether this effect on CncC activation protected against paraquat and lithium toxicity. *keap1*^*Del*^ flies were significantly resistant to both paraquat and lithium (Figures 6D and 6E), and pre-treatment with lithium further protected them. We confirmed these findings using a previously described heterozygous loss-of-function mutation in the *keap1* gene (*keap1*^*EY5*^) (Sykiotis and Bohmann, 2008) (Figures S6A and S6B). Thus, the combination of loss of *keap1* and lithium treatment further protected against paraquat and lithium-induced toxicity, suggesting that stronger CncC activation results in greater protection against these xenobiotics.

We subsequently evaluated the interaction between loss of *keap1* and lithium treatment for longevity. Survival analysis showed that the lifespan of *keap1*^*Del*^ mutant flies was indistinguishable from controls, but that addition of 1 mM lithium marginally, yet significantly, extended lifespan (Figure 6F). Increasing the dose of lithium to 10 mM restored longevity to control levels. The *keap1*^*EY5*^ mutant flies showed a significant lifespan extension (Figure 6G). However, supplementation of either 1 or 10 mM lithium to the *keap1*^*EY5*^ mutant shortened lifespan in a dose-dependent manner. These results suggest that the level of activation of CncC that maximizes extension of lifespan is considerably lower than that which maximizes protection against toxic doses of lithium and paraquat.

### Lithium Does Not Induce or Require Autophagy to Promote Longevity

**Figure 7:**
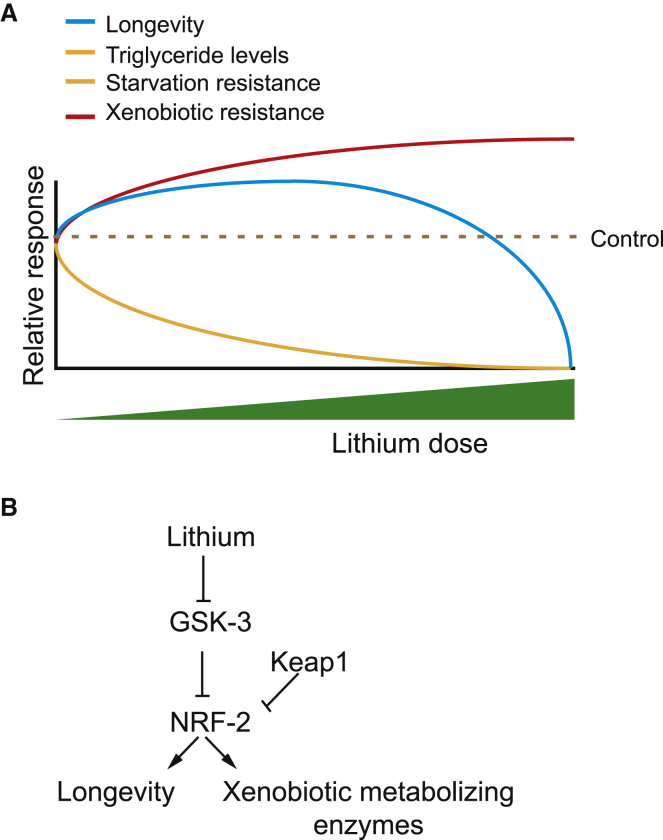
Lithium Regulates Longevity, Metabolism, and Stress Resistance by Inhibiting GSK-3 and Activating NRF-2 (A) Summary of findings with lithium for longevity, stress resistance, starvation, and triglyceride levels. (B) Proposed model showing the mechanism by which lithium, Sgg/GSK-3, and CncC/NRF-2 act in the same pathway to modulate longevity and xenobiotic resistance.

Activation of autophagy has been proposed as a mechanism for the beneficial effects of lithium (Sarkar et al., 2005). We therefore analyzed the induction of autophagy by LC3-I/LC3-II (Atg8 in *Drosophila*) levels without detecting statistically significant changes. Indeed, there was a tendency for lower LC3-I that did not reach statistical significance (Figure S7A). Moreover, lithium treatment was able to extend the lifespan of flies with autophagy defects due to heterozygous loss of *atg1* (Figure S7B) (Lee et al., 2007). Thus, taken together our results do not immediately support a role for autophagy in the pro-longevity effects of lithium treatment, and strengthen our conclusion that they are mediated through the inhibition of GSK-3 and the subsequent activation of CncC/NRF-2 (Figure 7). However, it remains possible that induction of autophagy occurs in *atg1*-deficient flies, or that lithium induces autophagy in a tissue-specific manner.

## Discussion

### Lithium Acts as a Pro-longevity Drug

Drug repurposing is the most promising approach for developing pharmacological agents to improve healthy aging. So far, two medically approved drugs, metformin and rapamycin, have been reported to promote longevity and provide health benefits across species from invertebrates to mammals (de Cabo et al., 2014, Madeo et al., 2014, Riera and Dillin, 2015). We and others have shown that lithium can extend lifespan in fission yeast, *C*. *elegans*, and *Drosophila* (McColl et al., 2008, Matsagas et al., 2009, Sofola-Adesakin et al., 2014). We also showed that this effect was common between two different laboratory strains and, unlike other interventions that seem to be more effective in females (Austad and Bartke, 2015), lithium similarly extended lifespan in both sexes.

Lifespan-extending drugs can often act like DR mimetics (Madeo et al., 2014, Ingram and Roth, 2015); hence, it was important to determine whether lithium was acting in a similar manner. While low doses of lithium were able to extend lifespan at all dietary levels tested, median lifespan extension was greatest under full feeding conditions. Our data thus suggest that lithium and DR act via partially overlapping mechanisms and confirms the observation made in *C*. *elegans* that lithium extends lifespan of *eat-2* mutants (McColl et al., 2008), a genetic model of DR in worms. Lithium also extended the lifespan of flies fed a diet enriched with sucrose, possibly by modulating lipid metabolism (Sykiotis et al., 2011, Pang et al., 2014, Karim et al., 2015, Steinbaugh et al., 2015). However, the role of CncC in modulating the triglyceride phenotype of lithium remains to be explored. Overall, our observations strongly suggest that lithium is a pro-longevity drug capable of extending lifespan at low doses independent of sex and genetic background, and under a variety of dietary conditions.

### Lithium Toxicity, Hormesis, and Stress Resistance

In humans, the therapeutic window for lithium treatment of bipolar disorder lies between 0.5 and 1 mM in serum, whereas concentrations of 1.5 mM and above severely increase the risk of tissue damage (Malhi and Tanious, 2011). Previous work in *Drosophila* suggests that the dose range at which we observed lifespan extension (0.5–25 mM) translates to *Drosophila* tissue concentrations below 0.5 mM (Dokucu et al., 2005). As previously reported for *C*. *elegans* and *Drosophila* (McColl et al., 2008, Zhu et al., 2015), concentrations above 50 mM were highly toxic.

Drug interventions to promote healthy lifespan are less likely to have side effects if started late in life (Castillo-Quan et al., 2015). Only a handful of drugs approved by the US Food and Drug Administration, namely rapamycin, metformin, and the Ras inhibitor trametinib, induce lifespan extension when commenced at later ages in model organisms (Harrison et al., 2009, Cabreiro et al., 2013, Martin-Montalvo et al., 2013, Slack et al., 2015). We found that lithium extends lifespan when first administered in mid-late life. In humans, long-term treatment with lithium for psychiatric disorders is associated with progressive and permanent renal damage (Malhi and Tanious, 2011). We showed that short treatment periods in *Drosophila*, 15 days during early adulthood, are sufficient to prolong life. Taken together, our data suggest that when testing lithium as a pro-longevity drug in mammals, lower doses than those used in psychiatric disorders are likely to be sufficient, and other strategies such as alternate-day dosing or transient treatment periods (either early or late in life), may be sufficient to reduce undesirable side effects and maximize the potential health benefits.

Interestingly, doses of lithium that shortened lifespan were protective against certain forms of xenobiotic stress. In vitro studies in mammalian cells have shown that lithium, and other GSK-3 inhibitors, protect against cell death caused by rotenone-induced oxidative stress (Lai et al., 2006), glutamate excitotoxicity, and H_2_O_2_ (Schäfer et al., 2004). This is likely mediated through a hormetic response (Suganthi et al., 2012), in this case orchestrated by NRF-2 activation. We observed that while simultaneous activation of CncC by loss of Keap1 and lithium treatment is additive and confers greater stress resistance to xenobiotics, the threshold for lifespan extension is perhaps considerably lower. A similar situation has been observed in *C*. *elegans* in which strong activation of the endoplasmic reticulum unfolded protein response conferred stress resistance benefits, while shortening lifespan (Taylor and Dillin, 2013). Our findings thus suggest that while NRF-2 activation either by loss of Keap1 or inhibition of GSK-3 is beneficial for longevity and stress resistance, at low levels of activation, stronger induction is detrimental for lifespan. This suggests that the hormetic benefits of lithium are more likely to occur at low levels under basal non-stress conditions (Calabrese, 2013). Hence, when testing for GSK-3 inhibitors or NRF-2 activators in modulating animal (and especially mammalian) aging, the degree of NRF-2 activation within the hormetic curve will determine positive or negative longevity outcomes. Future work studying the convergence of the salutary and damaging effects of lithium will aid in understanding to what extent the molecular mechanisms are shared (Calabrese and Mattson, 2011, Calabrese et al., 2013, Epel and Lithgow, 2014). Additionally, our microarray analysis was performed in heads and thoraces; therefore, it remains to be explored to what extent systemic or localized activation of NRF-2 modulates longevity, stress resistance, and lipid metabolism at the tissue level (Douglas et al., 2015).

### GSK-3 and NRF-2 as Drug Targets for Aging

Complete absence of GSK-3 in *C*. *elegans*, *Drosophila*, and mice shortens lifespan or prevents development (Hoeflich et al., 2000, McColl et al., 2008, Bourouis, 2002), while moderate inhibition has been associated with most of its positive effects (Avrahami et al., 2013). GSK-3 is upregulated in many disease states, including neurodegeneration, diabetes, inflammatory conditions, and some cancers (Takahashi-Yanaga, 2013). We have shown that adult-specific genetic manipulation of the fly ortholog of GSK-3, Sgg, affects longevity. Downregulation of Sgg prolonged lifespan and lithium was unable to further extend the lifespan, suggesting that lithium and inhibition of Sgg act through a common molecular pathway to extend lifespan.

In *C*. *elegans* and mammalian cells, GSK-3 directly interacts with NRF-2 to repress its activity, independently of Keap1 (An et al., 2005, Salazar et al., 2006, Rojo et al., 2008, Rada et al., 2012). Therefore, we hypothesized that lithium might act via Sgg/GSK-3, to de-repress CncC, the fly ortholog of NRF-2 and activate the oxidative and xenobiotic stress transcriptional signature (An et al., 2005, Hayes et al., 2015), which in turn would induce a CncC/NRF-2-dependent protective response (Jones et al., 2015, Blackwell et al., 2015). GO enrichment analysis identified a transcriptional signature that indeed suggested that lithium acts via CncC/NRF-2. CncC activity was indispensable for the lifespan extension conferred by lithium. In keeping with our results, work in rodents and mammalian cell lines has shown that lithium treatment and GSK-3 inhibition activate NRF-2 (Lee et al., 2014, Rizak et al., 2014). Because activation of CncC/NRF-2 modulates longevity in *C*. *elegans* and *Drosophila* (Tullet et al., 2008, Sykiotis and Bohmann, 2008, Ewald et al., 2015), our results provide evidence that GSK-3 is a viable therapeutic target to promote longevity via activation of NRF-2.

To date, the only GSK-3 inhibitor approved for human use is lithium (Williams and Harwood, 2000, Meijer et al., 2004, Martinez et al., 2011). However, researchers and pharmaceutical companies have developed more selective GSK-3 inhibitors, some of which have already entered the early stages of clinical trials for obesity, Alzheimer disease, and progressive supranuclear palsy (Eldar-Finkelman and Martinez, 2011). Our results call for a reassessment of the potential use of GSK-3 inhibitors and NRF-2 activators as potential anti-aging compounds.

## Experimental Procedures

### Fly Stocks and Husbandry

The *w*^*1118*^ stock was obtained from Bloomington *Drosophila* Stock Center. The control white *Dahomey* (*w*^*Dah*^) stock has been maintained in large population cages with overlapping generations since 1970. The *w*^*Dah*^ stock was initially derived by incorporation of the *w*^1118^ mutation into the outbred *Dahomey* background by backcrossing (Bass et al., 2007). Further details concerning fly mutants can be found in the Supplemental Experimental Procedures.

### Lithium Treatment

LiCl (Sigma) or NaCl (Sigma) were dissolved in ddH_2_O at a concentration of 5 M before supplementing to the medium. Equivalent volumes of vehicle were supplemented to the medium to compensate for dilution.

### Dietary Restriction Protocol

The DR protocol was performed as described previously (Bass et al., 2007).

### Statistical Analyses

Statistical analyses were performed using Excel, GraphPad Prism, or JMP software version 9 (SAS Institute). Survival experiments were analyzed using log rank test. Other data were tested by one-way analyses of variance (ANOVA) and planned comparisons of means were made using Tukey-Kramer HSD test. Cox proportional hazards analysis was performed to compare interactions for survival.

## Author Contributions

J.I.C.-Q. and I.B. conceived the experiments. J.I.C.-Q., I.B., L.L., K.J.K., L.S.T., T.N., and F.K. performed the experiments. D.K.I. analyzed the microarray data. C.S. and I.B. contributed reagents. J.I.C.-Q., I.B., J.T., J.H., and L.P. supervised experiments/project. J.I.C.-Q. and L.P. wrote the manuscript. All authors approved the final submission.

## Accession Numbers

The accession number reported for the microarray data in this paper is ArrayExpress: E-MTAB-3809.

## Supplemental Information

Document S1. Supplemental Experimental Procedures and Figures S1–S7

Document S2. Article plus Supplemental Information
