# Supplementary material for: Lithium Promotes Longevity through GSK3/NRF2-Dependent Hormesis
Source: Cell Rep. 2016 Apr 7;15(3):638–50. doi: 10.1016/j.celrep.2016.03.041 (PMC4850359; doi:10.1016/j.celrep.2016.03.041)
Supplement: Document S1. Supplemental Experimental Procedures and Figures S1–S7 [file mmc1.pdf]

Cell Reports, Volume *15*

Supplemental Information

Lithium Promotes Longevity

through GSK3/NRF2-Dependent Hormesis

Jorge Iván Castillo-Quan, Li Li, Kerri J. Kinghorn, Dobril K. Ivanov, Luke S. Tain, Cathy Slack, Fiona Kerr, Tobias Nespital, Janet Thornton, John Hardy, Ivana Bjedov, and Linda Partridge

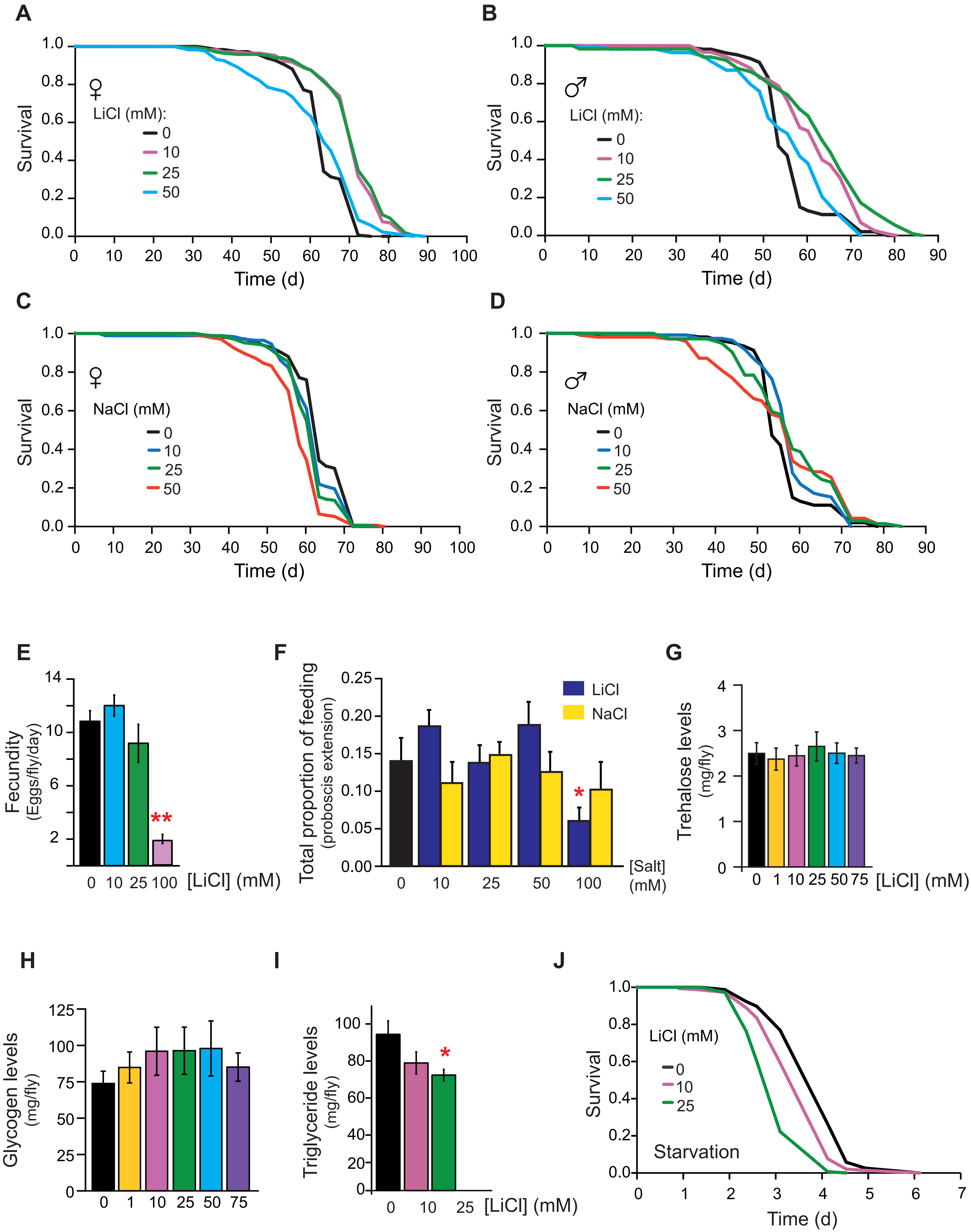
A
Survival
♀
LiCl (mM):
0
10
25
50
Time (d)
B
♂
LiCl (mM):
C
♀
NaCl (mM)
D
♂
NaCl (mM)
E
Fecundity (Eggs/fly/day)
**
[LiCl] (mM)
F
Total proportion of feeding (proboscis extension)
LiCl
NaCl
*
[Salt] (mM)
G
Trehalose levels (mg/fly)
[LiCl] (mM)
H
Glycogen levels (mg/fly)
I
Triglyceride levels (mg/fly)
*
[LiCl] (mM)
J
LiCl (mM)
Starvation
Survival
Time (d)

SUP FIGURE 1

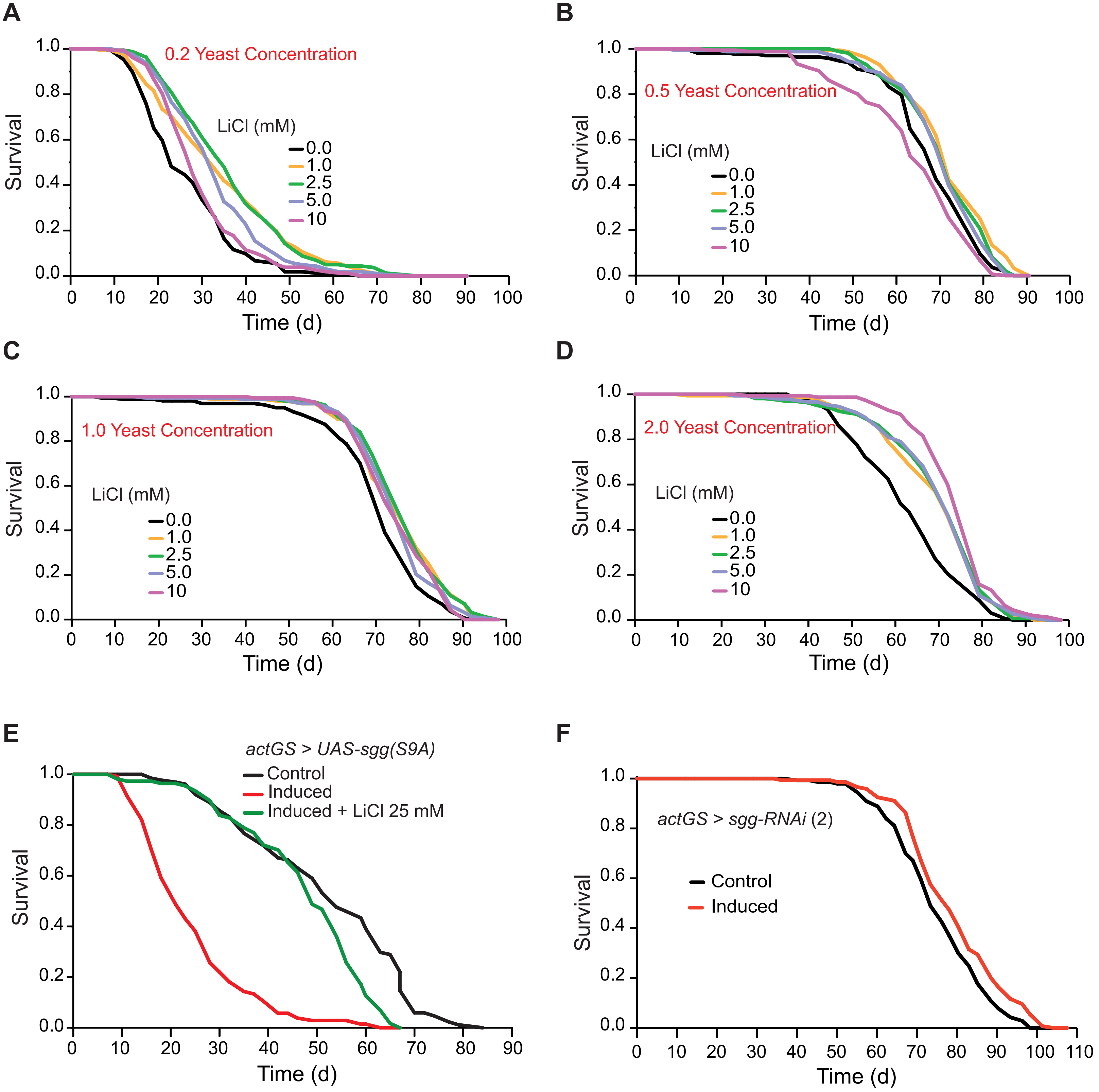
A
0.2 Yeast Concentration
LiCl (mM)
0.0
1.0
2.5
5.0
10
Survival
Time (d)
B
0.5 Yeast Concentration
C
1.0 Yeast Concentration
D
2.0 Yeast Concentration
E
actGS > UAS-sgg(S9A)
Control
Induced
Induced + LiCl 25 mM
F
actGS > sgg-RNAi (2)
Control
Induced

SUP FIGURE 2

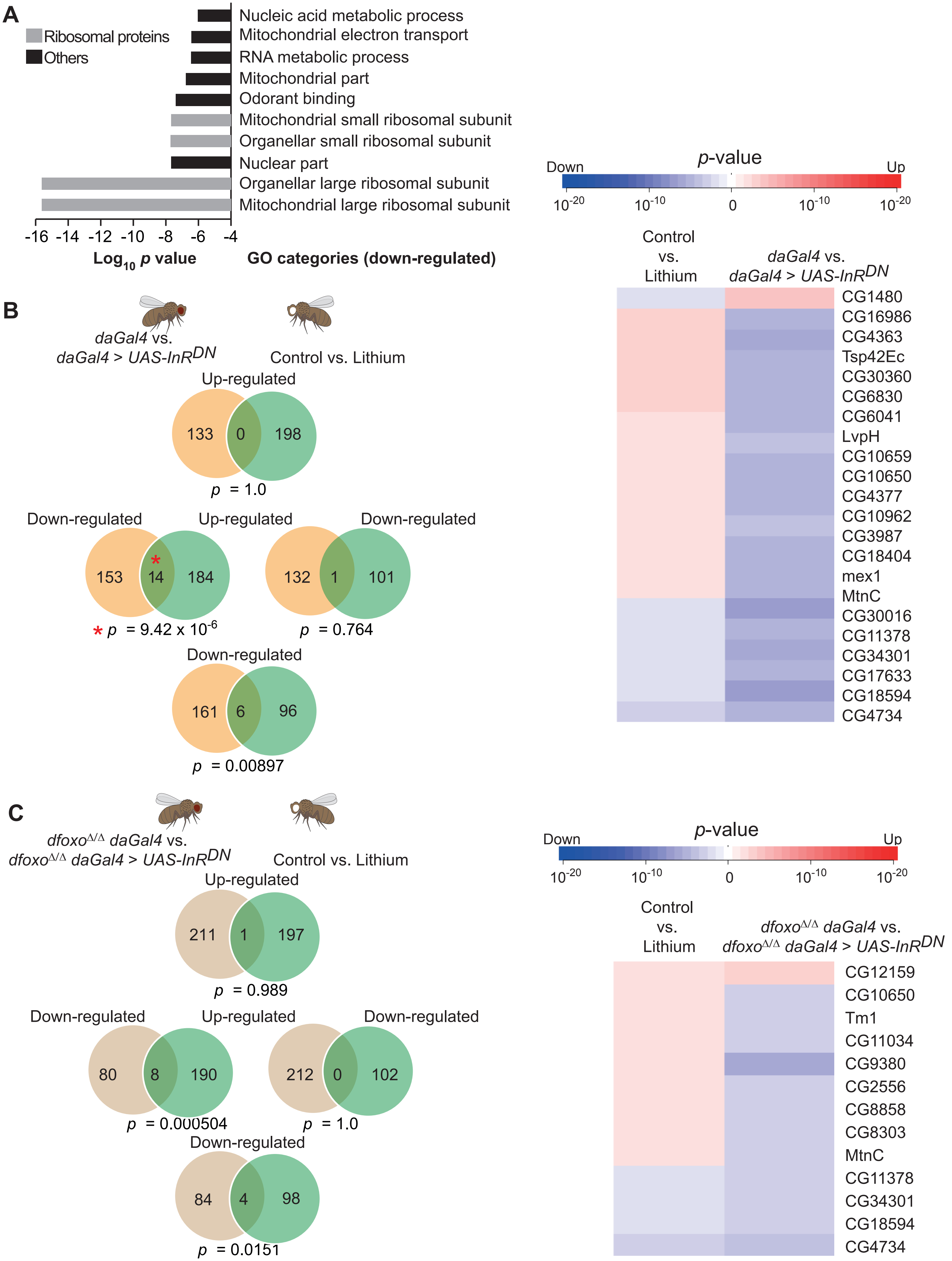
A
Ribosomal proteins
Others
Nucleic acid metabolic process
Mitochondrial electron transport
RNA metabolic process
Mitochondrial part
Odorant binding
Mitochondrial small ribosomal subunit
Organellar small ribosomal subunit
Nuclear part
Organellar large ribosomal subunit
Mitochondrial large ribosomal subunit
-16 -14 -12 -10 -8 -6 -4
Log_{10} p value
GO categories (down-regulated)
p-value
Down
Up
10^{-20} 10^{-10} 0 10^{-10} 10^{-20}
Control vs. Lithium
daGal4 vs. daGal4 > UAS-InR^DN
CG1480
CG16986
CG4363
Tsp42Ec
CG30360
CG6830
CG6041
LvpH
CG10659
CG10650
CG4377
CG10962
CG3987
CG18404
mex1
MtnC
CG30016
CG11378
CG34301
CG17633
CG18594
CG4734
B
daGal4 vs. daGal4 > UAS-InR^DN
Control vs. Lithium
Up-regulated
133
0
198
p = 1.0
Down-regulated
Up-regulated
Down-regulated
153
*
14
184
132
1
101
* p = 9.42 x 10^{-6}
p = 0.764
Down-regulated
161
6
96
p = 0.00897
C
dfoxo^Δ/Δ daGal4 vs. dfoxo^Δ/Δ daGal4 > UAS-InR^DN
Control vs. Lithium
Up-regulated
211
1
197
p = 0.989
Down-regulated
Up-regulated
Down-regulated
80
8
190
212
0
102
p = 0.000504
p = 1.0
Down-regulated
84
4
98
p = 0.0151
p-value
Down
Up
10^{-20} 10^{-10} 0 10^{-10} 10^{-20}
Control vs. Lithium
dfoxo^Δ/Δ daGal4 vs. dfoxo^Δ/Δ daGal4 > UAS-InR^DN
CG12159
CG10650
Tm1
CG11034
CG9380
CG2556
CG8858
CG8303
MtnC
CG11378
CG34301
CG18594
CG4734

SUP FIGURE 3

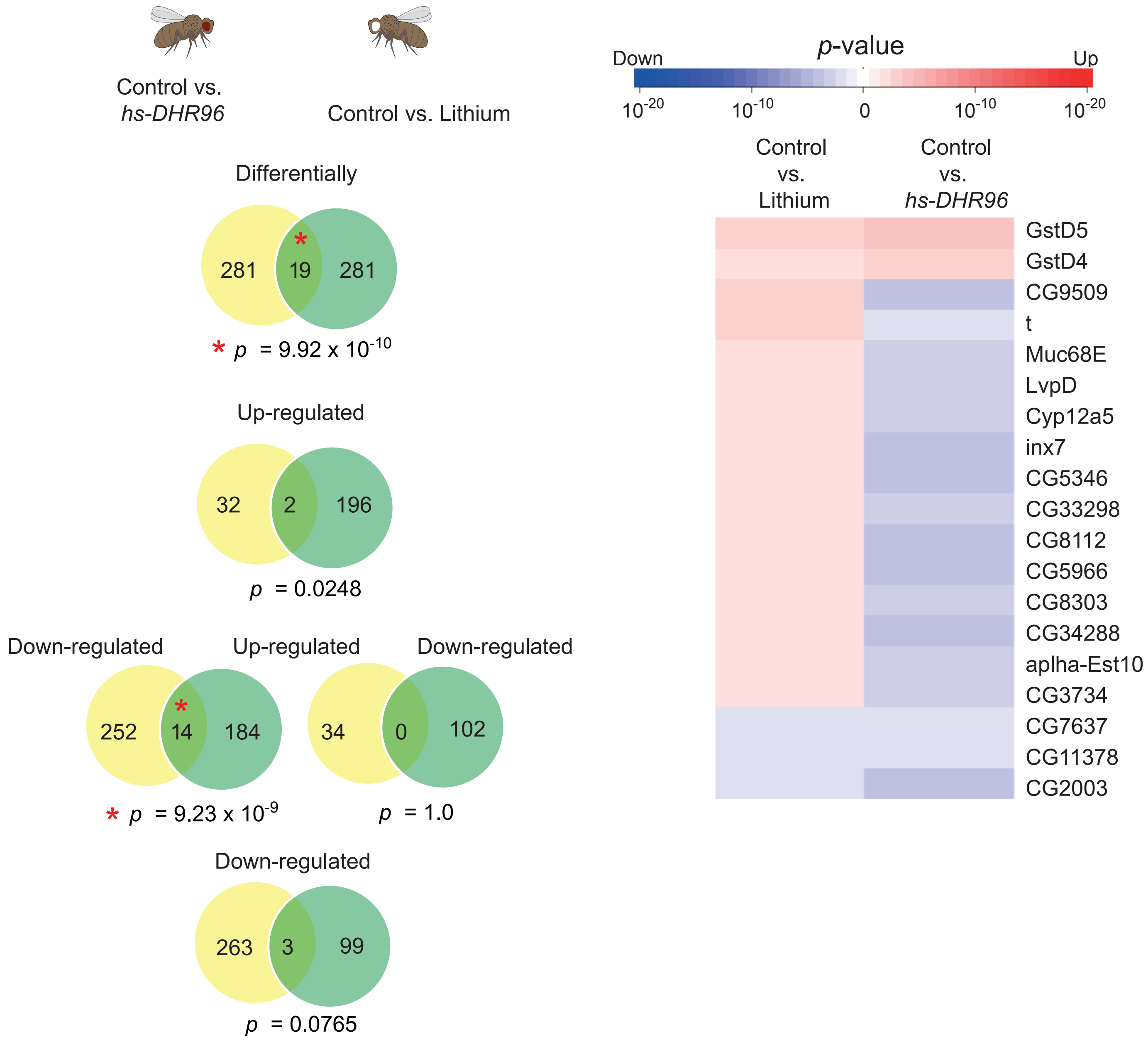
Control vs.
hs-DHR96
Control vs. Lithium
Differentially
281
19
281
* p = 9.92 x 10-10
Up-regulated
32
2
196
p = 0.0248
Down-regulated
Up-regulated
252
14
184
* p = 9.23 x 10-9
Up-regulated
Down-regulated
34
0
102
p = 1.0
Down-regulated
263
3
99
p = 0.0765
p-value
Down
Up
10-20
10-10
0
10-10
10-20
Control
vs.
Lithium
Control
vs.
hs-DHR96
GstD5
GstD4
CG9509
t
Muc68E
LvpD
Cyp12a5
inx7
CG5346
CG33298
CG8112
CG5966
CG8303
CG34288
aplha-Est10
CG3734
CG7637
CG11378
CG2003

SUP FIGURE 4

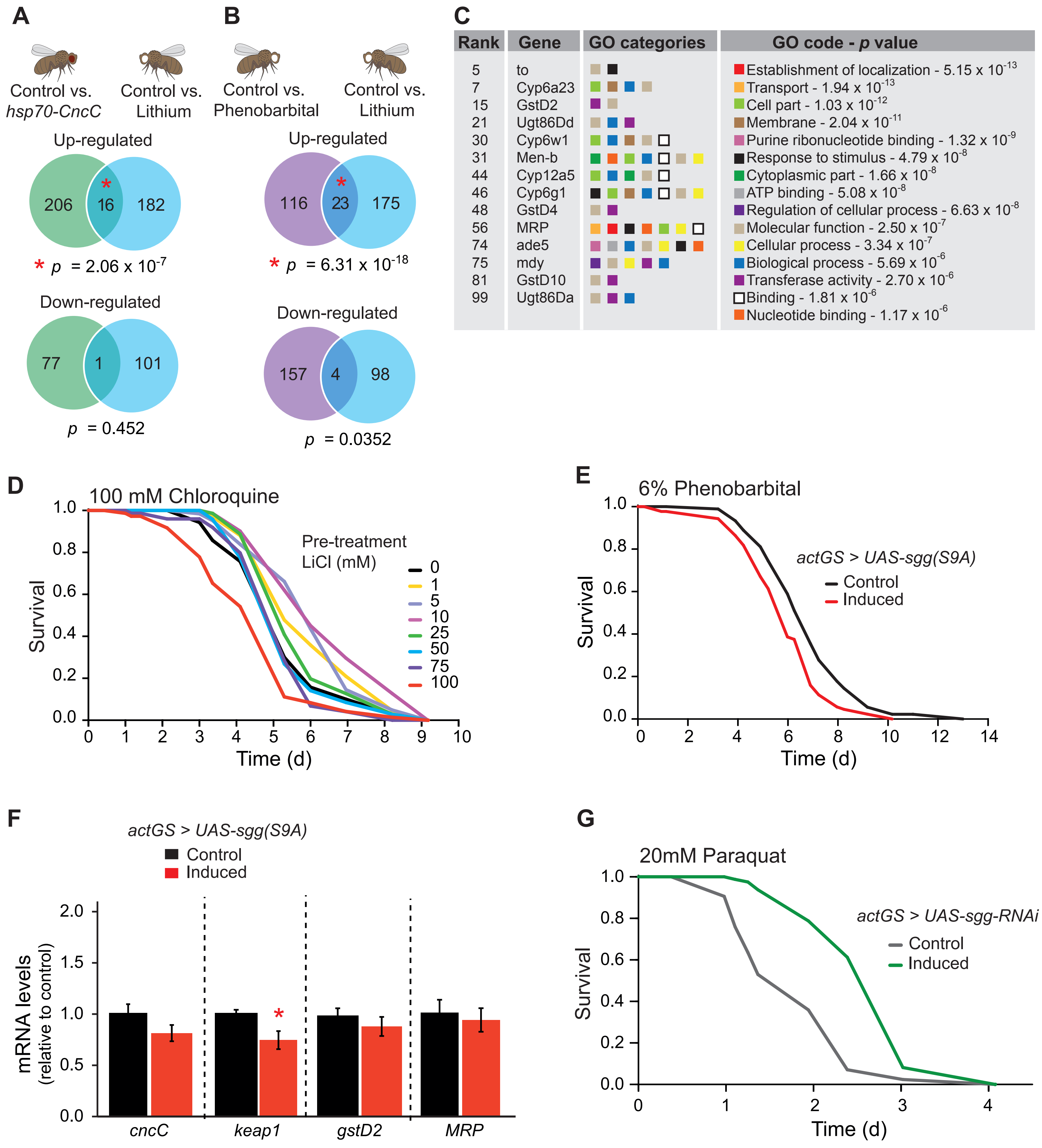
A
Control vs.
hsp70-CncC
Control vs.
Lithium
Up-regulated
206
16
182
* p = 2.06 x 10-7
Down-regulated
77
1
101
p = 0.452
B
Control vs.
Phenobarbital
Control vs.
Lithium
Up-regulated
116
23
175
* p = 6.31 x 10-18
Down-regulated
157
4
98
p = 0.0352
C
Rank
Gene
GO categories
GO code - p value
5 to
7 Cyp6a23
15 GstD2
21 Ugt86Dd
30 Cyp6w1
31 Men-b
44 Cyp12a5
46 Cyp6g1
48 GstD4
56 MRP
74 ade5
75 mdy
81 GstD10
99 Ugt86Da
Establishment of localization - 5.15 x 10-13
Transport - 1.94 x 10-13
Cell part - 1.03 x 10-12
Membrane - 2.04 x 10-11
Purine ribonucleotide binding - 1.32 x 10-9
Response to stimulus - 4.79 x 10-8
Cytoplasmic part - 1.66 x 10-8
ATP binding - 5.08 x 10-8
Regulation of cellular process - 6.63 x 10-8
Molecular function - 2.50 x 10-7
Cellular process - 3.34 x 10-7
Biological process - 5.69 x 10-6
Transferase activity - 2.70 x 10-6
Binding - 1.81 x 10-6
Nucleotide binding - 1.17 x 10-6
D
100 mM Chloroquine
Pre-treatment
LiCl (mM)
0
1
5
10
25
50
75
100
Survival
Time (d)
E
6% Phenobarbital
actGS > UAS-sgg(S9A)
Control
Induced
Survival
Time (d)
F
actGS > UAS-sgg(S9A)
Control
Induced
mRNA levels
(relative to control)
cncC
keap1
gstD2
MRP
G
20mM Paraquat
actGS > UAS-sgg-RNAi
Control
Induced
Survival
Time (d)

SUP FIGURE 5

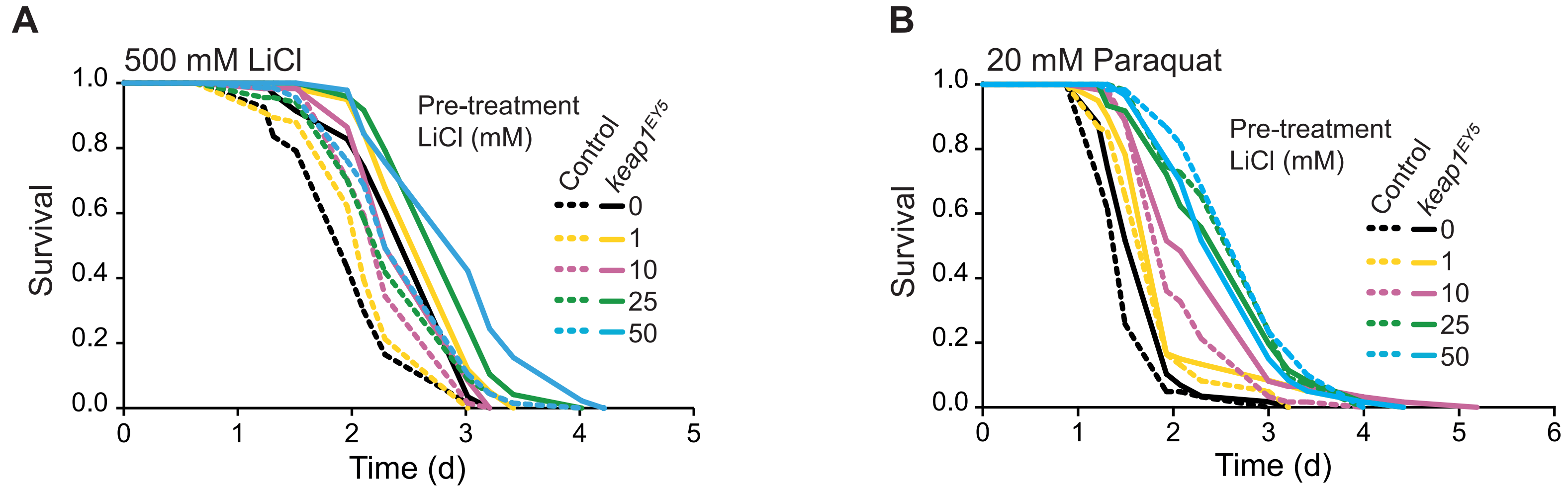

A
500 mM LiCl
Survival
Time (d)
Pre-treatment
LiCl (mM)
Control
keap1EY5
0
1
10
25
50
B
20 mM Paraquat
Survival
Time (d)
Pre-treatment
LiCl (mM)
Control
keap1EY5
0
1
10
25
50

SUP FIGURE 6

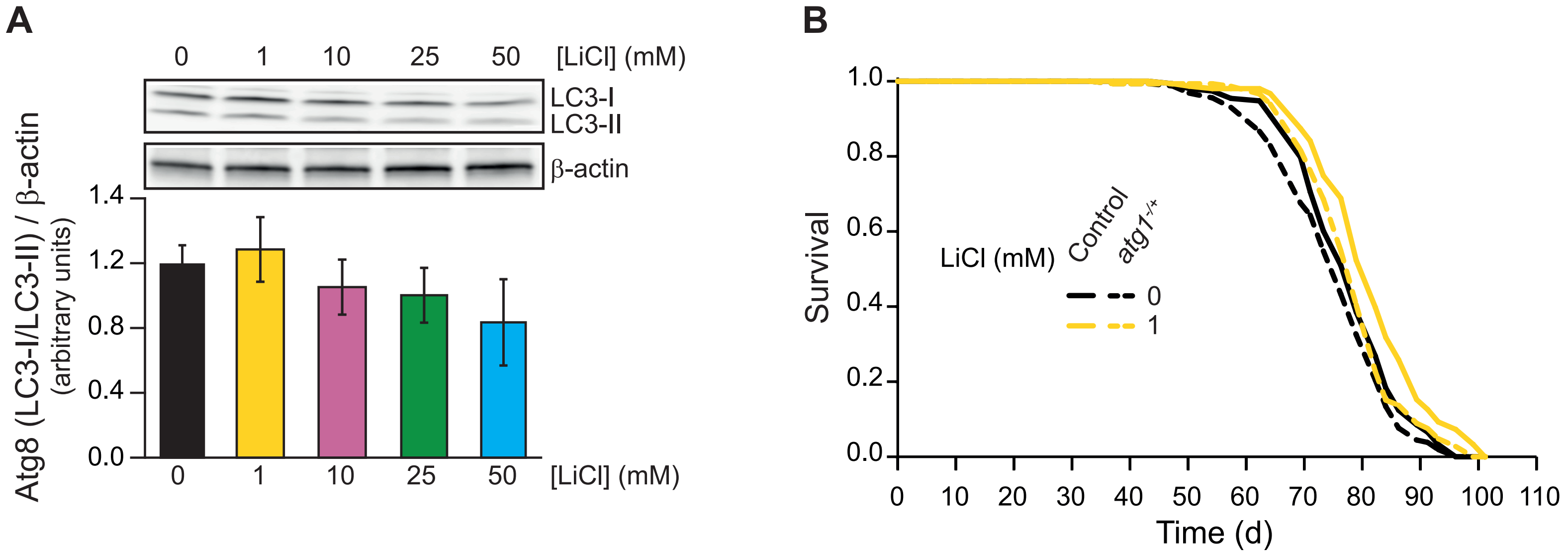
A
0
1
10
25
50
[LiCl] (mM)
LC3-I
LC3-II
β-actin
Atg8 (LC3-I/LC3-II) / β-actin
(arbitrary units)
1.4
1.2
0.8
0.4
0.0
0
1
10
25
50
[LiCl] (mM)
B
1.0
0.8
0.6
0.4
0.2
0.0
Survival
LiCl (mM)
Control
atg1-/+
0
1
0
10
20
30
40
50
60
70
80
90
100
110
Time (d)

SUP FIGURE 7

Supplemental Figure Legends

Figure S1. Lithium modulation of lifespan and metabolism.

(A) Lithium extended the lifespan of female *w*^{1118} flies (n = 150 flies per condition) at concentrations of 10 and 25 mM (+13% and +13-18% median and maximum lifespan extension; $p < 0.001$), but not at 50 mM ($p > 0.05$). Similar concentrations of NaCl did not extend lifespan (see Fig. S1C).

(B) Lithium extended lifespan of male *w*^{1118} flies (n = 150 flies per condition) at concentrations of 10 and 25 mM ($p < 0.001$, log-rank test). 10 mM lithium extended median lifespan by 18.5% and maximum lifespan by ~4%, while 25 mM lithium extended median lifespan by 23% and maximum by 13.5%. The higher dose of 50 mM lithium did not significantly extend lifespan ($p > 0.05$). Similar concentrations of NaCl did not extend lifespan (see Fig. S1D).

(C) Concentrations of NaCl similar to those of lithium that promote longevity (see Fig. 1A) did not extend lifespan of female *w*^{1118} flies (n = 150 flies per condition). 50 mM significantly shortened lifespan ($p < 0.05$) Note that this experiment was run in parallel to that in Fig. S1A, hence the lifespan data for controls (LiCl 0 mM) is the same.

(D) Concentrations of NaCl similar to those of lithium that promote longevity did not extend the lifespan of male *w*^{1118} flies (n = 150 flies per condition). Note that this experiment was run in parallel to that in Fig. S1B, hence the lifespan data for controls (LiCl 0 mM) is the same.

(E) Female *w*^{1118} flies treated with 10 or 25 mM lithium for 15 days did not show significant changes in fecundity, while flies treated with 100 mM lithium showed a significant ($p < 0.01$) reduction. N = 150 females per condition.

(F) Lithium or NaCl –treated flies for 15 days did not show changes in feeding behavior, except for 100 mM lithium which significantly reduced it ($p < 0.05$). N = 50 flies per condition.

(G) There were no significant differences in whole body trehalose levels after 15 days of lithium treatment of female w^{Dah} flies. Bars represent means ± SEM of triplicates of 6 biological repeats of 5 flies.

(H) There were no significant differences in whole body glycogen after 15 days of lithium treatment of female w^{Dah} flies. Bars represent means ± SEM of triplicates of 6 biological repeats of 5 flies.

(I) Triglycerides in female w^{1118} flies. Whole body triglyceride levels were reduced in flies after 15 days of lithium treatment compared with untreated controls. Bars represent medians of 6 replicas of 5 flies per condition ± SEM, * $p < 0.05$.

(J) Female w^{1118} flies pre-treated with increasing concentrations of lithium for 15 days showed a dose-dependent sensitivity to starvation ($p < 0.01$; n = 75 flies per condition).

Figure S2. Interactions of lithium with DR and GSK-3.

(A) Lithium increased lifespan under semi-starvation (0.2x yeast; n = 160 flies per condition). Doses from 1 to 5 mM lithium significantly extended lifespan ($p < 0.001$). 10 mM lithium treatment did not significantly change survival ($p > 0.05$).

(B) Doses of 1 and 2.5 mM lithium extended lifespan under semi-starvation (0.5x yeast; $p < 0.05$), while 10 mM lithium significantly shortened lifespan ($p < 0.05$). N = 160 flies per condition.

(C) All doses of lithium tested increased lifespan under the yeast concentration that maximized lifespan (1.0x yeast). $p < 0.001$ for 1 and 2.5 mM lithium versus control, and $p < 0.01$ for 5 and 10 mM; n = 160 flies per condition.

(D) All lithium concentrations tested extended lifespan under fully fed conditions (2.0x yeast; $p < 0.001$).

(E) Ubiquitous expression of a constitutively active *sgg* (*sgg(S9A)*) shortened lifespan ($p < 0.001$), and this shortening was almost completely reversed by 25 mM lithium ($p < 0.001$ for induced not treated and $p < 0.05$ for induced treated vs control). N = 150 flies per condition.

(F) A second RNAi line against *sgg* significantly extended lifespan ($p < 0.01$) when expressed ubiquitously using the actGS driver induced 2 days post-eclosion. N = 150 flies per condition.

Figure S3. Bioinformatics analyses of lithium-treated flies and interaction with IIS down-regulation.

(A) Top 10 of most significantly changed GO categories of down-regulated genes after lithium treatment.

(B) Venn diagrams and heatmap showing the overlap of genes differentially changed by lithium and the expression of an insulin receptor dominant negative (Alic et al., 2011). The only significant overlap over the Bonferroni corrected *p* value ($p < 0.0001$) was for genes down-regulated by IIS down-regulation and genes up-regulated by lithium ($p = 9.42^{-6}$).

(C) Venn diagrams and heatmap showing the overlap of genes differentially changed by lithium and expression of an insulin receptor dominant negative in a *dfoxo* null background (Alic et al., 2011). No significant overlap over the Bonferroni corrected *p* value ($p < 0.0001$) was detected in any direction.

Figure S4. Bioinformatic analyses of the interaction of lithium and DHR96.

Venn diagrams and heatmap showing the overlap of genes differentially changed by lithium and over-expression of *DHR96* (King-Jones et al., 2006). We detected a significant overlap over the Bonferroni corrected *p* value ($p < 0.0001$) for differentially expressed genes by both treatments. However, genes down-regulated by DHR96 significantly overlapped with those up-regulated by lithium treatment ($p = 9.23^{-10}$). No other significant overlap was detected in any direction.

Figure S5. Interactions of lithium transcriptional response with phenobarbital treatment and *cncC* over-expression.

(A) Genes up-regulated by *cncC* over-expression (Misra et al., 2011) overlapped with those up-regulated by lithium treatment ($p = 2.06 \times 10^{-7}$, Fisher's exact test). Down-regulated genes by c*ncC* over-expression (Misra et al., 2011) and lithium did not significantly overlap ($p = 0.452$, Fisher's exact test).

(B) Lithium and phenobarbital treatment (Misra et al., 2011) up-regulated an overlapping set of genes ($p = 6.31 \times 10^{-18}$, Fisher's exact test), but did not change a similar set of genes ($p = 0.0352$; statistical value was considered when $p < 0.0001$, Bonferroni correction for multiple comparisons) at the down-regulated level (Misra et al., 2011).

(C) GO category classification for up-regulated genes involved in detoxification commonly changed by lithium treatment and *cncC* over-expression.

(D) Pretreatment with 1 to 10 mM lithium significantly protected ($p < 0.05$) flies against the xenobiotic chloroquine. N = 75 flies per condition

(E) Over-expression of the S9A *sgg* mutant significantly reduced survival when stressed with phenobarbital ($p < 0.05$, log rank test). N= 75 flies per condition.

(F) Over-expression of *sgg(S9A)* significantly reduced *keap1* mRNA levels ($p < 0.05$), while non-significant trends were detected for *cncC*, *gstD2* and *MRP* mRNA levels ($p > 0.05$).

(G) RNAi-mediated down-regulation of *sgg* protected against paraquat ($p < 0.001$). N = 75 flies per condition.

Figure S6. Interaction of lithium with Keap1.

(A) Heterozygous loss of *keap1* ($keap1^{EY5}$) significantly protected against toxic concentrations of lithium ($p < 0.001$), and the combination of genetic and pharmacological activation of CncC further protected flies ($p < 0.001$ for 25 and 50 mM lithium treated mutants vs mutant or the same doses in the control flies). N = 75 flies per condition.

(B) The $keap1^{EY5}$ mutant significantly protected against paraquat-induced stress compared with control flies ($p < 0.01$), and potentiated the effect on lifespan of lithium pre-treatment ($p < 0.001$). N = 75 flies per condition.

Figure S7. Interaction of lithium with autophagy.

(A) 15 days of lithium treatment at several concentrations did not induce autophagy as measured by Atg8 (LC3) levels. There was a slight but non-significant reduction in the levels of Atg8**.**

(B) Lithium extended lifespan of flies with heterozygous loss of atg1. N= 120 flies per condition.

Supplemental Experimental Procedures

Fly Stocks and Husbandry

Stocks and experiments were kept at 25˚C and 65% humidity on a 12:12 light:dark cycle using standard sugar/yeast (SY) medium (15gL^{-1} agar, 50gL^{-1} sucrose, 100gL^{-1} autolyzed yeast, 100 gL^{-1} nipagin and 3 mL L^{-1} propionic acid). *daGS* was a gift from Veronique Monnier (Tricoire et al., 2009). The following stocks were obtained from Bloomington *Drosophila* Stock Center: *UAS-sgg*, *UAS-sgg(S9A)*. *UAS-sgg-RNAi w*as obtained from Vienna *Drosophila* Resource Center. *UAS-sgg-RNAi(2)* is part of the Transgenic RNAi project at Harvard Medical School and obtained from Bloomington. *actGS* (Poirier et al., 2008), *atg1*$^{-/+}$ (*atg*^{1}) (Lee et al., 2007) and all previously mentioned lines were backcrossed at least 6 times into the *w*^{Dah} background. *keap1*^{EY5}, *UAS-keap1*, *Gst-D-eGFP*, and *UAS-cncC-RNAi* were obtained from the Bohmann laboratory (Sykiotis and Bohmann, 2008). The *keap1* deletion (*keap1*^{Del}) was generated by P-element mediated male recombination using the P-element insertion line, Keap1[EY02632]. The resulting lines were screened by PCR for deletions that removed Keap1 coding sequence. Keap1-gDNA2 TTACTATTCCACCGTGTTGTG, Keap1-gDNA3 CCGCTCATTCCGTATGAAG. All *keap1*/*cncC* fly lines were backcrossed at least 6 times (except for the *yw; UAS-cncC-RNAi* that was used as a hybrid) into the *w*^{1118} background and then crossed into *w*^{Dah} females for experimental analyses. Gene induction (Osterwalder et al., 2001) was obtained by supplementing food with RU486 (Sigma) at 200 μM (stock prepared at 100 mM in 100% ethanol).

Lifespan Experiments

'Egg squirt' protocols were undertaken to ensure that all experimental flies were raised at similar larval densities (~300 eggs per bottle containing 70 mL of food). Flies were allowed to lay eggs for less than 24 hours on grape medium plates, with live yeast paste to encourage egg-laying. The eggs were collected from the plate by washing with phosphate buffered

saline (PBS) solution and collected into falcon tubes. The eggs were then allowed to settle to the bottom of the tube. Using a 100 µL Gilson pipette ~15-18 µL of egg suspension was dispensed into 200 mL glass bottles containing 70 mL SY medium. Following eclosion flies were transferred to fresh food for a 48 hr mating period. Under CO_2 anesthesia flies were divided by sex with 15-20 flies per vial. Flies were transferred to fresh vials of food three times a week throughout life. The number of dead flies found during each transfer was recorded. Lifespan curves were analyzed using a log-rank test. Maximum lifespans were calculated as the median of the last surviving 10% of the population.

Behavioral measurements

Feeding behavior was assessed by analyzing the proboscis extension of 10 vials of five flies each. The feeding behaviour assay relies on the fly proboscis extension (Wong et al., 2009). The number of flies engaged in proboscis extension was recorded and plotted as a function of observed versus estimated. To assess fecundity female flies were let lay eggs for a period of ~24 hours in vials containing standard medium. Vials containing eggs were frozen and kept at -20°C for a short time until eggs were counted (Grandison et al., 2009). Locomotor ability was assessed through the negative geotaxis paradigm which consists of a climbing assay in which 10-15 flies were placed in a 35 cm column (1.5 cm diameter) with a conic bottom end. Flies were tapped down and observed during 45 seconds. The column was separated into three areas: top, middle and bottom by two lines; one was 10 cm from top and the other was 3 cm from bottom). After 45 seconds flies located in each of these three sections were recorded into scoring sheets. Each column was evaluated three times to minimize trial error and a minimum of three vials per genotype/condition was evaluated in order to be able to perform statistical analyses. The recorded scoring showed the mean number of flies in total (ntot), at top (ntop), and at bottom (nbottom), which allowed obtaining a performance index. The performance index (PI) was calculated as ½ (ntot + ntop – nbottom / ntot). The performance index was plotted against time per each genotype and

condition. Results were analysed with two-way ANOVA and Tukey post-hoc (Rogers et al., 2012; Kerr et al., 2011).

Stress Assays

Phenobarbital, chloroquine and paraquat were obtained from Sigma. All were dissolved directly into the sugar/agar medium similarly prepared as the normal fly medium without yeast. No LiCl treatment was provided during the stress period. For starvation assays flies were maintained on a pure 1.5% agar medium.

Triglyceride and Storage Carbohydrate Measurements

To quantify triglyceride levels a colorimetric assay kit by Thermo Scientific was used (Bjedov et al., 2010; Kinghorn et al., 2015); 6 replicas of 5 female flies were homogenized in 0.05% Tween 20 and incubated for 5 minutes at 70ºC. Samples were then centrifuged for 5 minutes at 7000 rpm. The supernatant was transferred to fresh Eppendorfs and centrifuged for 10 minutes at maximum speed. For each sample, 175 µL was transferred to a fresh Eppendorf. 10 µL of each sample was dispensed into a well on a 96-well plate, with each sample in triplicate. 200 µL of Thermo Infinity Triglycerides solution was added to each well and the plate was left to incubate at 37ºC for 10 mins, after which time the absorbance in each well was measured at 574 nm. The lipid standards were treated in the same way as the samples all throughout and were prepared as 7 serial dilutions in 0.05% Tween 20. These were 2, 1, 0.5, 0.25, 0.125, 0.0625 and 0 µg/µL triglyceride. Samples were normalized to protein levels using the Thermo Scientific bicinchoninic acid protein (BCA) assay. Whole fly glycogen and trehalose were measured in once-mated females after 15 days of treatment as previously described (Broughton et al., 2005; Slack et al., 2011).

Western blot analysis

10 flies were homogenized in 2X Laemmli loading buffer (100 mM Tris 6.8, 20% glycerol, 4% SDS) containing 5% β-mercaptoethanol and then boiled for 5 min. Approximately 40 μg of protein extract was loaded per lane. Proteins were separated on SDS polyacrylamide gels and transferred to a nitrocellulose membrane. Membranes were blocked in 5% milk TBST for 1 hour at room temperature, after which they were probed for primary antibodies diluted in 5% BSA TBST overnight at 4°C. The following primary antibodies were used: GFP (Cell Signaling Technologies #2955, 1:1000), anti-phospho-GSK3 (Cell Signaling Technologies #9331, 1:500), anti-GSK-3 (US Biologicals #G8170, 1:1000), anti-Atg-8 (custom made, 1:1000), β-actin (Abcam #ab4801, 1:5000). Appropriate HRP-conjugated secondary antibodies (Abcam) were used (1:12,000). Blots were developed using the ECL detection system and analyzed using ImageJ program (US National Institutes of Health).

RT-qPCR

Total RNA was extracted from 15 flies using Trizol (GIBCO) according to the manufacturer's instructions. The concentration of total RNA purified for each sample was measured using an *Eppendorf biophotometer*. 2 μg of total RNA was then subjected to DNA digestion using DNAse I (Ambion), immediately followed by reverse transcription using the Superscript II system (Invitrogen) with oligo(dT) primers. Quantitative PCR was performed using the PRISM 7000 sequence-detection system (Applied Biosystems), SYBR Green (Molecular Probes), ROX Reference Dye (Invitrogen), and Hot Star Taq (Qiagen, Valencia, CA) by following manufacturer's instructions. Each sample was analysed in triplicate using a*ct5c* as reference gene. Primers used were: *gstD2*-F CATCGCCGTCTATCTGGTGGA, *gstD2*-R GGCATTGTCGTACCACCTGG, *keap1-F* CAAGGAGTCGGAGATGTCG, *keap1-R* GTAGAGGATGCGTGACATGG, *cncC-F* GAGGTGGAAATCGGAGATGA, *cncC-R* CTGCTTGTAGAGCACCTCAGC, *MRP*-F ACTTTACGCCCTGCTT, *MRP*-R TCACGTTCAGCTTGTTCCAC, a*ct5C*-F GCAGCAACTTCTTCGTCACA, a*ct5C*-R CATCAGCCAGCAGTCGTCTA.

Microarray analyses

Once-mated w^{1118} female flies were pre-treated for 10 days with 10 mM LiCl and snap frozen in liquid nitrogen, after which heads and thoraces were separated. RNA extraction was performed as per manufacturer's guidelines using Trizol. RNA was DNase treated and checked for quality by BioRad Experion. RNA was processed to cRNA, labeled and hybridized to a single-color Affymetrix GeneChip Drosophila Genome 2.0 Array, following manufacturer's protocol. These experiments were performed by the Max Planck-Genome-centre Cologne, Germany (http://mpgc.mpipz.mpg.de/home/).

Differential expression analysis

Raw data (cel files) were processed to correct for probe-sequence biases, by using bioconductor's package gcrma (http://www.bioconductor.org) in R (http://www.r-project.org). Affymetrix's MicroArray Suite 5.0 (bioconductor's package affy) was used to determine present target transcripts (Schuster et al., 2007). Transcripts were deemed present if the p-value was <0.111 and absent otherwise. The raw data were summarized and normalized by using Robust Multichip Average (rma function, part of bioconductor's package affy (Gautier et al., 2004)). In order to identify differentially expressed genes a linear model was fitted and differential expression was assessed using the empirical Bayes moderated *t*-statistic as implemented in R's limma package (Ritchie et al., 2015). P-values were adjusted for multiple hypotheses testing by applying the Benjamini and Hochberg correction for false discovery rate. Summarized probe-sets were mapped to transcripts using R's package "drosophila2.db". Transcripts not mapping to any known or predicted genes were excluded from further analysis.

The raw data for the wild-type vs. wild-type+Lithium(10mM) are deposited in ArrayExpress (http://www.ebi.ac.uk/arrayexpress) with identifier E-MTAB-3809. The following freely available microarray datasets were used: control vs. phenobarbital (E-GEOD-30087); control

vs. *hsp70*-CncC (E-GEOD-30087); *daGal4* vs. *daGal4 > UAS-InR*^{DN} and *dfoxo*$^{\Delta/\Delta}$ *daGal4* vs. *dfoxo*$^{\Delta/\Delta}$ *daGal4 > UAS-InR*^{DN} (E-TABM-757); control vs. *hs-DHR96* (E-GEOD-5097).

Statistical significance of differential expression gene sets

Statistical significance of overlaps of genes in two microarray experiments was determined using fisher's exact test. To account for multiple hypotheses testing a p-value cut-off of $\leq 1.0 \times 10^{-05}$ was used. After correcting for multiple hypothesis testing in the wild-type vs. wild-type+Lithium(10mM) (Benjamini and Hochberg correction for false discovery rate as described above), there were only two genes that were above the significance threshold of 0.05, thus we used the top 300 genes within each experiment to calculate significance of overlap. These genes could be viewed as top up or down-regulated genes among all of the genes part of an experiment.

Gene Ontology analysis

The Wilcoxon rank sum test, as implemented in Catmap (Breslin et al., 2004), was used to perform functional analysis, that is significant enrichment of Gene Ontology categories. FlyBase (http://flybase.org) gene identifiers were mapped to Gene Ontology identifiers (FlyBase version FB2014_01). Ranks of genes were based on the p-value derived from the Bayes *t*-statistic for differential expression. To account for multiple hypothesis testing, an enrichment of GO terms was deemed statistically significant if the p-value derived from the wilcoxon rank sum test was $\leq 1.0 \times 10^{-05}$. Gene lists were sorted by log-fold change and p-value. For all microarray experiments two sets of lists were derived; a gene list comprising most differentially up-regulated (log-fold change > 0) genes at the top of the list and most differentially down-regulated genes (log-fold change < 0) at the bottom of the list (termed up-to-down) and *vice verse* (termed down-to-up). If a GO category was found to be statistically significant in the up-to-down list, this GO was referred to as up-regulated, i.e. a large enough proportion of the genes that are part of this GO category were found to be up-regulated or at

the top of the list. If a GO category was found to be statistically significant in the down-to-up list, this GO was referred to as down-regulated, i.e. a large enough proportion of the genes that are part of this GO category were found to be down-regulated or at the top of the list.

Supplemental References

Alic, N., Andrews, T. D., Giannakou, M. E., Papatheodorou, I., Slack, C., Hoddinott, M. P., Cochemé, H. M., Schuster, E. F., Thornton, J. M., and Partridge, L. (2011). Genome-wide dFOXO targets and topology of the transcriptomic response to stress and insulin signalling. Mol. Syst. Biol. *7*, 502.

Bjedov, I., Toivonen, J. M., Kerr, F., Slack, C., Jacobson, J., Foley, A., and Partridge, L. (2010). Mechanisms of life span extension by rapamycin in the fruit fly Drosophila melanogaster. Cell Metab. *11*, 35–46.

Breslin, T., Edén, P., and Krogh, M. (2004). Comparing functional annotation analyses with Catmap. BMC Bioinformatics *5*, 193.

Broughton, S. J., Piper, M. D. W., Ikeya, T., Bass, T. M., Jacobson, J., Driege, Y., Martinez, P., Hafen, E., Withers, D. J., Leevers, S. J., et al. (2005). Longer lifespan, altered metabolism, and stress resistance in Drosophila from ablation of cells making insulin-like ligands. Proc. Natl. Acad. Sci. U. S. A. *102*, 3105–3110.

Gautier, L., Cope, L., Bolstad, B. M., and Irizarry, R. A. (2004). Affy - Analysis of Affymetrix GeneChip data at the probe level. Bioinformatics *20*, 307–315.

Grandison, R. C., Wong, R., Bass, T. M., Partridge, L., and Piper, M. D. W. (2009). Effect of a standardised dietary restriction protocol on multiple laboratory strains of Drosophila melanogaster. PLoS One *4*, e4067.

Kerr, F., Augustin, H., Piper, M. D. W., Gandy, C., Allen, M. J., Lovestone, S., and Partridge, L. (2011). Dietary restriction delays aging, but not neuronal dysfunction, in Drosophila models of Alzheimer's disease. Neurobiol. Aging *32*, 1977–1989.

Kinghorn, K. J., Castillo-Quan, J. I., Bartolome, F., Angelova, P. R., Li, L., Pope, S., Cocheme, H. M., Khan, S., Asghari, S., Bhatia, K. P., et al. (2015). Loss of PLA2G6 leads to elevated mitochondrial lipid peroxidation and mitochondrial dysfunction. Brain *138*, 1801–1816.

King-Jones, K., Horner, M. A., Lam, G., and Thummel, C. S. (2006). The DHR96 nuclear receptor regulates xenobiotic responses in Drosophila. Cell Metab. *4*, 37–48.

Lee, S. B., Kim, S., Lee, J., Park, J., Lee, G., Kim, Y., Kim, J.-M., and Chung, J. (2007). ATG1, an autophagy regulator, inhibits cell growth by negatively regulating S6 kinase. EMBO Rep. *8*, 360–365.

Misra, J. R., Horner, M. A., Lam, G., and Thummel, C. S. (2011). Transcriptional regulation of xenobiotic detoxification in Drosophila. Genes Dev. *25*, 1796–1806.

Osterwalder, T., Yoon, K. S., White, B. H., and Keshishian, H. (2001). A conditional tissue-specific transgene expression system using inducible GAL4. Proc. Natl. Acad. Sci. U. S. A. *98*, 12596–12601.

Poirier, L., Shane, A., Zheng, J., and Seroude, L. (2008). Characterization of the Drosophila

gene-switch system in aging studies: a cautionary tale. Aging Cell *7*, 758–770.

Ritchie, M. E., Phipson, B., Wu, D., Hu, Y., Law, C. W., Shi, W., and Smyth, G. K. (2015). limma powers differential expression analyses for RNA-sequencing and microarray studies. Nucleic Acids Res. *43*, e47.

Rogers, I., Kerr, F., Martinez, P., Hardy, J., Lovestone, S., and Partridge, L. (2012). Ageing increases vulnerability to aβ42 toxicity in Drosophila. PLoS One *7*, e40569.

Schuster, E. F., Blanc, E., Partridge, L., and Thornton, J. M. (2007). Correcting for sequence biases in present/absent calls. Genome Biol. *8*, R125.

Slack, C., Giannakou, M. E., Foley, A., Goss, M., and Partridge, L. (2011). dFOXO-independent effects of reduced insulin-like signaling in Drosophila. Aging Cell *10*, 735–748.

Sykiotis, G. P., and Bohmann, D. (2008). Keap1/Nrf2 signaling regulates oxidative stress tolerance and lifespan in Drosophila. Dev. Cell *14*, 76–85.

Tricoire, H., Battisti, V., Trannoy, S., Lasbleiz, C., Pret, A. M., and Monnier, V. (2009). The steroid hormone receptor EcR finely modulates Drosophila lifespan during adulthood in a sex-specific manner. Mech. Ageing Dev. *130*, 547–552.

Wong, R., Piper, M. D. W., Wertheim, B., and Partridge, L. (2009). Quantification of food intake in Drosophila. PLoS One *4*, e6063.
