# Supplementary material for: Lithium Promotes Longevity through GSK3/NRF2-Dependent Hormesis
Source: Cell Rep. 2016 Apr 7;15(3):638–50. doi: 10.1016/j.celrep.2016.03.041 (PMC4850359; doi:10.1016/j.celrep.2016.03.041)
Supplement: Document S2. Article plus Supplemental Information [file mmc2.pdf]

Cell Reports

Article

Lithium Promotes Longevity through GSK3/NRF2-Dependent Hormesis

Graphical Abstract

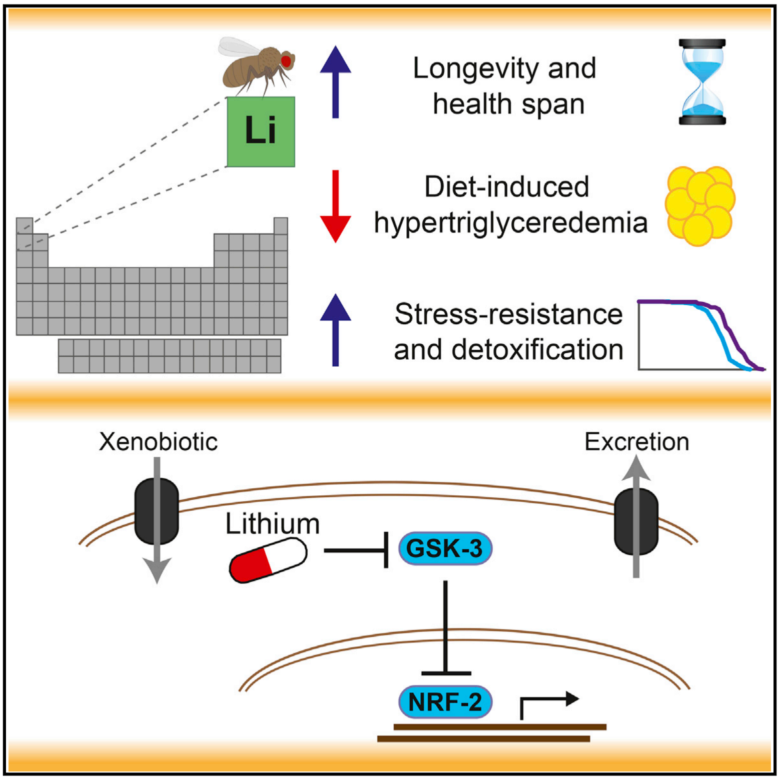

Authors

Jorge Iván Castillo-Quan, Li Li, Kerri J. Kinghorn, ..., John Hardy, Ivana Bjedov, Linda Partridge

Correspondence

l.partridge@ucl.ac.uk

In Brief

The mood stabilizer lithium has been shown to extend lifespan in organisms ranging from yeast to flies. Castillo-Quan et al. show that lithium promotes longevity through GSK-3 inhibition and subsequent NRF-2 activation, suggesting that GSK3 is a possible drug target that might affect aging.

Highlights

- Lithium extends *Drosophila* lifespan independent of sex and genetic background
- Lithium reduces triglycerides and confers stress-resistance
- Genetic or pharmacological inhibition of GSK-3 activates NRF-2
- NRF-2 activation is required for the longevity effects of lithium

Castillo-Quan et al., 2016, Cell Reports *15*, 638–650
April 19, 2016 ©2016 The Authors
http://dx.doi.org/10.1016/j.celrep.2016.03.041

CellPress

OPEN ACCESS
CellPress

Cell Reports
Article

Lithium Promotes Longevity through GSK3/NRF2-Dependent Hormesis

Jorge Iván Castillo-Quan,[1,2,3] Li Li,[1,3] Kerri J. Kinghorn,[1,3] Dobril K. Ivanov,[4] Luke S. Tain,[2] Cathy Slack,[1,2] Fiona Kerr,[1] Tobias Nespital,[2] Janet Thornton,[4] John Hardy,[3] Ivana Bjedov,[1,5] and Linda Partridge[1,2,*]
[1]Institute of Healthy Ageing and Department of Genetics, Evolution and Environment, University College London, Darwin Building, Gower Street, London WC1E 6BT, UK
[2]Max Planck Institute for Biology of Ageing, Joseph-Stelzmann Strasse 9-b, 50931 Köln, Germany
[3]Department of Molecular Neuroscience, Institute of Neurology, University College London, Queen Square, London WC1N 3BG, UK
[4]European Molecular Biology Laboratory, European Bioinformatics Institute (EMBL-EBI), Wellcome Trust Genome Campus, Hinxton, Cambridge CB10 1SD, UK
[5]UCL Cancer Institute, Paul O'Gorman Building, 72 Huntley Street, London WC1E 6BT, UK
*Correspondence: l.partridge@ucl.ac.uk
http://dx.doi.org/10.1016/j.celrep.2016.03.041

SUMMARY

The quest to extend healthspan via pharmacological means is becoming increasingly urgent, both from a health and economic perspective. Here we show that lithium, a drug approved for human use, promotes longevity and healthspan. We demonstrate that lithium extends lifespan in female and male *Drosophila*, when administered throughout adulthood or only later in life. The life-extending mechanism involves the inhibition of glycogen synthase kinase-3 (GSK-3) and activation of the transcription factor nuclear factor erythroid 2-related factor (NRF-2). Combining genetic loss of the NRF-2 repressor Kelch-like ECH-associated protein 1 (Keap1) with lithium treatment revealed that high levels of NRF-2 activation conferred stress resistance, while low levels additionally promoted longevity. The discovery of GSK-3 as a therapeutic target for aging will likely lead to more effective treatments that can modulate mammalian aging and further improve health in later life.

INTRODUCTION

Lithium is the most commonly prescribed drug for the treatment of bipolar disorder. It also improves disease phenotypes in animal models of many clinical conditions including Alzheimer disease, depression, and stroke (Chiu and Chuang, 2010). The effects of lithium on aging have been documented in yeast and *Caenorhabditis elegans*, with lithium extending lifespan (McColl et al., 2008; Zarse et al., 2011; Tam et al., 2014; Sofola-Adesakin et al., 2014). The effects of lithium on *Drosophila* aging have previously been inconclusive, with demonstration of both positive and negative effects on survival (Matsagas et al., 2009; Zhu et al., 2015). Moreover, lithium concentration in the drinking water of a large Japanese population has been associated with reduced all-cause mortality (Zarse et al., 2011), suggesting that lithium may be a bona fide anti-aging drug. However, the mechanisms by which lithium acts in humans remain poorly understood.

In vitro studies have reported that lithium can protect against several forms of oxidative and xenobiotic stressors (Lai et al., 2006; Schäfer et al., 2004), but in vivo evidence for such protective effects of lithium is lacking. Longevity has been extensively correlated with resistance to stress (Minois, 2000; Rattan, 2008; Calabrese et al., 2011; Epel and Lithgow, 2014). Transcriptomic analysis of interventions known to extend lifespan have identified particular genes likely to be involved in stress resistance (McElwee et al., 2007; Steinbaugh et al., 2012). Upregulation of the transcription factor cap'n'collar C (CncC, an NRF-2 homolog) has been shown not only to confer resistance to toxic compounds, but also to promote longevity in *C. elegans* and flies (Tullet et al., 2008; Sykiotis and Bohmann, 2008; Ewald et al., 2015). In flies and mammals, NRF-2/CncC is negatively inhibited through cytosolic sequestration and proteasomal degradation by the canonical Keap1 (Hayes and Dinkova-Kostova, 2014; Pitoniak and Bohmann, 2015). However, a second emerging upstream regulator of NRF-2/CncC is GSK-3, a well-documented target of lithium (Jope, 2003; Hayes and Dinkova-Kostova, 2014; Cuadrado, 2015; Hayes et al., 2015; Blackwell et al., 2015). GSK-3 regulates NRF-2 by phosphorylation and nuclear exclusion, an effect that is evolutionarily conserved from invertebrates to mammals (Salazar et al., 2006; An et al., 2005). Interestingly, GSK-3 inhibition has been shown to phenocopy the effects of lithium for protection against xenobiotic stress in vitro (Lai et al., 2006; Schäfer et al., 2004).

Activation of NRF-2/CncC produces hormetic effects on lifespan, such that at low level NRF-2/CncC activity extends lifespan while higher levels of activation limit it (Mattson, 2008; Maher and Yamamoto, 2010). Interestingly a hormetic signature was recently reported for the survival of a mammalian cell line treated with lithium (Suganthi et al., 2012), suggesting that lithium and GSK-3 inhibition could influence

This is an open access article under the CC BY license (http://creativecommons.org/licenses/by/4.0/).

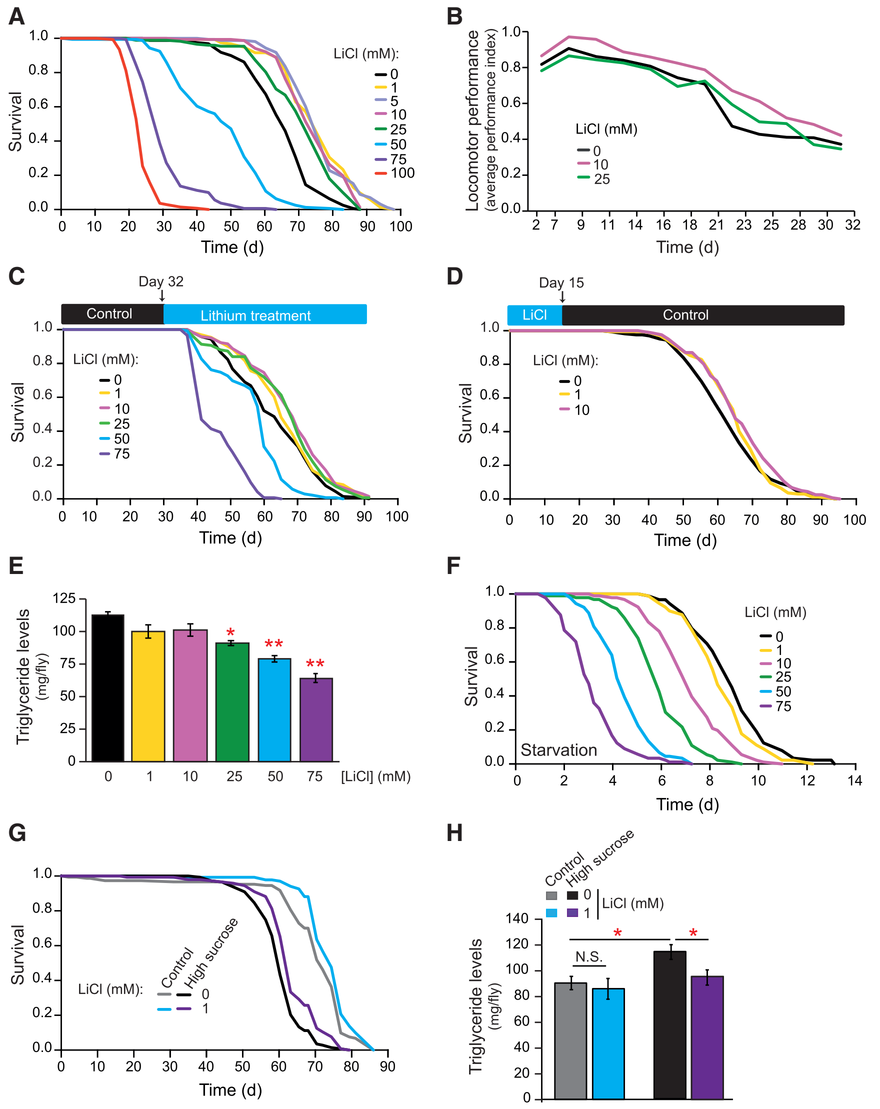

Figure 1. Lithium Regulated Longevity and Metabolism in *Drosophila*

(A) Lithium extended lifespan of w^{Dah} *Drosophila* females (n = 160 flies per condition) at concentrations between 1 and 25 mM (+16% and +18% median and maximum lifespan extension; $p < 0.001$), but resulted in a dose-dependent reduction in lifespan at concentrations between 50 and 100 mM ($p < 0.001$).

(B) Lithium treated female w^{1118} flies showed a significant improvement and protection against age-related locomotor decline ($p < 0.01$, two-way ANOVA for 10 mM).

(C) Lithium extended lifespan of aged, 32-day-old female w^{Dah} flies at concentrations from 1 to 25 mM (30 days later than in Figure 1A): 1 mM extended median lifespan by 5% (4 days) and maximum lifespan by 13% (8 days; $p < 0.05$); 10 and 25 mM lithium increased median lifespan by 9% (6 days); 10 mM increased maximum lifespan by 4.5% (3.5 days); whereas 25 mM lengthened it by 8% or 6 days ($p < 0.01$); and 50 and 75 mM significantly shortened lifespan ($p < 0.01$). n = 150 flies per condition.

(D) Brief treatment with lithium for 15 days early in adulthood extended lifespan of female w^{Dah} flies ($p < 0.05$ for 1 mM and $p < 0.01$ for 10 mM; n = 150 flies per condition).

(E) Lithium induced a dose-dependent reduction in triglyceride levels. Bars represent means of six replicas of five flies per condition ± SEM. *$p < 0.01$, **$p < 0.001$.

(F) Female w^{Dah} flies pre-treated with lithium for 15 days were subsequently sensitive to starvation in a dose-dependent manner (n = 90 flies per condition).

(G) Lithium treatment significantly extended the lifespan of w^{1118} female flies exposed to a four times higher sucrose concentration (2g/L; $p < 0.001$; n = 120 flies per condition).

(H) The increase of triglycerides observed on a high-sucrose diet was completely blocked after 15 days of treatment with 1 mM lithium. Bars represent means of six replicas of five w^{1118} female flies per condition ± SEM. *$p < 0.01$.

animal lifespan and stress resistance through activation of NRF-2.

Here we show that lithium supplementation in the diet can modulate longevity, stress resistance, and metabolism in *Drosophila* through the inhibition of GSK-3. Correspondingly, genetic downregulation of GSK-3 and lithium treatment are epistatic, suggesting a common molecular pathway. We also show that lithium and the genetic inhibition of GSK-3 promote xenobiotic stress resistance and lifespan extension through the activation of a transcriptional response mediated by CncC/NRF-2. Furthermore, lithium protects against a high-sucrose diet and acts through mechanisms that only partially overlap with those mediating lifespan extension by dietary restriction (DR). These findings demonstrate an alternative genetic and pharmacological target for the promotion of longevity and stress resistance, and emphasize the potential of pharmacological inhibitors of GSK-3 as viable anti-aging treatments.

RESULTS

Lithium Extends Healthy Lifespan in *Drosophila*

To assess the role of lithium in *Drosophila* aging, we treated adult female flies with lithium chloride (LiCl) by supplementation in their food. Lithium treatment in the range of 1 to 25 mM resulted in lifespan extension, whereas higher doses (50–100 mM) shortened lifespan (Figure 1A). These effects of lithium treatment on lifespan extension were also observed in an independent genetic background (Figure S1A) and in males (Figure S1B). Thus, lithium treatment extended *Drosophila* lifespan independently of genetic background and sex.

To ensure that the increased lifespan observed with lithium supplementation was dependent on the addition of lithium itself, we treated flies with equivalent molar concentrations of sodium chloride (NaCl) and found no lifespan extension (Figures S1C and S1D). Thus, the pro-longevity effect of LiCl is specific to lithium and not its chloride counterion.

Interestingly, we observed that, unlike with many other genetic and pharmacological interventions (e.g., DR, insulin/IGF down-regulation, rapamycin, or trametinib treatment), lithium did not reduce fecundity at life-extending doses or compromise feeding behavior (Figures S1E and S1F). Moreover, it delayed locomotor decline at two concentrations that extend lifespan (Figure 1B). Thus, lithium promotes healthspan in adult *Drosophila* with limited side effects.

Lithium Extends Lifespan in Mid-life or with Short-Term Treatment in Young Flies

To limit the side effects of long-term use, a drug that improves lifespan and healthspan will ideally do so with late-onset administration (Castillo-Quan et al., 2015; Longo et al., 2015). We therefore assessed the effect of commencing lithium treatment at older ages. Flies were switched onto food containing a range of lithium concentrations (1–75 mM) at 32 days of age (Figure 1C). Lower doses (1–25 mM) of lithium extended lifespan, whereas higher doses (50 and 75 mM) significantly reduced lifespan, similar to the dose-dependent effects we observed in younger flies.

We also tested whether transient lithium treatment early in life could increase lifespan. We therefore exposed young flies to 1 or 10 mM lithium for 15 days and then switched them to control food for the remainder of their lifespans. Early treatment with these doses of lithium extended lifespan (Figure 1D). Lithium treatment early in life, and for a transient period, can therefore increase survival later in life.

Lithium Alters Lipid Metabolism and Promotes Survival under a High-Sugar Diet

Genetic and environmental interventions that extend lifespan often induce abnormalities in carbohydrate and lipid metabolism (Barzilai et al., 2012; Wang et al., 2014; Lamming et al., 2013). We therefore examined the effects of lithium on whole body trehalose, glycogen, and triglyceride levels. Following 15 days of lithium treatment, and over a wide range of lithium concentrations, we were unable to detect a significant change in the levels of either trehalose or glycogen (Figures S1G and S1H). However, we observed a dose-dependent reduction in whole body triglycerides, the main lipid storage in flies (Ballard et al., 2008; Skorupa et al., 2008) (Figures 1E and S1I). In keeping with the lowered triglyceride levels (Ballard et al., 2008; Ulgherait et al., 2014), lithium treatment reduced survival under starvation conditions in a dose-dependent manner (Figures 1F and S1J). Moreover, lithium also extended lifespan under dietary conditions that promote triglyceride accumulation (Skorupa et al., 2008). Flies fed a high-sucrose diet were short lived and lithium was able to partially rescue this defect (Figure 1G) while completely blocking the increase in triglycerides observed with a sucrose-rich diet (Figure 1H). Therefore, lithium can extend lifespan under obesogenic dietary conditions.

Lithium and DR Extend Lifespan via Partially Overlapping Mechanisms

We next investigated whether lithium treatment was acting as a DR mimetic. DR is a well-established anti-aging intervention that extends healthy lifespan in diverse species (de Cabo et al., 2014; Fontana and Partridge, 2015), and some pharmacological and genetic interventions that extend lifespan have features of DR mimetics (Ingram and Roth, 2015; de Cabo et al., 2014). To determine whether lithium and DR extend lifespan by similar mechanisms, we assessed whether lithium could extend lifespan beyond the maximum achievable by DR. To maximize lifespan under DR, we varied the yeast concentration in the food while maintaining a constant concentration of sucrose (Bass et al., 2007), resulting in a typical tent-shaped response, with peak lifespan at food containing a 1.0 yeast concentration (Figures 2A and S2A–S2D). If lithium treatment and DR share overlapping pathways, then lithium would not be able to further extend lifespan already maximized by DR (Gems et al., 2002; Castillo-Quan et al., 2015). All lithium doses tested significantly extended median lifespan in both the yeast condition that maximized lifespan (1.0 yeast; Figures 2A and S2C) and under full feeding (2.0 yeast; Figures 2A and S2D), with greatest extension of median lifespan with 10 mM lithium under full feeding. However, under reduced yeast concentrations that shorten lifespan (0.2 and 0.5 yeast), 10 mM lithium either significantly reduced lifespan (Figure S2B) or did not confer a significant lifespan benefit (Figure S2A). Cox proportional hazards analysis showed a significant interaction between lithium and yeast concentrations for lifespan (interaction term $p < 0.0001$). The extension of lifespan from lithium increased with the level of yeast in the fly diet, suggesting partially overlapping mechanisms to those of DR.

Lithium Extends Lifespan through Inhibition of GSK-3

A well-known target of lithium is GSK-3 (Phiel and Klein, 2001; Jope, 2003; Eldar-Finkelman and Martinez, 2011). We therefore evaluated the phosphorylation status of the fly ortholog of GSK-3, Shaggy (Sgg), in response to lithium treatment. Lithium addition to the fly medium resulted in a dose-dependent increase in the inhibitory phosphorylation (Serine 9 or S9) of Sgg (Figure 2B). To evaluate the role of Sgg in lithium-mediated lifespan extension, we directly manipulated its activity in adult flies. Ubiquitous overexpression of wild-type or constitutively active Sgg (SggS9A) significantly reduced lifespan by ~30% and 50%, respectively (Figures 2C and S2E). This reduction in lifespan was almost completely reversed by lithium treatment. Furthermore, RNAi-mediated reduction in *sgg* expression using two independent dsRNA-expressing transgenes significantly increased lifespan (Figures 2D and S2F). Importantly, lithium was unable to further increase the lifespan of these *sgg* RNAi knockdown mutants flies (Figure 2D). Taken together, these findings suggest that Sgg/GSK-3 inhibition and lithium treatment increase lifespan by acting on the same downstream targets.

Lithium Activates the Cap'n'Collar C/NRF-2 Transcription Factor

To identify downstream mediators of lifespan extension by lithium and of GSK-3 inhibition, we analyzed the genome-wide transcript profiles of lithium-treated flies using microarrays. Genes encoding ribosomal proteins were among the most upregulated (Figure 3A) and downregulated (Figure S3A) gene ontology (GO) categories in lithium-treated flies. This transcriptional response could underlie the translational repression following lithium treatment that has been previously observed

OPEN ACCESS
CellPress

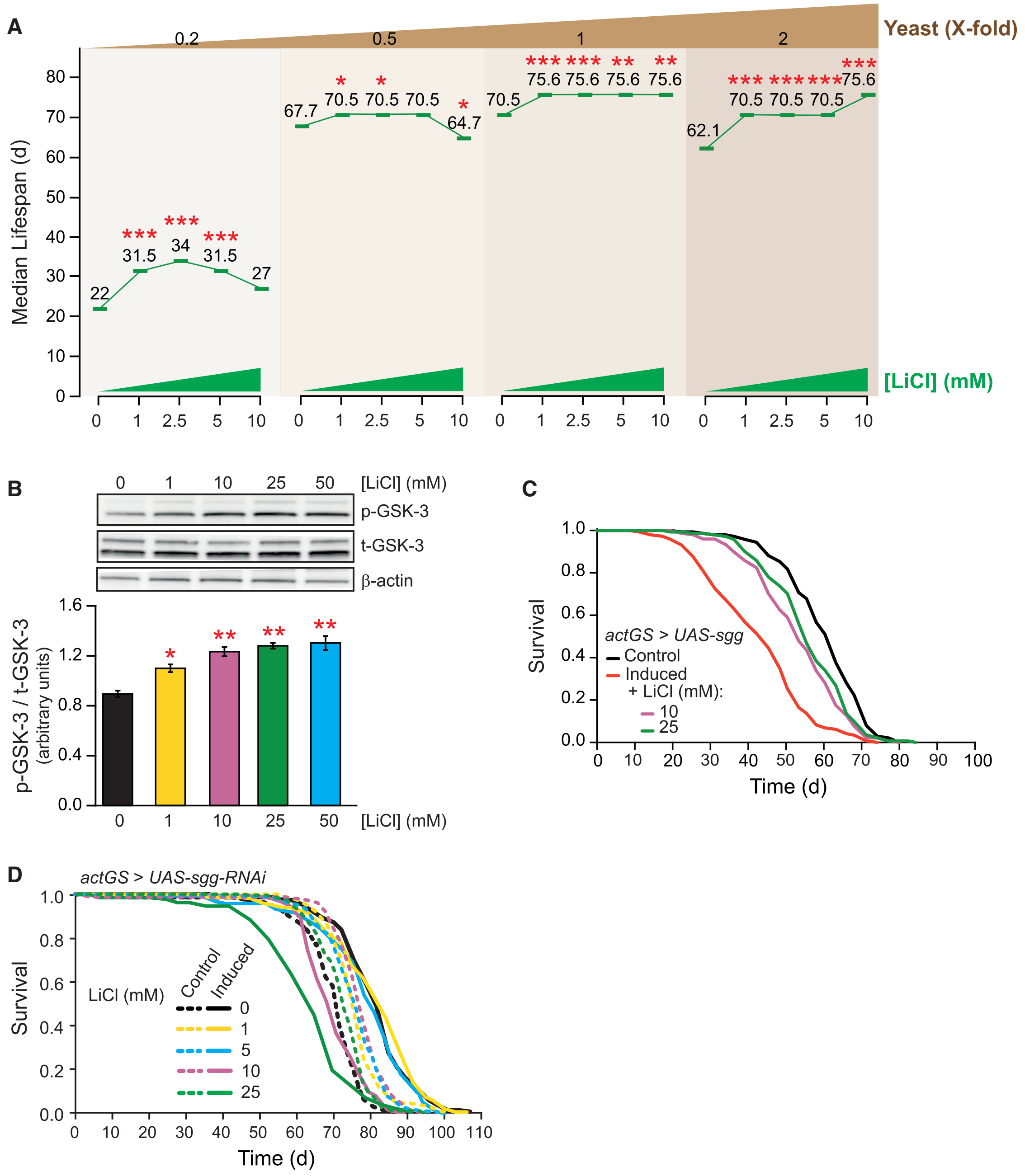

Figure 2. Lithium Extended Lifespan beyond Dietary Restriction by Inhibiting Sgg/GSK-3

(A) Median lifespans at different lithium concentrations (0, 1, 2.5, 5, or 10 mM) are plotted for four different yeast concentrations (0.2×, 0.5×, 1.0×, and 2.0× yeast): 1–5 mM lithium extended lifespan under all dietary conditions tested. Although 10 mM lithium prolonged life at 1.0× and 2.0×, it showed no effect at 0.2× and significantly shortened lifespan at 0.5× yeast. *p < 0.05, **p < 0.01, ***p < 0.001, from 0 lithium; n = 160 flies per condition. Complete survival curves are shown in Figures S2A–S2D.

(B) Lithium treatment for 15 days significantly increased the inhibitory phosphorylation of Sgg/GSK-3 in a dose-dependent manner. Bars represent means of triplicates of ten flies per biological repeat ± SEM, *p < 0.05, **p < 0.01.

(C) Ubiquitous overexpression of wild-type *sgg* significantly shortened lifespan (p < 0.001) and this was partially rescued by lithium treatment at two concentrations (10 and 25 mM; p < 0.001). See Figure S2E for the interaction of *sgg(S9A)* and lithium treatment on lifespan.

(D) Ubiquitous RNAi-mediated downregulation of *sgg* extended lifespan (p < 0.001) and no further extension occurred when the flies were treated with 1 or 5 mM lithium (p > 0.05), whereas 10 mM lithium treatment restored the lifespan to control levels (p > 0.05), and 25 mM was significantly toxic (p > 0.05). See Figure S2F for lifespan extension obtained with an independent RNAi line.

in fission yeast and *Drosophila* heads (Sofola-Adesakin et al., 2014). In addition, five GO terms for genes encoding enzymes in the detoxification pathway were also in the ten most upregulated categories (Figure 3A).

The responses to xenobiotics and oxidative stress in *Drosophila* are regulated by the transcription factors dFOXO, CncC, and DHR96 (Salih and Brunet, 2008; Sykiotis and Bohmann, 2010; Tullet, 2015; Hoffmann and Partridge, 2015; Blackwell et al., 2015). We therefore assessed whether the transcriptional responses to activation of these transcription factors overlapped with that of lithium treatment. The transcriptomic response to lithium did not overlap with that of dFOXO-dependent or -independent transcriptional regulation downstream of IIS (Figures S3B and S3C) (Alic et al., 2011). Furthermore, although we detected a significant overlap in the transcriptional signatures of lithium and DHR96 (King-Jones et al., 2006), they did not share the same directionality (Figure S4). However, we found a significant overlap (Figure 3B) between the genes that were upregulated by lithium and *cncC* overexpression (Misra et al., 2011), but not between genes downregulated by both treatments (Figure S5A), suggesting that lithium might activate a CncC transcriptional response downstream of GSK-3. The barbiturate phenobarbital activates CncC and induces a similar transcriptional response to that of *cncC* overexpression (Misra et al., 2011). We therefore analyzed the overlap between the transcriptional profiles induced by lithium and phenobarbital treatment, and again found a significant overlap (Figure 3B) between upregulated, but not downregulated, genes (Figure S5B). The genes upregulated in common between lithium treatment, phenobarbital treatment and *cncC* overexpression (Figures 3B and S5C) encoded enzymes that participate in all three phases of xenobiotic metabolism (Figure 3C). To further confirm the activation of CncC by lithium, we used a previously generated CncC reporter that responds to both chemical and genetic inducers of CncC (Sykiotis and Bohmann, 2008). Flies carrying the GstD-eGFP CncC reporter showed a dose-dependent increase in GFP expression with increasing concentrations of lithium (Figure 3D). Taken together, our results suggest that lithium activates CncC to upregulate the expression of genes in the detoxification pathway.

Lithium Induces Lifespan-Extension, Hormesis, and Protection against Xenobiotics via CncC-Dependent Mechanisms

We next assessed whether CncC activity is required for the pro-longevity effects of lithium. Ubiquitous, RNAi-mediated knockdown of *cncC* expression blocked the lifespan extension of 1 to 10 mM lithium, but was detrimental to survival in flies treated with 25 mM lithium, the highest dose that extends lifespan under basal conditions, albeit to a lesser extent (Figure 4A). Thus, lithium treatment requires CncC activity to confer its longevity benefits.

Because CncC/NRF-2 can induce hormesis (Mattson, 2008; Maher and Yamamoto, 2010), we assessed whether lithium can also do this. To test for a hormetic effect of lithium at low doses, we pre-treated flies with a range of concentrations of lithium and then challenged them with a toxic dose of 500 mM. Most pre-treatment doses of lithium induced subsequent resistance to the toxic dose (Figure 4B). To assess whether the hormetic response of lithium was mediated by CncC, we knocked down expression of *cncC* using RNAi, and treated the flies with 1–25 mM lithium. Reduction in *cncC* expression completely blocked the hormetic response induced by 10 mM lithium pre-treatment, and significantly reduced the effect of 25 mM lithium (Figure 4C).

We next assessed the ability of lithium pre-treatment to protect against other xenobiotics. Flies pre-treated with increasing concentrations of lithium ranging from 1 to 100 mM were significantly resistant to a toxic concentration of phenobarbital, with lithium doses between 1 and 75 mM almost doubling survival (Figure 4D). Lower doses of lithium also protected against a toxic dose of the anti-malarial drug, chloroquine (Figure S5D; 1–10 mM), and the pesticide paraquat (Figure 4E). Thus, low to intermediate concentrations of lithium protect against xenobiotic toxicity. To determine the role of CncC activity in lithium-mediated protection against phenobarbital, we used RNAi to knock down expression of *cncC*, which sensitized the flies to phenobarbital and completely abrogated the protection against phenobarbital afforded by lithium supplementation (Figure 4F). Thus, CncC is at least partly responsible for the hormetic effect induced by low-level treatment with lithium.

To confirm that Sgg, upstream of CncC, is also necessary for the resistance to xenobiotic stress (Blackwell et al., 2015; Cuadrado, 2015; Hayes et al., 2015), we assessed the effect of ubiquitous overexpression of wild-type *sgg* or the constitutively active Sgg(S9A) on xenobiotic resistance. Both significantly sensitized flies to phenobarbital (Figures 5A and S5E). We confirmed that *sgg* or *sgg(S9A)* overexpression regulated CncC by showing significantly lower levels of *MRP* and *keap1* (Figures 5B and S5F), both CncC target genes. Correspondingly, RNAi-mediated knockdown of *sgg* resulted in resistance to phenobarbital (Figure 5C), and paraquat (Figure S5G). An increase of mRNA levels of *cncC*, *keap1*, and *gstD2* confirmed that CncC was active in *sgg* knockdown flies (Figure 5D). Thus, increased Sgg activity sensitizes against xenobiotic stressors, whereas its inhibition protects against them.

Lifespan and Stress Resistance Depend on the Degree of Activation of CncC by Keap1 and Lithium Treatment

In addition to activating CncC by repressing Sgg/GSK-3, lithium could potentially increase CncC activity by inhibiting its canonical repressor Keap1 (Cuadrado, 2015; Pitoniak and Bohmann, 2015). Hence, we analyzed the interaction between lithium treatment and Keap1. Overexpression of Keap1, which inhibits CncC activity in vivo (Sykiotis and Bohmann, 2008), was unable to prevent the lifespan-extending properties of lithium (Figure 6A), suggesting that the longevity effect of lithium treatment is independent of Keap1. Next, we analyzed the interaction of loss of Keap1 and lithium treatment. We generated a deletion of the *keap1* coding sequence by P-element-mediated male recombination using a previously described P-element insertion line (Sykiotis and Bohmann, 2008) (Figure 6B). The *keap1* deletion (*keap1*^{Del}) was homozygous lethal, but activated CncC 4-fold in the heterozygous state, as measured by the CncC reporter (Figure 6C). Lithium treatment of the *keap1*^{Del} flies further activated CncC (Figure 6C). We next tested whether this effect on CncC

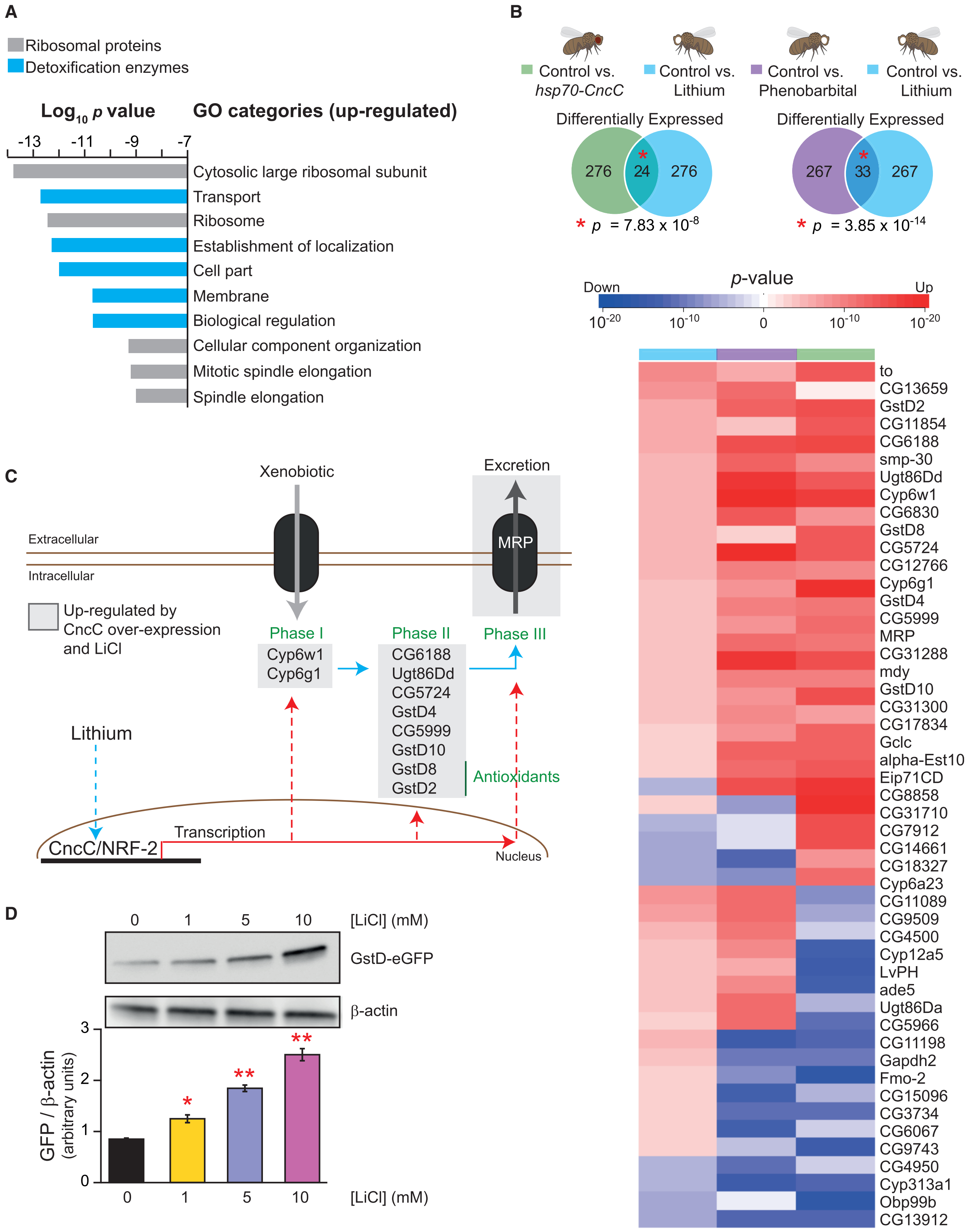

(legend on next page)

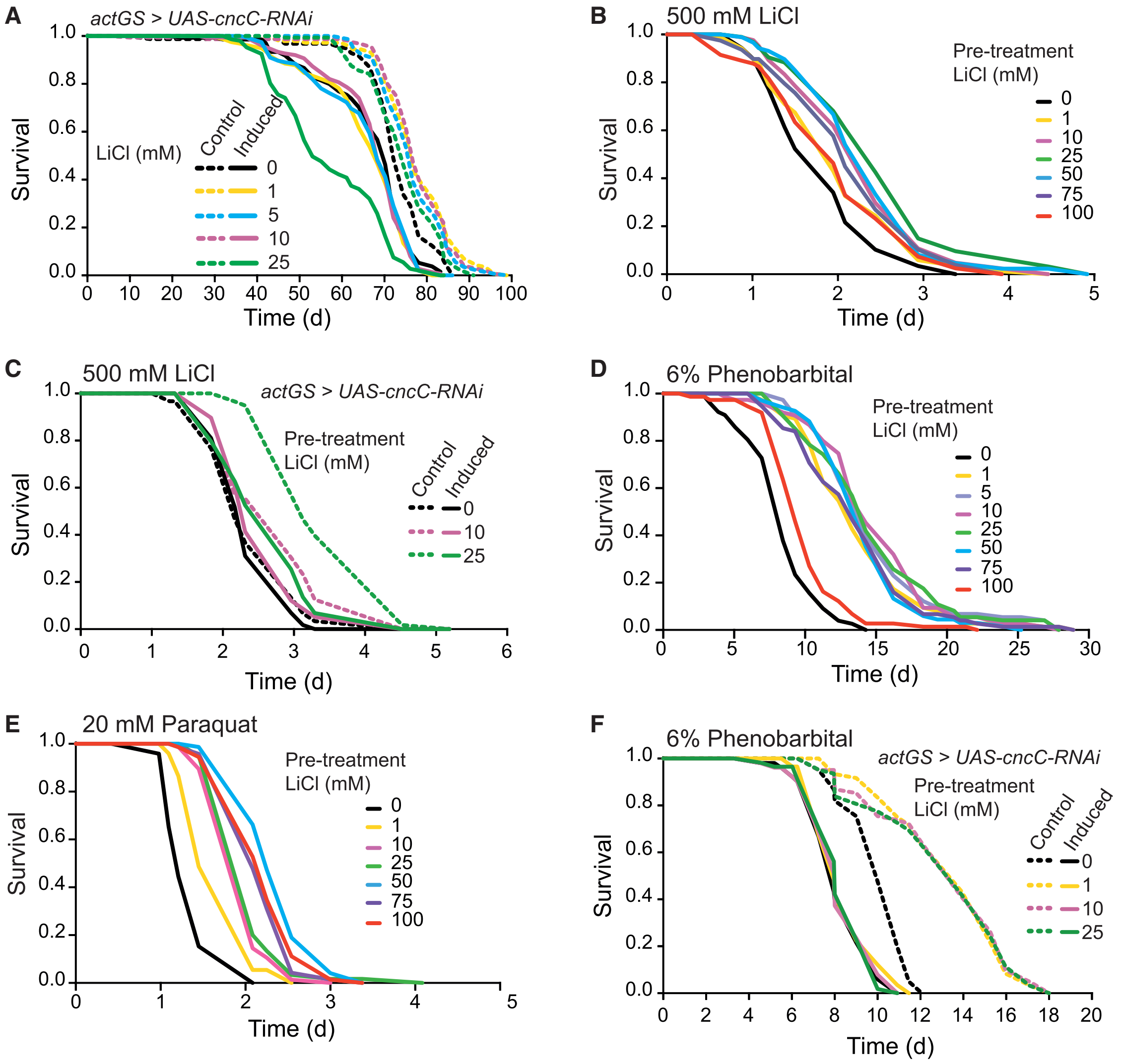

Figure 4. Lithium-Induced Xenobiotic Resistance and Longevity Were Mediated by CncC

(A) Ubiquitous knockdown of *cncC* blocked lifespan extension by lithium.

(B) Pre-treatment with increasing concentrations of lithium protected against a subsequent toxic dose of lithium (500 mM; $p < 0.01$ for doses from 10 to 100 mM; $p < 0.05$ for 1 mM).

(C) Ubiquitous downregulation of *cncC* blocked the protective effect of 10 mM lithium pre-treatment against a subsequent toxic dose and partially blocked the protective effect of 25 mM lithium pre-treatment.

(D) 1 to 100 mM lithium pre-treatment protected against a 6% phenobarbital ($p < 0.001$ for all doses).

(E) Lithium pre-treatment (for 15 days) protected against the herbicide paraquat in a dose-dependent manner ($p < 0.001$ for all doses, with maximal protection at 50 mM).

(F) RNAi-mediated downregulation of *cncC* completely blocked the protective effect of lithium against phenobarbital.

activation protected against paraquat and lithium toxicity. *keap1^{Del}* flies were significantly resistant to both paraquat and lithium (Figures 6D and 6E), and pre-treatment with lithium further protected them. We confirmed these findings using a previously described heterozygous loss-of-function mutation in the *keap1* gene (*keap1^{EY5}*) (Sykiotis and Bohmann, 2008) (Figures

Figure 3. Lithium Activated a Transcriptional Response Similar to that of CncC/NRF-2

(A) Ten most significantly upregulated GO categories induced by lithium treatment of w^{1118} female flies. See Figure S3A for downregulated GO categories.

(B) Lithium treatment of w^{1118} females flies induced a transcriptional response that significantly overlapped with that induced by *cncC* overexpression ($p = 7.83 \times 10^{-8}$) or phenobarbital treatment ($p = 3.85 \times 10^{-14}$) (Misra et al., 2011). Heatmap showing the 57 genes most significantly changed by lithium or phenobarbital treatment and overexpression of *cncC*.

(C) Genes upregulated by lithium treatment mapped to the three phases of the xenobiotic detoxification pathway in flies.

(D) Lithium treatment of w^{Dah} female flies upregulated Gst-D protein levels. Bars represent means of triplicates of ten flies per condition ± SEM. *$p < 0.05$, **$p < 0.01$.

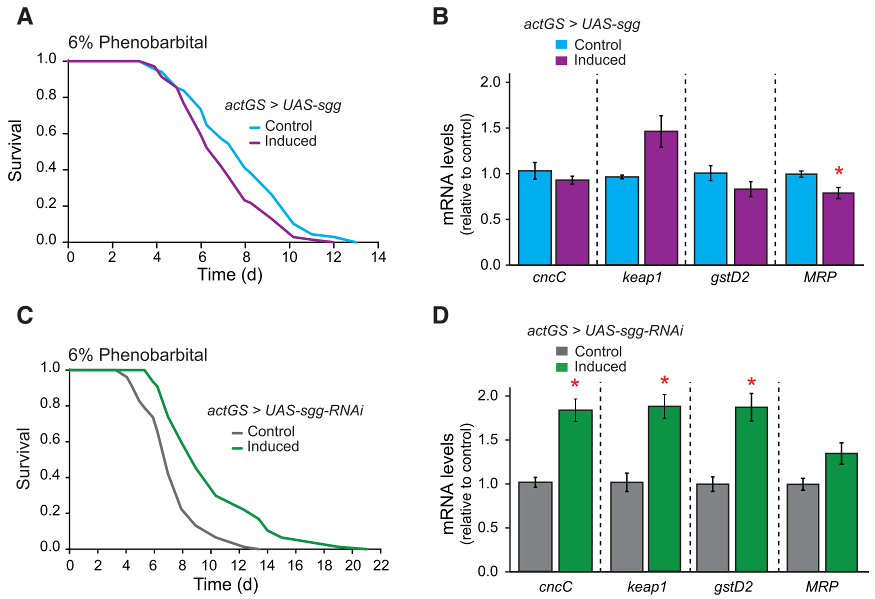

Figure 5. Reduced Activity of GSK-3 Increased Resistance to Xenobiotics

(A) Ubiquitous overexpression of wild-type *sgg* significantly ($p < 0.05$) reduced survival under xenobiotic stress with phenobarbital. n = 75 flies per condition.

(B) Overexpression of wild-type *sgg* significantly reduced multidrug-resistance like protein 1 (*MRP*) mRNA levels ($p < 0.05$, paired t test), whereas non-significant trends were detected for glutathione S transferase D2 (*gstD2*) and *cncC* mRNA levels ($p > 0.05$). A non-significant increase of *keap1* mRNA levels was observed.

(C) RNAi-mediated knockdown of *sgg* protected against phenobarbital stress ($p < 0.001$). n = 75 flies per condition.

(D) Knockdown of *sgg* increased mRNA levels of *cncC*, *keap1* and *gstD2* ($p < 0.05$), while a non-significant increase was observed for *MRP* mRNA levels.

S6A and S6B). Thus, the combination of loss of *keap1* and lithium treatment further protected against paraquat and lithium-induced toxicity, suggesting that stronger CncC activation results in greater protection against these xenobiotics.

We subsequently evaluated the interaction between loss of *keap1* and lithium treatment for longevity. Survival analysis showed that the lifespan of *keap1*^{Del} mutant flies was indistinguishable from controls, but that addition of 1 mM lithium marginally, yet significantly, extended lifespan (Figure 6F). Increasing the dose of lithium to 10 mM restored longevity to control levels. The *keap1*^{EY5} mutant flies showed a significant lifespan extension (Figure 6G). However, supplementation of either 1 or 10 mM lithium to the *keap1*^{EY5} mutant shortened lifespan in a dose-dependent manner. These results suggest that the level of activation of CncC that maximizes extension of lifespan is considerably lower than that which maximizes protection against toxic doses of lithium and paraquat.

Lithium Does Not Induce or Require Autophagy to Promote Longevity

Activation of autophagy has been proposed as a mechanism for the beneficial effects of lithium (Sarkar et al., 2005). We therefore analyzed the induction of autophagy by LC3-I/LC3-II (Atg8 in *Drosophila*) levels without detecting statistically significant changes. Indeed, there was a tendency for lower LC3-I that did not reach statistical significance (Figure S7A). Moreover, lithium treatment was able to extend the lifespan of flies with autophagy defects due to heterozygous loss of *atg1* (Figure S7B) (Lee et al., 2007). Thus, taken together our results do not immediately support a role for autophagy in the pro-longevity effects of lithium treatment, and strengthen our conclusion that they are mediated through the inhibition of GSK-3 and the subsequent activation of CncC/NRF-2 (Figure 7). However, it remains possible that induction of autophagy occurs in *atg1*-deficient flies, or that lithium induces autophagy in a tissue-specific manner.

DISCUSSION

Lithium Acts as a Pro-longevity Drug

Drug repurposing is the most promising approach for developing pharmacological agents to improve healthy aging. So far, two medically approved drugs, metformin and rapamycin, have been reported to promote longevity and provide health benefits across species from invertebrates to mammals (de Cabo et al., 2014; Madeo et al., 2014; Riera and Dillin, 2015). We and others have shown that lithium can extend lifespan in fission yeast, *C. elegans*, and *Drosophila* (McColl et al., 2008; Matsagas et al., 2009; Sofola-Adesakin et al., 2014). We also showed that this effect was common between two different laboratory strains and, unlike other interventions that seem to be more effective in females (Austad and Bartke, 2015), lithium similarly extended lifespan in both sexes.

Lifespan-extending drugs can often act like DR mimetics (Madeo et al., 2014; Ingram and Roth, 2015); hence, it was important to determine whether lithium was acting in a similar manner. While low doses of lithium were able to extend lifespan at all dietary levels tested, median lifespan extension was greatest under full feeding conditions. Our data thus suggest that lithium and DR act via partially overlapping mechanisms and confirms the observation made in *C. elegans* that lithium extends lifespan of *eat-2* mutants (McColl et al., 2008), a genetic model of DR in worms. Lithium also extended the lifespan of flies fed a diet enriched with sucrose, possibly by modulating lipid metabolism (Sykiotis et al., 2011; Pang et al., 2014; Karim et al., 2015; Steinbaugh et al., 2015). However, the role of CncC in modulating the triglyceride phenotype of lithium remains to be explored. Overall, our observations strongly suggest that lithium is a pro-longevity drug capable of extending lifespan at low doses independent of sex and genetic background, and under a variety of dietary conditions.

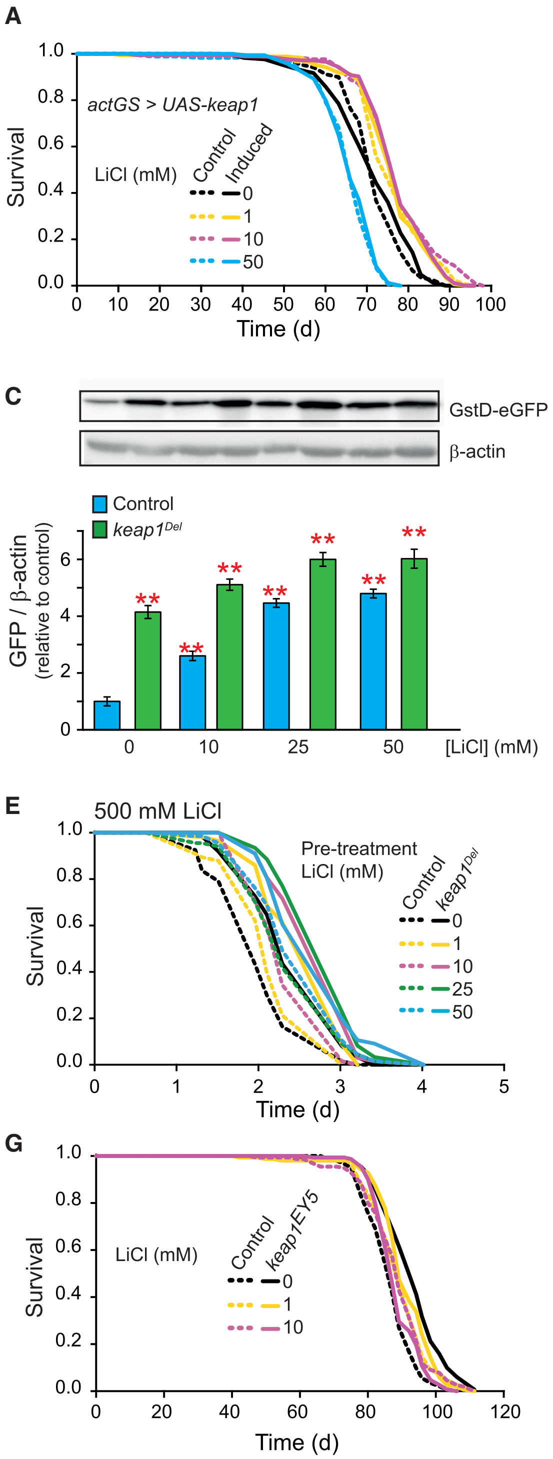

Figure 6. Higher Activation Levels of CncC Promote Xenobiotic Resistance but Not Lifespan

(A) Overexpression of *keap1* did not prevent the lifespan-modulatory effects of lithium treatment. n = 150 flies per condition.

(B) Schematic of the *keap1* gene showing the portion deleted in the *keap1^{Del}* mutant (top) and agarose gel showing start and end of P-element disrupting *keap1* coding sequence in the *keap1^{Del}* mutant (bottom).

(C) Combination of heterozygous deletion of *keap1* and lithium treatment showed a greater activation of CncC than on their own. Bars represent means of four replicas of five flies per repeat ± SEM. **$p < 0.01$.

(legend continued on next page)

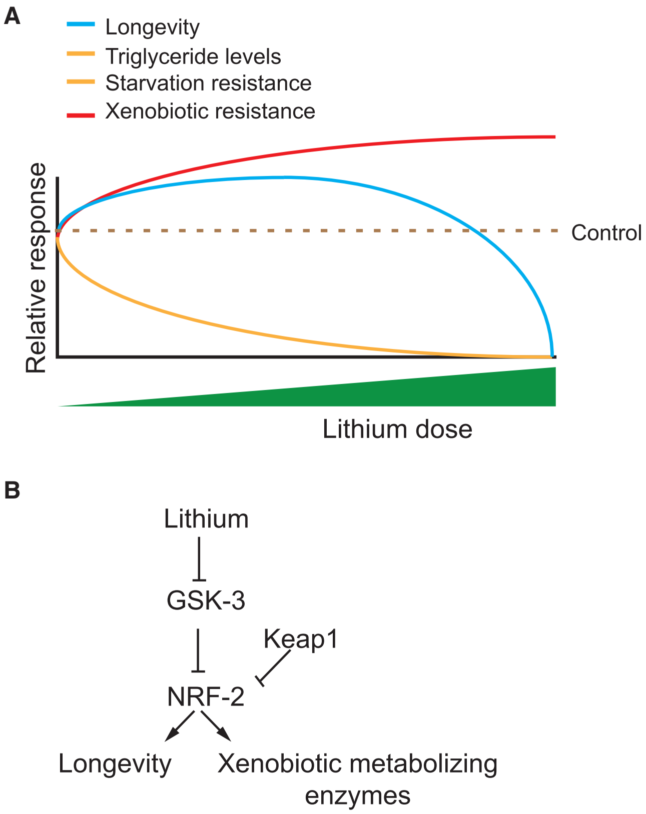

Figure 7. Lithium Regulates Longevity, Metabolism, and Stress Resistance by Inhibiting GSK-3 and Activating NRF-2

(A) Summary of findings with lithium for longevity, stress resistance, starvation, and triglyceride levels.

(B) Proposed model showing the mechanism by which lithium, Sgg/GSK-3, and CncC/NRF-2 act in the same pathway to modulate longevity and xenobiotic resistance.

Lithium Toxicity, Hormesis, and Stress Resistance

In humans, the therapeutic window for lithium treatment of bipolar disorder lies between 0.5 and 1 mM in serum, whereas concentrations of 1.5 mM and above severely increase the risk of tissue damage (Malhi and Tanious, 2011). Previous work in *Drosophila* suggests that the dose range at which we observed lifespan extension (0.5–25 mM) translates to *Drosophila* tissue concentrations below 0.5 mM (Dokucu et al., 2005). As previously reported for *C. elegans* and *Drosophila* (McColl et al., 2008; Zhu et al., 2015), concentrations above 50 mM were highly toxic.

Drug interventions to promote healthy lifespan are less likely to have side effects if started late in life (Castillo-Quan et al., 2015). Only a handful of drugs approved by the US Food and Drug Administration , namely rapamycin, metformin, and the Ras inhibitor trametinib, induce lifespan extension when commenced at later ages in model organisms (Harrison et al., 2009; Cabreiro et al., 2013; Martin-Montalvo et al., 2013; Slack et al., 2015). We found that lithium extends lifespan when first administered in mid-late life. In humans, long-term treatment with lithium for psychiatric disorders is associated with progressive and permanent renal damage (Malhi and Tanious, 2011). We showed that short treatment periods in *Drosophila*, 15 days during early adulthood, are sufficient to prolong life. Taken together, our data suggest that when testing lithium as a pro-longevity drug in mammals, lower doses than those used in psychiatric disorders are likely to be sufficient, and other strategies such as alternate-day dosing or transient treatment periods (either early or late in life), may be sufficient to reduce undesirable side effects and maximize the potential health benefits.

Interestingly, doses of lithium that shortened lifespan were protective against certain forms of xenobiotic stress. In vitro studies in mammalian cells have shown that lithium, and other GSK-3 inhibitors, protect against cell death caused by rotenone-induced oxidative stress (Lai et al., 2006), glutamate excitotoxicity, and H_2O_2 (Schäfer et al., 2004). This is likely mediated through a hormetic response (Suganthi et al., 2012), in this case orchestrated by NRF-2 activation. We observed that while simultaneous activation of CncC by loss of Keap1 and lithium treatment is additive and confers greater stress resistance to xenobiotics, the threshold for lifespan extension is perhaps considerably lower. A similar situation has been observed in *C. elegans* in which strong activation of the endoplasmic reticulum unfolded protein response conferred stress resistance benefits, while shortening lifespan (Taylor and Dillin, 2013). Our findings thus suggest that while NRF-2 activation either by loss of Keap1 or inhibition of GSK-3 is beneficial for longevity and stress resistance, at low levels of activation, stronger induction is detrimental for lifespan. This suggests that the hormetic benefits of lithium are more likely to occur at low levels under basal non-stress conditions (Calabrese, 2013). Hence, when testing for GSK-3 inhibitors or NRF-2 activators in modulating animal (and especially mammalian) aging, the degree of NRF-2 activation within the hormetic curve will determine positive or negative longevity outcomes. Future work studying the convergence of the salutary and damaging effects of lithium will aid in understanding to what extent the molecular mechanisms are shared (Calabrese and Mattson, 2011; Calabrese et al., 2013; Epel and Lithgow, 2014). Additionally, our microarray analysis was performed in heads and thoraces; therefore, it remains to be explored to what extent systemic or localized activation of NRF-2 modulates longevity, stress resistance, and lipid metabolism at the tissue level (Douglas et al., 2015).

(D) Deletion of *keap1* in flies treated with lithium showed greater protection against paraquat than either treatment on its own, with maximal effects observed at 25 mM ($p < 0.001$).

(E) The *keap1* deletion protected against toxic concentrations of lithium (500 mM), and this protection was augmented with lithium pre-treatment ($p < 0.01$).

(F) Deletion of *keap1* did not extend lifespan: 1 mM lithium ($p < 0.05$), but not 10 mM ($p > 0.05$), treatment of *keap1* flies resulted in a small but significant extension. n = 150 flies per condition.

(G) $keap1^{EY5}$ mutant flies showed significant lifespan extension ($p < 0.001$), that was dose-dependently abolished ($p > 0.05$) by lithium, likely as a result of over-activation of CncC. n = 150 flies per condition.

GSK-3 and NRF-2 as Drug Targets for Aging

Complete absence of GSK-3 in *C. elegans*, *Drosophila*, and mice shortens lifespan or prevents development (Hoeflich et al., 2000; McColl et al., 2008; Bourouis, 2002), while moderate inhibition has been associated with most of its positive effects (Avrahami et al., 2013). GSK-3 is upregulated in many disease states, including neurodegeneration, diabetes, inflammatory conditions, and some cancers (Takahashi-Yanaga, 2013). We have shown that adult-specific genetic manipulation of the fly ortholog of GSK-3, Sgg, affects longevity. Downregulation of Sgg prolonged lifespan and lithium was unable to further extend the lifespan, suggesting that lithium and inhibition of Sgg act through a common molecular pathway to extend lifespan.

In *C. elegans* and mammalian cells, GSK-3 directly interacts with NRF-2 to repress its activity, independently of Keap1 (An et al., 2005; Salazar et al., 2006; Rojo et al., 2008; Rada et al., 2012). Therefore, we hypothesized that lithium might act via Sgg/GSK-3, to de-repress CncC, the fly ortholog of NRF-2 and activate the oxidative and xenobiotic stress transcriptional signature (An et al., 2005; Hayes et al., 2015), which in turn would induce a CncC/NRF-2-dependent protective response (Jones et al., 2015; Blackwell et al., 2015). GO enrichment analysis identified a transcriptional signature that indeed suggested that lithium acts via CncC/NRF-2. CncC activity was indispensable for the lifespan extension conferred by lithium. In keeping with our results, work in rodents and mammalian cell lines has shown that lithium treatment and GSK-3 inhibition activate NRF-2 (Lee et al., 2014; Rizak et al., 2014). Because activation of CncC/NRF-2 modulates longevity in *C. elegans* and *Drosophila* (Tullet et al., 2008; Sykiotis and Bohmann, 2008; Ewald et al., 2015), our results provide evidence that GSK-3 is a viable therapeutic target to promote longevity via activation of NRF-2.

To date, the only GSK-3 inhibitor approved for human use is lithium (Williams and Harwood, 2000; Meijer et al., 2004; Martinez et al., 2011). However, researchers and pharmaceutical companies have developed more selective GSK-3 inhibitors, some of which have already entered the early stages of clinical trials for obesity, Alzheimer disease, and progressive supranuclear palsy (Eldar-Finkelman and Martinez, 2011). Our results call for a reassessment of the potential use of GSK-3 inhibitors and NRF-2 activators as potential anti-aging compounds.

EXPERIMENTAL PROCEDURES

Fly Stocks and Husbandry

The w^{1118} stock was obtained from Bloomington *Drosophila* Stock Center. The control white *Dahomey* (w^{Dah}) stock has been maintained in large population cages with overlapping generations since 1970. The w^{Dah} stock was initially derived by incorporation of the w^{1118} mutation into the outbred *Dahomey* background by backcrossing (Bass et al., 2007). Further details concerning fly mutants can be found in the Supplemental Experimental Procedures.

Lithium Treatment

LiCl (Sigma) or NaCl (Sigma) were dissolved in ddH_2O at a concentration of 5 M before supplementing to the medium. Equivalent volumes of vehicle were supplemented to the medium to compensate for dilution.

Dietary Restriction Protocol

The DR protocol was performed as described previously (Bass et al., 2007).

Statistical Analyses

Statistical analyses were performed using Excel, GraphPad Prism, or JMP software version 9 (SAS Institute). Survival experiments were analyzed using log rank test. Other data were tested by one-way analyses of variance (ANOVA) and planned comparisons of means were made using Tukey-Kramer HSD test. Cox proportional hazards analysis was performed to compare interactions for survival.

ACCESSION NUMBERS

The accession number reported for the microarray data in this paper is ArrayExpress: E-MTAB-3809.

SUPPLEMENTAL INFORMATION

Supplemental Information includes Supplemental Experimental Procedures and seven figures and can be found with this article online at http://dx.doi.org/10.1016/j.celrep.2016.03.041.

AUTHOR CONTRIBUTIONS

J.I.C.-Q. and I.B. conceived the experiments. J.I.C.-Q., I.B., L.L., K.J.K., L.S.T., T.N., and F.K. performed the experiments. D.K.I. analyzed the microarray data. C.S. and I.B. contributed reagents. J.I.C.-Q., I.B., J.T., J.H., and L.P. supervised experiments/project. J.I.C.-Q. and L.P. wrote the manuscript. All authors approved the final submission.

ACKNOWLEDGMENTS

We thank Profs. D. Gems, M. Murphy, and D. Rubinsztein, and Drs. H. Cochemé, F. Cabreiro, S. Emran, Y. de la Guardia, M. Piper, N. Woodling, A. Gilliat, and O. Sofola-Adesakin for insightful advice and comments; Dr. N. Alic for advice and assistance with statistical testing; and Dr. D. Wieser for initial analysis of microarray data. We thank the Max Planck-Genome-centre Cologne (http://mpgc.mpipz.mpg.de/home/) for microarray analysis. We acknowledge funding from UCL Scholarships (to J.I.C.-Q.), the Max Planck Society (to J.I.C.-Q., L.S.T., C.S., T.N., and L.P.), European Research Council (to I.B. and L.P.), Research Into Ageing (to I.B. and L.P.), Wellcome Trust (to D.K.I., K.J.K., J.T., J.H., and L.P.), Parkinson's UK (to L.L. and L.P.), and Alzheimer's Research UK (to F.K. and L.P.).

Received: November 9, 2015
Revised: January 31, 2016
Accepted: March 10, 2016
Published: April 7, 2016

REFERENCES

Alic, N., Andrews, T.D., Giannakou, M.E., Papatheodorou, I., Slack, C., Hoddinott, M.P., Cochemé, H.M., Schuster, E.F., Thornton, J.M., and Partridge, L. (2011). Genome-wide dFOXO targets and topology of the transcriptomic response to stress and insulin signalling. Mol. Syst. Biol. *7*, 502.

An, J.H., Vranas, K., Lucke, M., Inoue, H., Hisamoto, N., Matsumoto, K., and Blackwell, T.K. (2005). Regulation of the Caenorhabditis elegans oxidative stress defense protein SKN-1 by glycogen synthase kinase-3. Proc. Natl. Acad. Sci. USA *102*, 16275–16280.

Austad, S.N., and Bartke, A. (2015). Sex Differences in Longevity and in Responses to Anti-Aging Interventions: A Mini-Review. Gerontology *62*, 40–46.

Avrahami, L., Licht-Murava, A., Eisenstein, M., and Eldar-Finkelman, H. (2013). GSK-3 inhibition: achieving moderate efficacy with high selectivity. Biochim. Biophys. Acta *1834*, 1410–1414.

Ballard, J.W.O., Melvin, R.G., and Simpson, S.J. (2008). Starvation resistance is positively correlated with body lipid proportion in five wild caught Drosophila simulans populations. J. Insect Physiol. *54*, 1371–1376.

Barzilai, N., Huffman, D.M., Muzumdar, R.H., and Bartke, A. (2012). The critical role of metabolic pathways in aging. Diabetes *61*, 1315–1322.

Bass, T.M., Grandison, R.C., Wong, R., Martinez, P., Partridge, L., and Piper, M.D.W. (2007). Optimization of dietary restriction protocols in Drosophila. J. Gerontol. A Biol. Sci. Med. Sci. *62*, 1071–1081.

Blackwell, T.K., Steinbaugh, M.J., Hourihan, J.M., Ewald, C.Y., and Isik, M. (2015). SKN-1/Nrf, stress responses, and aging in Caenorhabditis elegans. Free Radic. Biol. Med. *88* (*Pt B*), 290–301.

Bourouis, M. (2002). Targeted increase in shaggy activity levels blocks wingless signaling. Genesis *34*, 99–102.

Cabreiro, F., Au, C., Leung, K.-Y., Vergara-Irigaray, N., Cochemé, H.M., Noori, T., Weinkove, D., Schuster, E., Greene, N.D.E., and Gems, D. (2013). Metformin retards aging in C. elegans by altering microbial folate and methionine metabolism. Cell *153*, 228–239.

Calabrese, E.J. (2013). Hormesis: Toxicological foundations and role in aging research. Exp. Gerontol. *48*, 99–102.

Calabrese, E.J., and Mattson, M.P. (2011). Hormesis provides a generalized quantitative estimate of biological plasticity. J. Cell Commun. Signal. *5*, 25–38.

Calabrese, V., Cornelius, C., Cuzzocrea, S., Iavicoli, I., Rizzarelli, E., and Calabrese, E.J. (2011). Hormesis, cellular stress response and vitagenes as critical determinants in aging and longevity. Mol. Aspects Med. *32*, 279–304.

Calabrese, E.J., Iavicoli, I., and Calabrese, V. (2013). Hormesis: its impact on medicine and health. Hum. Exp. Toxicol. *32*, 120–152.

Castillo-Quan, J.I., Kinghorn, K.J., and Bjedov, I. (2015). Genetics and pharmacology of longevity: the road to therapeutics for healthy aging. Adv. Genet. *90*, 1–101.

Chiu, C.-T., and Chuang, D.-M. (2010). Molecular actions and therapeutic potential of lithium in preclinical and clinical studies of CNS disorders. Pharmacol. Ther. *128*, 281–304.

Cuadrado, A. (2015). Structural and functional characterization of Nrf2 degradation by glycogen synthase kinase 3/β-TrCP. Free Radic. Biol. Med. *88* (*Pt B*), 147–157.

de Cabo, R., Carmona-Gutierrez, D., Bernier, M., Hall, M.N., and Madeo, F. (2014). The search for antiaging interventions: from elixirs to fasting regimens. Cell *157*, 1515–1526.

Dokucu, M.E., Yu, L., and Taghert, P.H. (2005). Lithium- and valproate-induced alterations in circadian locomotor behavior in Drosophila. Neuropsychopharmacology *30*, 2216–2224.

Douglas, P.M., Baird, N.A., Simic, M.S., Uhlein, S., McCormick, M.A., Wolff, S.C., Kennedy, B.K., and Dillin, A. (2015). Heterotypic signals from neural HSF-1 separate thermotolerance from longevity. Cell Rep. *12*, 1196–1204.

Eldar-Finkelman, H., and Martinez, A. (2011). GSK-3 inhibitors: preclinical and clinical focus on CNS. Front. Mol. Neurosci. *4*, 32.

Epel, E.S., and Lithgow, G.J. (2014). Stress biology and aging mechanisms: toward understanding the deep connection between adaptation to stress and longevity. J. Gerontol. A Biol. Sci. Med. Sci. *69* (*Suppl 1*), S10–S16.

Ewald, C.Y., Landis, J.N., Porter Abate, J., Murphy, C.T., and Blackwell, T.K. (2015). Dauer-independent insulin/IGF-1-signalling implicates collagen remodelling in longevity. Nature *519*, 97–101.

Fontana, L., and Partridge, L. (2015). Promoting health and longevity through diet: from model organisms to humans. Cell *161*, 106–118.

Gems, D., Pletcher, S., and Partridge, L. (2002). Interpreting interactions between treatments that slow aging. Aging Cell *1*, 1–9.

Harrison, D.E., Strong, R., Sharp, Z.D., Nelson, J.F., Astle, C.M., Flurkey, K., Nadon, N.L., Wilkinson, J.E., Frenkel, K., Carter, C.S., et al. (2009). Rapamycin fed late in life extends lifespan in genetically heterogeneous mice. Nature *460*, 392–395.

Hayes, J.D., and Dinkova-Kostova, A.T. (2014). The Nrf2 regulatory network provides an interface between redox and intermediary metabolism. Trends Biochem. Sci. *39*, 199–218.

Hayes, J.D., Chowdhry, S., Dinkova-Kostova, A.T., and Sutherland, C. (2015). Dual regulation of transcription factor Nrf2 by Keap1 and by the combined actions of β-TrCP and GSK-3. Biochem. Soc. Trans. *43*, 611–620.

Hoeflich, K.P., Luo, J., Rubie, E.A., Tsao, M.S., Jin, O., and Woodgett, J.R. (2000). Requirement for glycogen synthase kinase-3beta in cell survival and NF-kappaB activation. Nature *406*, 86–90.

Hoffmann, J.M., and Partridge, L. (2015). Nuclear hormone receptors: roles of xenobiotic detoxification and sterol homeostasis in healthy aging. Crit. Rev. Biochem. Mol. Biol. *50*, 380–392.

Ingram, D.K., and Roth, G.S. (2015). Calorie restriction mimetics: can you have your cake and eat it, too? Ageing Res. Rev. *20*, 46–62.

Jones, R.M., Desai, C., Darby, T.M., Luo, L., Wolfarth, A.A., Scharer, C.D., Ardita, C.S., Reedy, A.R., Keebaugh, E.S., and Neish, A.S. (2015). Lactobacilli modulate epithelial cytoprotection through the Nrf2 pathway. Cell Rep. *12*, 1217–1225.

Jope, R.S. (2003). Lithium and GSK-3: one inhibitor, two inhibitory actions, multiple outcomes. Trends Pharmacol. Sci. *24*, 441–443.

Karim, M.R., Taniguchi, H., and Kobayashi, A. (2015). Constitutive activation of Drosophila CncC transcription factor reduces lipid formation in the fat body. Biochem. Biophys. Res. Commun. *463*, 693–698.

King-Jones, K., Horner, M.A., Lam, G., and Thummel, C.S. (2006). The DHR96 nuclear receptor regulates xenobiotic responses in Drosophila. Cell Metab. *4*, 37–48.

Lai, J.S., Zhao, C., Warsh, J.J., and Li, P.P. (2006). Cytoprotection by lithium and valproate varies between cell types and cellular stresses. Eur. J. Pharmacol. *539*, 18–26.

Lamming, D.W., Ye, L., Sabatini, D.M., and Baur, J.A. (2013). Rapalogs and mTOR inhibitors as anti-aging therapeutics. J. Clin. Invest. *123*, 980–989.

Lee, S.B., Kim, S., Lee, J., Park, J., Lee, G., Kim, Y., Kim, J.-M., and Chung, J. (2007). ATG1, an autophagy regulator, inhibits cell growth by negatively regulating S6 kinase. EMBO Rep. *8*, 360–365.

Lee, T.-M., Lin, S.-Z., and Chang, N.-C. (2014). Antiarrhythmic effect of lithium in rats after myocardial infarction by activation of Nrf2/HO-1 signaling. Free Radic. Biol. Med. *77*, 71–81.

Longo, V.D., Antebi, A., Bartke, A., Barzilai, N., Brown-Borg, H.M., Caruso, C., Curiel, T.J., de Cabo, R., Franceschi, C., Gems, D., et al. (2015). Interventions to slow aging in humans: are we ready? Aging Cell *14*, 497–510.

Madeo, F., Pietrocola, F., Eisenberg, T., and Kroemer, G. (2014). Caloric restriction mimetics: towards a molecular definition. Nat. Rev. Drug Discov. *13*, 727–740.

Maher, J., and Yamamoto, M. (2010). The rise of antioxidant signaling–the evolution and hormetic actions of Nrf2. Toxicol. Appl. Pharmacol. *244*, 4–15.

Malhi, G.S., and Tanious, M. (2011). Optimal frequency of lithium administration in the treatment of bipolar disorder: clinical and dosing considerations. CNS Drugs *25*, 289–298.

Martin-Montalvo, A., Mercken, E.M., Mitchell, S.J., Palacios, H.H., Mote, P.L., Scheibye-Knudsen, M., Gomes, A.P., Ward, T.M., Minor, R.K., Blouin, M.-J., et al. (2013). Metformin improves healthspan and lifespan in mice. Nat. Commun. *4*, 2192.

Martinez, A., Gil, C., and Perez, D.I. (2011). Glycogen synthase kinase 3 inhibitors in the next horizon for Alzheimer's disease treatment. Int. J. Alzheimers Dis. *2011*, 280502.

Matsagas, K., Lim, D.B., Horwitz, M., Rizza, C.L., Mueller, L.D., Villeponteau, B., and Rose, M.R. (2009). Long-term functional side-effects of stimulants and sedatives in Drosophila melanogaster. PLoS ONE *4*, e6578.

Mattson, M.P. (2008). Hormesis defined. Ageing Res. Rev. *7*, 1–7.

McColl, G., Killilea, D.W., Hubbard, A.E., Vantipalli, M.C., Melov, S., and Lithgow, G.J. (2008). Pharmacogenetic analysis of lithium-induced delayed aging in Caenorhabditis elegans. J. Biol. Chem. *283*, 350–357.

McElwee, J.J., Schuster, E., Blanc, E., Piper, M.D., Thomas, J.H., Patel, D.S., Selman, C., Withers, D.J., Thornton, J.M., Partridge, L., and Gems, D. (2007).

OPEN
ACCESS
CellPress

Evolutionary conservation of regulated longevity assurance mechanisms. Genome Biol. *8*, R132.

Meijer, L., Flajolet, M., and Greengard, P. (2004). Pharmacological inhibitors of glycogen synthase kinase 3. Trends Pharmacol. Sci. *25*, 471–480.

Minois, N. (2000). Longevity and aging: beneficial effects of exposure to mild stress. Biogerontology *1*, 15–29.

Misra, J.R., Horner, M.A., Lam, G., and Thummel, C.S. (2011). Transcriptional regulation of xenobiotic detoxification in Drosophila. Genes Dev. *25*, 1796–1806.

Pang, S., Lynn, D.A., Lo, J.Y., Paek, J., and Curran, S.P. (2014). SKN-1 and Nrf2 couples proline catabolism with lipid metabolism during nutrient deprivation. Nat. Commun. *5*, 5048.

Phiel, C.J., and Klein, P.S. (2001). Molecular targets of lithium action. Annu. Rev. Pharmacol. Toxicol. *41*, 789–813.

Pitoniak, A., and Bohmann, D. (2015). Mechanisms and functions of Nrf2 signaling in Drosophila. Free Radic. Biol. Med. *88* (*Pt B*), 302–313.

Rada, P., Rojo, A.I., Evrard-Todeschi, N., Innamorato, N.G., Cotte, A., Jaworski, T., Tobón-Velasco, J.C., Devijver, H., García-Mayoral, M.F., Van Leuven, F., et al. (2012). Structural and functional characterization of Nrf2 degradation by the glycogen synthase kinase 3/β-TrCP axis. Mol. Cell. Biol. *32*, 3486–3499.

Rattan, S.I.S. (2008). Hormesis in aging. Ageing Res. Rev. *7*, 63–78.

Riera, C.E., and Dillin, A. (2015). Can aging be 'drugged'? Nat. Med. *21*, 1400–1405.

Rizak, J., Tan, H., Zhu, H., and Wang, J.-F. (2014). Chronic treatment with the mood-stabilizing drug lithium up-regulates nuclear factor E2-related factor 2 in rat pheochromocytoma PC12 cells in vitro. Neuroscience *256*, 223–229.

Rojo, A.I., Rada, P., Egea, J., Rosa, A.O., López, M.G., and Cuadrado, A. (2008). Functional interference between glycogen synthase kinase-3 beta and the transcription factor Nrf2 in protection against kainate-induced hippocampal cell death. Mol. Cell. Neurosci. *39*, 125–132.

Salazar, M., Rojo, A.I., Velasco, D., de Sagarra, R.M., and Cuadrado, A. (2006). Glycogen synthase kinase-3beta inhibits the xenobiotic and antioxidant cell response by direct phosphorylation and nuclear exclusion of the transcription factor Nrf2. J. Biol. Chem. *281*, 14841–14851.

Salih, D.A.M., and Brunet, A. (2008). FoxO transcription factors in the maintenance of cellular homeostasis during aging. Curr. Opin. Cell Biol. *20*, 126–136.

Sarkar, S., Floto, R.A., Berger, Z., Imarisio, S., Cordenier, A., Pasco, M., Cook, L.J., and Rubinsztein, D.C. (2005). Lithium induces autophagy by inhibiting inositol monophosphatase. J. Cell Biol. *170*, 1101–1111.

Schäfer, M., Goodenough, S., Moosmann, B., and Behl, C. (2004). Inhibition of glycogen synthase kinase 3 beta is involved in the resistance to oxidative stress in neuronal HT22 cells. Brain Res. *1005*, 84–89.

Skorupa, D.A., Dervisefendic, A., Zwiener, J., and Pletcher, S.D. (2008). Dietary composition specifies consumption, obesity, and lifespan in Drosophila melanogaster. Aging Cell *7*, 478–490.

Slack, C., Alic, N., Foley, A., Cabecinha, M., Hoddinott, M.P., and Partridge, L. (2015). The Ras-Erk-ETS-signaling pathway is a drug target for longevity. Cell *162*, 72–83.

Sofola-Adesakin, O., Castillo-Quan, J.I., Rallis, C., Tain, L.S., Bjedov, I., Rogers, I., Li, L., Martinez, P., Khericha, M., Cabecinha, M., et al. (2014). Lithium suppresses Aβ pathology by inhibiting translation in an adult Drosophila model of Alzheimer's disease. Front. Aging Neurosci. *6*, 190.

Steinbaugh, M.J., Sun, L.Y., Bartke, A., and Miller, R.A. (2012). Activation of genes involved in xenobiotic metabolism is a shared signature of mouse models with extended lifespan. Am. J. Physiol. Endocrinol. Metab. *303*, E488–E495.

Steinbaugh, M.J., Narasimhan, S.D., Robida-Stubbs, S., Moronetti Mazzeo, L.E., Dreyfuss, J.M., Hourihan, J.M., Raghavan, P., Operaña, T.N., Esmaillie, R., and Blackwell, T.K. (2015). Lipid-mediated regulation of SKN-1/Nrf in response to germ cell absence. eLife *4*.

Suganthi, M., Sangeetha, G., Gayathri, G., and Ravi Sankar, B. (2012). Biphasic dose-dependent effect of lithium chloride on survival of human hormone-dependent breast cancer cells (MCF-7). Biol. Trace Elem. Res. *150*, 477–486.

Sykiotis, G.P., and Bohmann, D. (2008). Keap1/Nrf2 signaling regulates oxidative stress tolerance and lifespan in Drosophila. Dev. Cell *14*, 76–85.

Sykiotis, G.P., and Bohmann, D. (2010). Stress-activated cap'n'collar transcription factors in aging and human disease. Sci. Signal. *3*, re3.

Sykiotis, G.P., Habeos, I.G., Samuelson, A.V., and Bohmann, D. (2011). The role of the antioxidant and longevity-promoting Nrf2 pathway in metabolic regulation. Curr. Opin. Clin. Nutr. Metab. Care *14*, 41–48.

Takahashi-Yanaga, F. (2013). Activator or inhibitor? GSK-3 as a new drug target. Biochem. Pharmacol. *86*, 191–199.

Tam, Z.Y., Gruber, J., Ng, L.F., Halliwell, B., and Gunawan, R. (2014). Effects of lithium on age-related decline in mitochondrial turnover and function in Caenorhabditis elegans. J. Gerontol. A Biol. Sci. Med. Sci. *69*, 810–820.

Taylor, R.C., and Dillin, A. (2013). XBP-1 is a cell-nonautonomous regulator of stress resistance and longevity. Cell *153*, 1435–1447.

Tullet, J.M.A. (2015). DAF-16 target identification in C. elegans: past, present and future. Biogerontology *16*, 221–234.

Tullet, J.M.A., Hertweck, M., An, J.H., Baker, J., Hwang, J.Y., Liu, S., Oliveira, R.P., Baumeister, R., and Blackwell, T.K. (2008). Direct inhibition of the longevity-promoting factor SKN-1 by insulin-like signaling in C. elegans. Cell *132*, 1025–1038.

Ulgherait, M., Rana, A., Rera, M., Graniel, J., and Walker, D.W. (2014). AMPK modulates tissue and organismal aging in a non-cell-autonomous manner. Cell Rep. *8*, 1767–1780.

Wang, L., Karpac, J., and Jasper, H. (2014). Promoting longevity by maintaining metabolic and proliferative homeostasis. J. Exp. Biol. *217*, 109–118.

Williams, R.S., and Harwood, A.J. (2000). Lithium therapy and signal transduction. Trends Pharmacol. Sci. *21*, 61–64.

Zarse, K., Terao, T., Tian, J., Iwata, N., Ishii, N., and Ristow, M. (2011). Low-dose lithium uptake promotes longevity in humans and metazoans. Eur. J. Nutr. *50*, 387–389.

Zhu, F., Li, Q., Zhang, F., Sun, X., Cai, G., Zhang, W., and Chen, X. (2015). Chronic lithium treatment diminishes the female advantage in lifespan in *Drosophila melanogaster*. Clin. Exp. Pharmacol. Physiol. *42*, 617–621.

Cell Reports, Volume *15*

Supplemental Information

Lithium Promotes Longevity through GSK3/NRF2-Dependent Hormesis

Jorge Iván Castillo-Quan, Li Li, Kerri J. Kinghorn, Dobril K. Ivanov, Luke S. Tain, Cathy Slack, Fiona Kerr, Tobias Nespital, Janet Thornton, John Hardy, Ivana Bjedov, and Linda Partridge

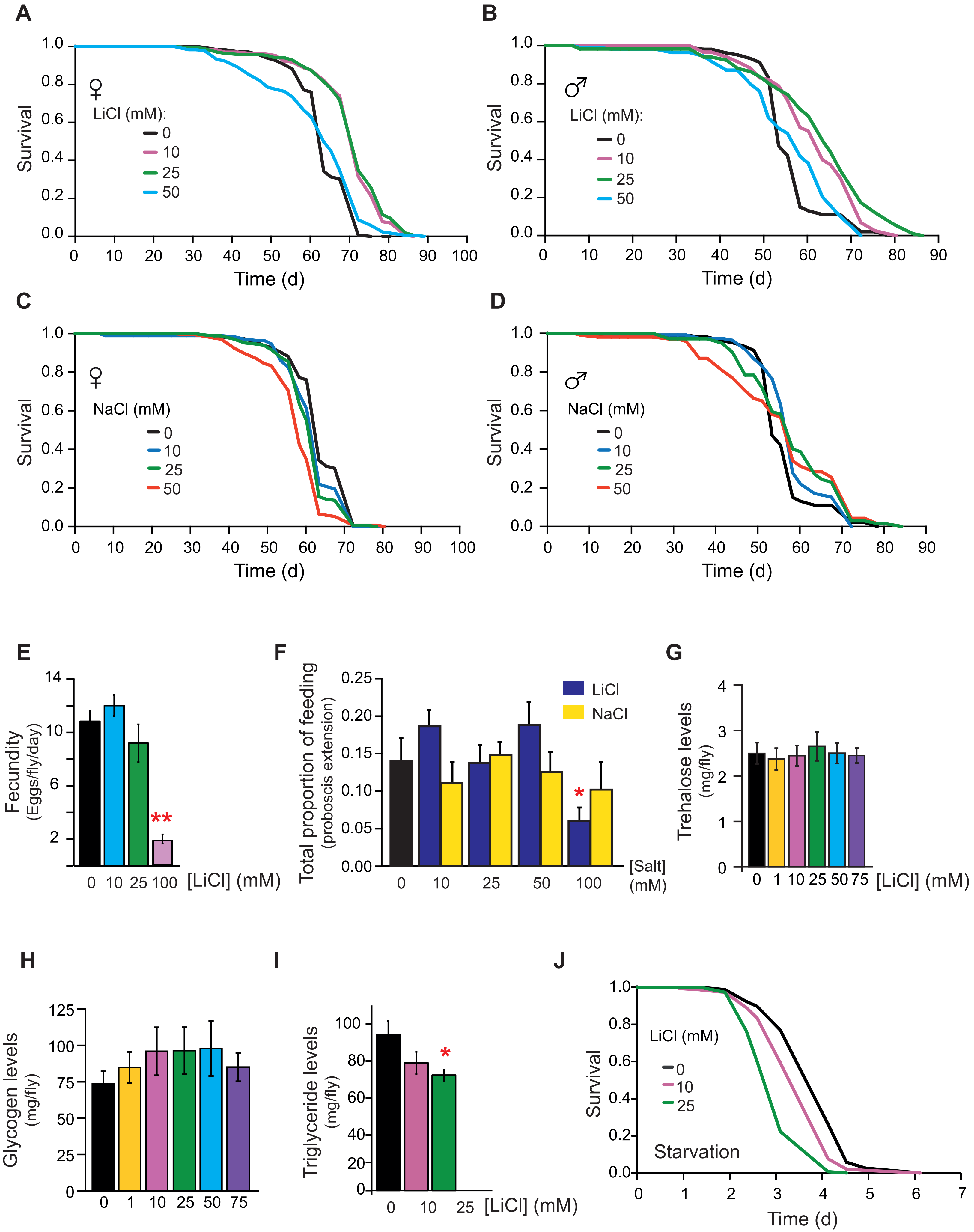

A
B
C
D
E
F
G
H
I
J
Survival
Time (d)
♀
♂
LiCl (mM):
0
10
25
50
NaCl (mM)
Fecundity
(Eggs/fly/day)
[LiCl] (mM)
Total proportion of feeding
(proboscis extension)
LiCl
NaCl
[Salt]
(mM)
Trehalose levels
(mg/fly)
Glycogen levels
(mg/fly)
Triglyceride levels
(mg/fly)
LiCl (mM)
Starvation

SUP FIGURE 1

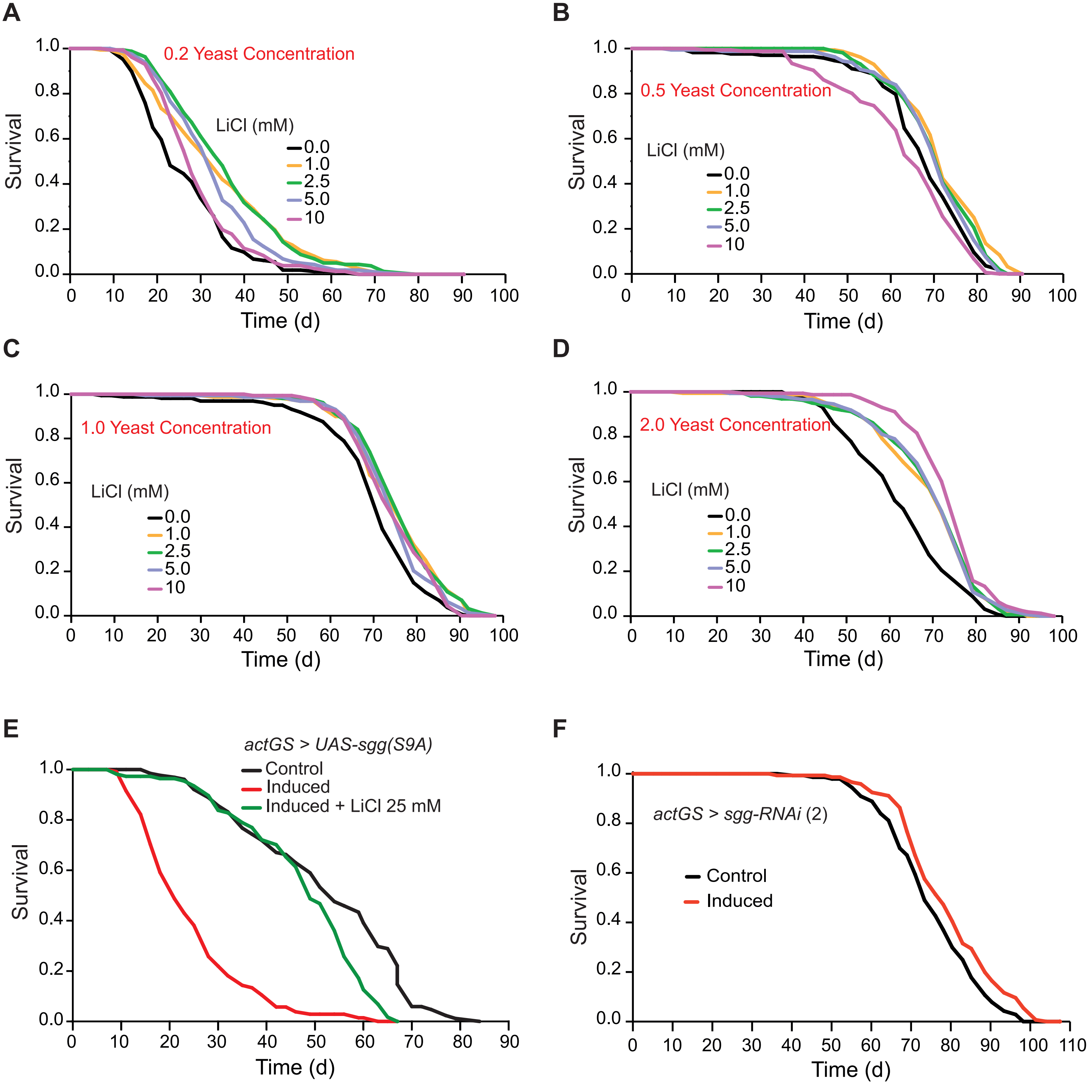
A
0.2 Yeast Concentration
Survival
Time (d)
LiCl (mM)
0.0
1.0
2.5
5.0
10
B
0.5 Yeast Concentration
Survival
Time (d)
LiCl (mM)
0.0
1.0
2.5
5.0
10
C
1.0 Yeast Concentration
Survival
Time (d)
LiCl (mM)
0.0
1.0
2.5
5.0
10
D
2.0 Yeast Concentration
Survival
Time (d)
LiCl (mM)
0.0
1.0
2.5
5.0
10
E
actGS > UAS-sgg(S9A)
Control
Induced
Induced + LiCl 25 mM
Survival
Time (d)
F
actGS > sgg-RNAi (2)
Control
Induced
Survival
Time (d)

SUP FIGURE 2

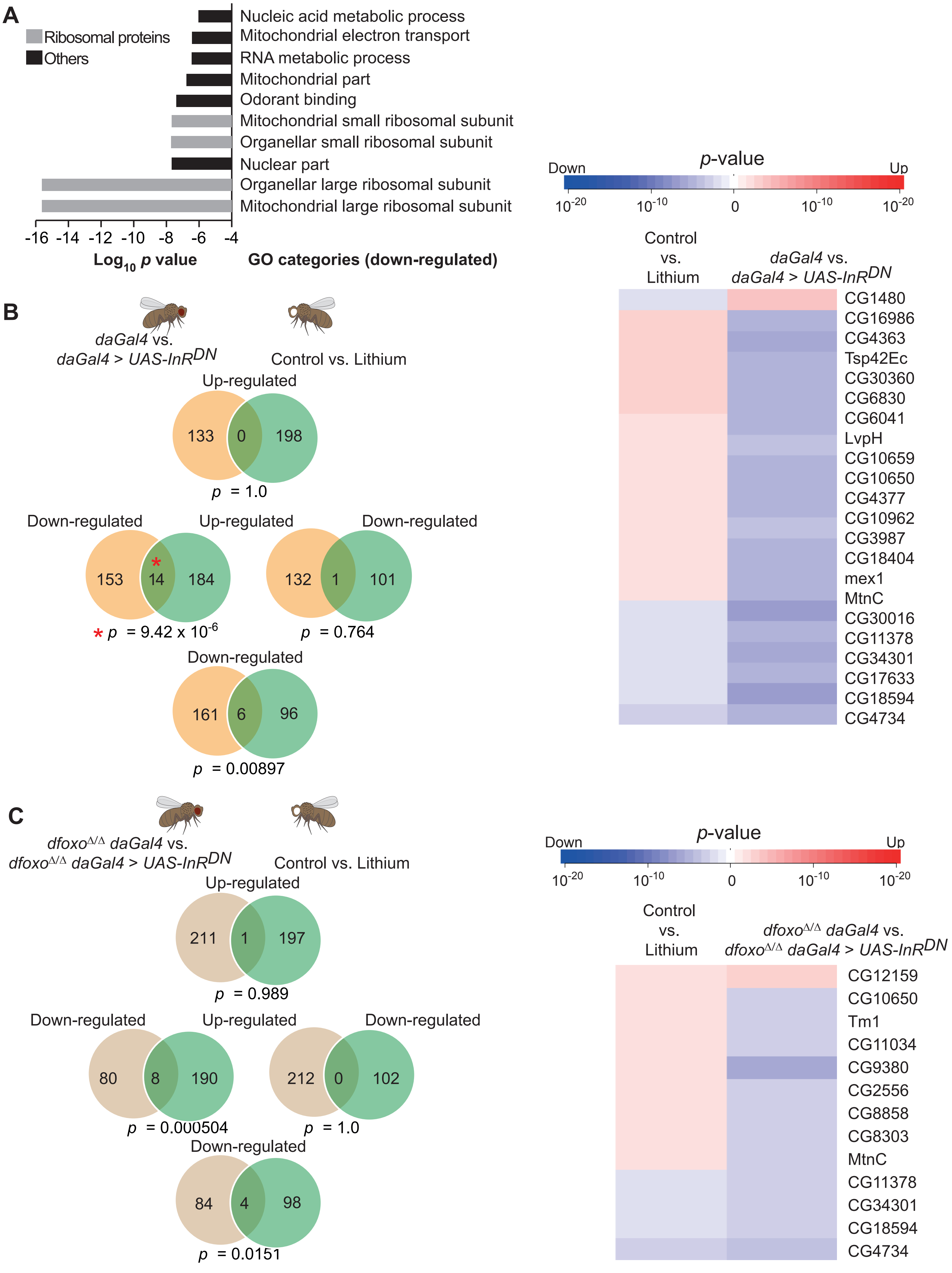
A
Ribosomal proteins
Others
Nucleic acid metabolic process
Mitochondrial electron transport
RNA metabolic process
Mitochondrial part
Odorant binding
Mitochondrial small ribosomal subunit
Organellar small ribosomal subunit
Nuclear part
Organellar large ribosomal subunit
Mitochondrial large ribosomal subunit
-16 -14 -12 -10 -8 -6 -4
Log10 p value
GO categories (down-regulated)
p-value
Down
Up
10-20 10-10 0 10-10 10-20
Control vs. Lithium
daGal4 vs. daGal4 > UAS-InRDN
CG1480
CG16986
CG4363
Tsp42Ec
CG30360
CG6830
CG6041
LvpH
CG10659
CG10650
CG4377
CG10962
CG3987
CG18404
mex1
MtnC
CG30016
CG11378
CG34301
CG17633
CG18594
CG4734
B
daGal4 vs. daGal4 > UAS-InRDN
Control vs. Lithium
Up-regulated
133 0 198
p = 1.0
Down-regulated
Up-regulated
Down-regulated
153 14 184
* p = 9.42 x 10-6
132 1 101
p = 0.764
Down-regulated
161 6 96
p = 0.00897
C
dfoxoΔ/Δ daGal4 vs. dfoxoΔ/Δ daGal4 > UAS-InRDN
Control vs. Lithium
Up-regulated
211 1 197
p = 0.989
Down-regulated
Up-regulated
Down-regulated
80 8 190
p = 0.000504
212 0 102
p = 1.0
Down-regulated
84 4 98
p = 0.0151
p-value
Down
Up
10-20 10-10 0 10-10 10-20
Control vs. Lithium
dfoxoΔ/Δ daGal4 vs. dfoxoΔ/Δ daGal4 > UAS-InRDN
CG12159
CG10650
Tm1
CG11034
CG9380
CG2556
CG8858
CG8303
MtnC
CG11378
CG34301
CG18594
CG4734

SUP FIGURE 3

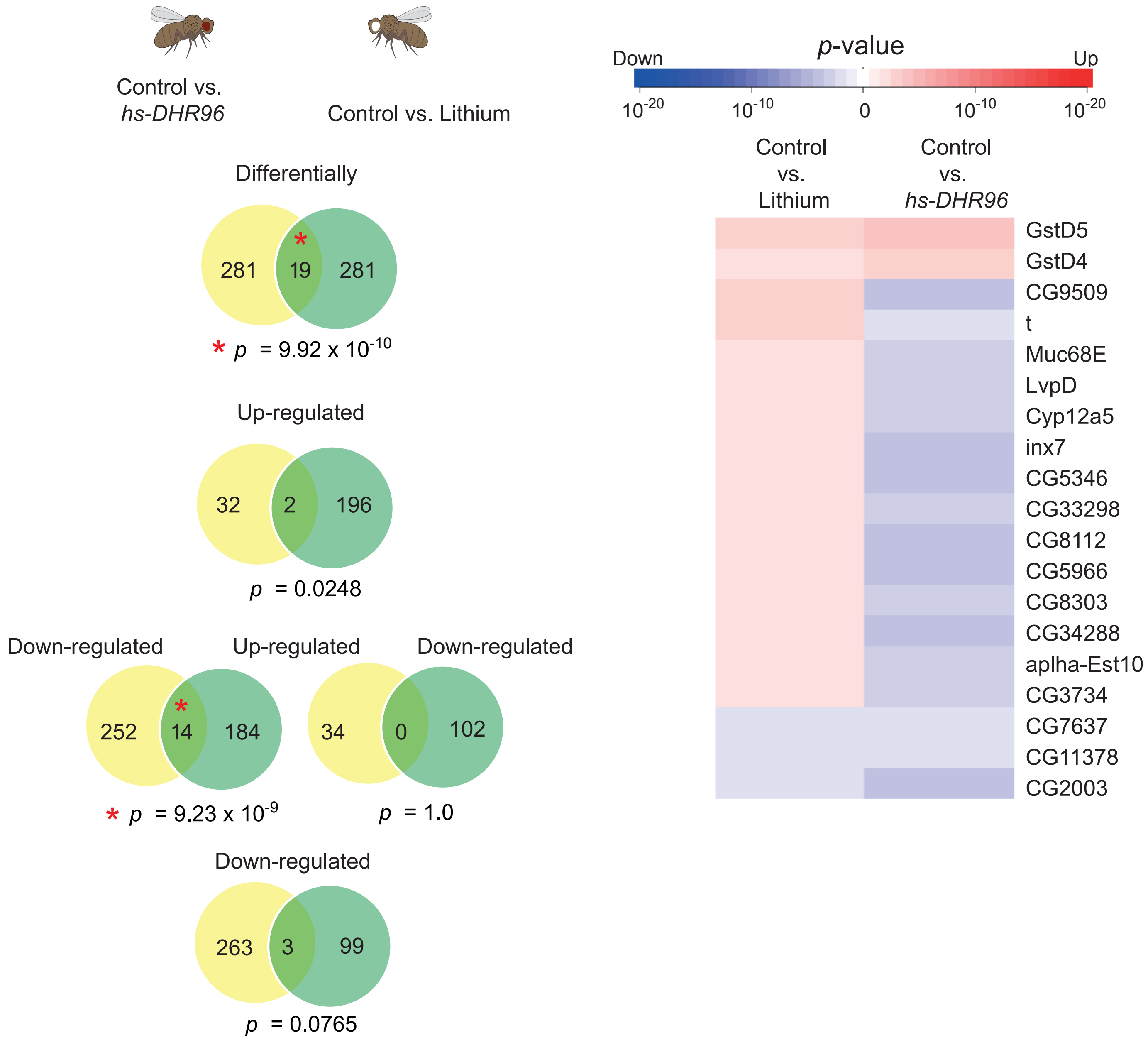
Control vs.
hs-DHR96
Control vs. Lithium
Differentially
281
19
281
* p = 9.92 x 10-10
Up-regulated
32
2
196
p = 0.0248
Down-regulated
Up-regulated
252
14
184
* p = 9.23 x 10-9
Up-regulated
Down-regulated
34
0
102
p = 1.0
Down-regulated
263
3
99
p = 0.0765
p-value
Down
Up
10-20
10-10
0
10-10
10-20
Control
vs.
Lithium
Control
vs.
hs-DHR96
GstD5
GstD4
CG9509
t
Muc68E
LvpD
Cyp12a5
inx7
CG5346
CG33298
CG8112
CG5966
CG8303
CG34288
aplha-Est10
CG3734
CG7637
CG11378
CG2003

SUP FIGURE 4

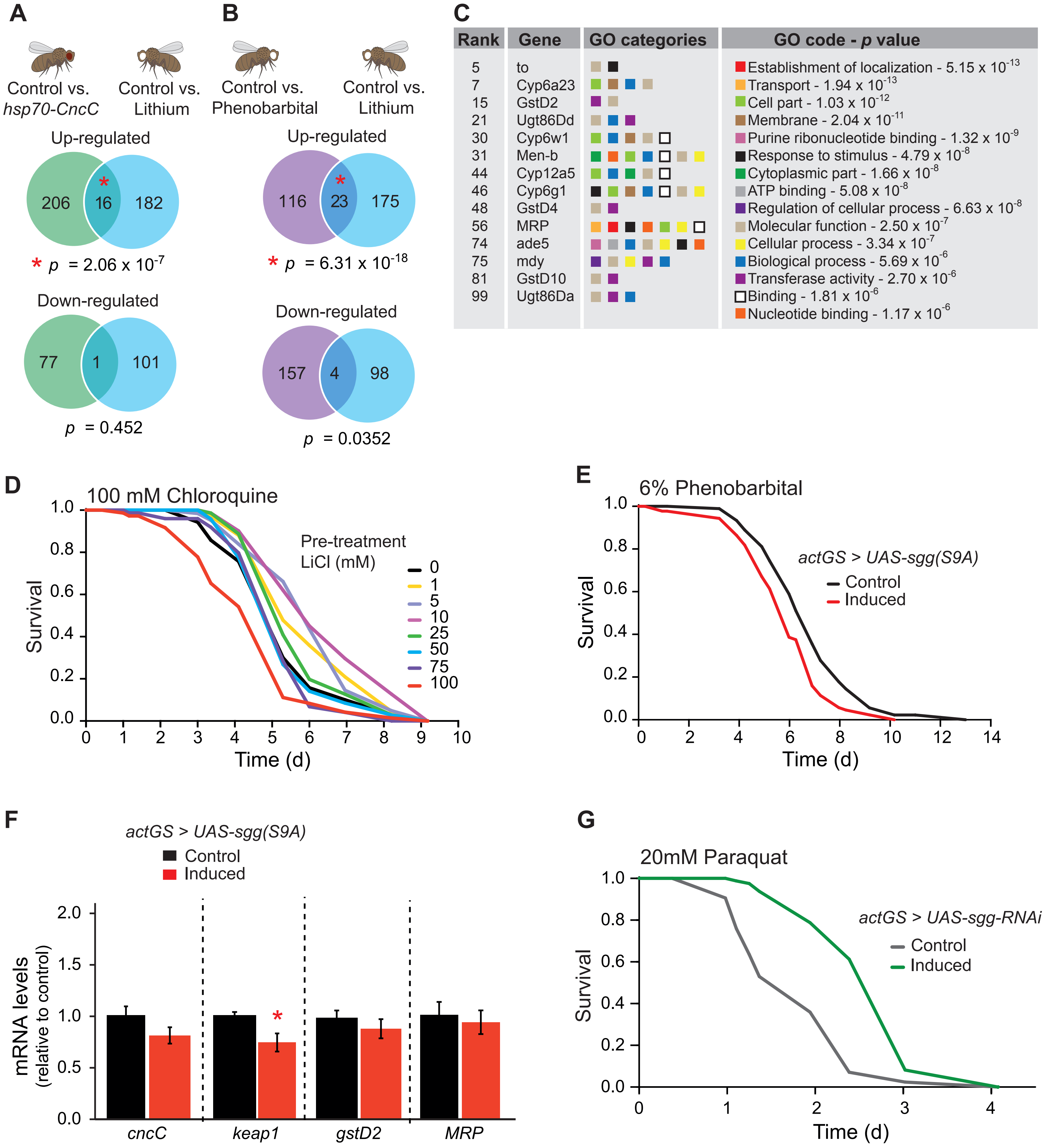
A
Control vs. hsp70-CncC
Control vs. Lithium
Up-regulated
206
16
182
* p = 2.06 x 10-7
Down-regulated
77
1
101
p = 0.452
B
Control vs. Phenobarbital
Control vs. Lithium
Up-regulated
116
23
175
* p = 6.31 x 10-18
Down-regulated
157
4
98
p = 0.0352
C
Rank
Gene
GO categories
GO code - p value
5 to
7 Cyp6a23
15 GstD2
21 Ugt86Dd
30 Cyp6w1
31 Men-b
44 Cyp12a5
46 Cyp6g1
48 GstD4
56 MRP
74 ade5
75 mdy
81 GstD10
99 Ugt86Da
Establishment of localization - 5.15 x 10-13
Transport - 1.94 x 10-13
Cell part - 1.03 x 10-12
Membrane - 2.04 x 10-11
Purine ribonucleotide binding - 1.32 x 10-9
Response to stimulus - 4.79 x 10-8
Cytoplasmic part - 1.66 x 10-8
ATP binding - 5.08 x 10-8
Regulation of cellular process - 6.63 x 10-8
Molecular function - 2.50 x 10-7
Cellular process - 3.34 x 10-7
Biological process - 5.69 x 10-6
Transferase activity - 2.70 x 10-6
Binding - 1.81 x 10-6
Nucleotide binding - 1.17 x 10-6
D
100 mM Chloroquine
Pre-treatment LiCl (mM)
0
1
5
10
25
50
75
100
Survival
Time (d)
E
6% Phenobarbital
actGS > UAS-sgg(S9A)
Control
Induced
Survival
Time (d)
F
actGS > UAS-sgg(S9A)
Control
Induced
mRNA levels (relative to control)
cncC
keap1
gstD2
MRP
G
20mM Paraquat
actGS > UAS-sgg-RNAi
Control
Induced
Survival
Time (d)

SUP FIGURE 5

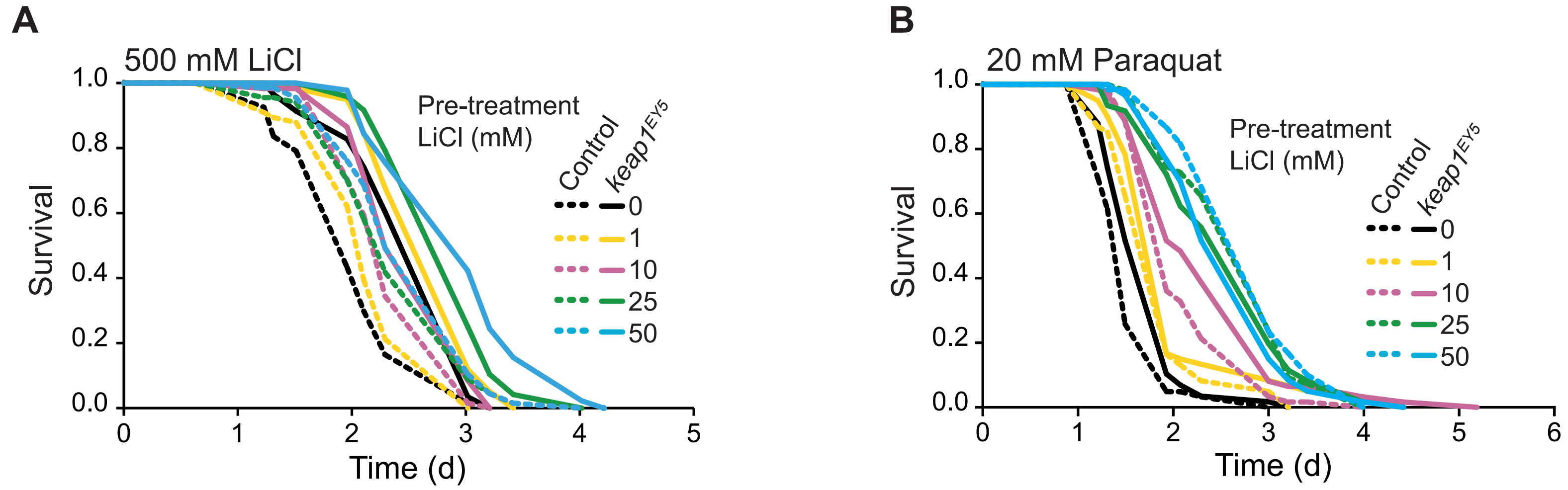

A
500 mM LiCl
Survival
Time (d)
Pre-treatment
LiCl (mM)
Control
keap1EY5
0
1
10
25
50
B
20 mM Paraquat
Survival
Time (d)
Pre-treatment
LiCl (mM)
Control
keap1EY5
0
1
10
25
50

SUP FIGURE 6

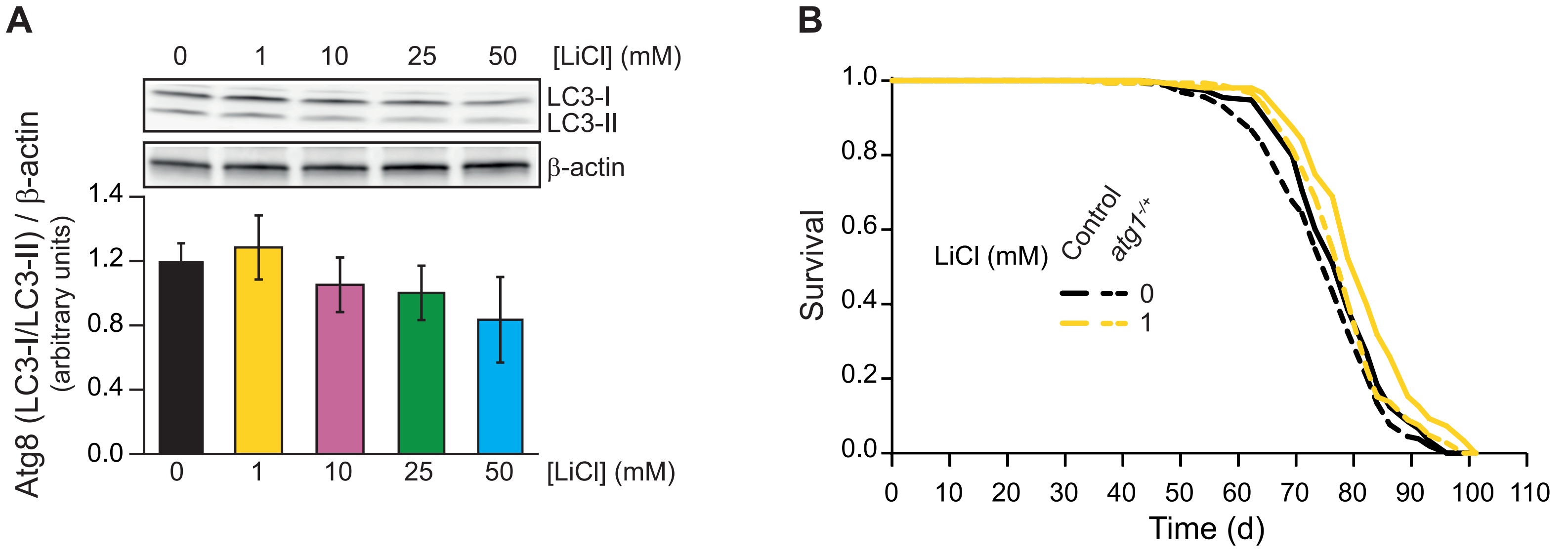

A
0
1
10
25
50
[LiCl] (mM)
LC3-I
LC3-II
β-actin
Atg8 (LC3-I/LC3-II) / β-actin
(arbitrary units)
1.4
1.2
0.8
0.4
0.0
0
1
10
25
50
[LiCl] (mM)
B
1.0
0.8
0.6
0.4
0.2
0.0
Survival
LiCl (mM)
Control
atg1-/+
0
1
0
10
20
30
40
50
60
70
80
90
100
110
Time (d)

SUP FIGURE 7

Supplemental Figure Legends

Figure S1. Lithium modulation of lifespan and metabolism.

(A) Lithium extended the lifespan of female *w*^{1118} flies (n = 150 flies per condition) at concentrations of 10 and 25 mM (+13% and +13-18% median and maximum lifespan extension; $p < 0.001$), but not at 50 mM ($p > 0.05$). Similar concentrations of NaCl did not extend lifespan (see Fig. S1C).

(B) Lithium extended lifespan of male *w*^{1118} flies (n = 150 flies per condition) at concentrations of 10 and 25 mM ($p < 0.001$, log-rank test). 10 mM lithium extended median lifespan by 18.5% and maximum lifespan by ~4%, while 25 mM lithium extended median lifespan by 23% and maximum by 13.5%. The higher dose of 50 mM lithium did not significantly extend lifespan ($p > 0.05$). Similar concentrations of NaCl did not extend lifespan (see Fig. S1D).

(C) Concentrations of NaCl similar to those of lithium that promote longevity (see Fig. 1A) did not extend lifespan of female *w*^{1118} flies (n = 150 flies per condition). 50 mM significantly shortened lifespan ($p < 0.05$) Note that this experiment was run in parallel to that in Fig. S1A, hence the lifespan data for controls (LiCl 0 mM) is the same.

(D) Concentrations of NaCl similar to those of lithium that promote longevity did not extend the lifespan of male *w*^{1118} flies (n = 150 flies per condition). Note that this experiment was run in parallel to that in Fig. S1B, hence the lifespan data for controls (LiCl 0 mM) is the same.

(E) Female *w*^{1118} flies treated with 10 or 25 mM lithium for 15 days did not show significant changes in fecundity, while flies treated with 100 mM lithium showed a significant ($p < 0.01$) reduction. N = 150 females per condition.

(F) Lithium or NaCl –treated flies for 15 days did not show changes in feeding behavior, except for 100 mM lithium which significantly reduced it (p < 0.05). N = 50 flies per condition.

(G) There were no significant differences in whole body trehalose levels after 15 days of lithium treatment of female w^{Dah} flies. Bars represent means ± SEM of triplicates of 6 biological repeats of 5 flies.

(H) There were no significant differences in whole body glycogen after 15 days of lithium treatment of female w^{Dah} flies. Bars represent means ± SEM of triplicates of 6 biological repeats of 5 flies.

(I) Triglycerides in female w^{1118} flies. Whole body triglyceride levels were reduced in flies after 15 days of lithium treatment compared with untreated controls. Bars represent medians of 6 replicas of 5 flies per condition ± SEM, * p < 0.05.

(J) Female w^{1118} flies pre-treated with increasing concentrations of lithium for 15 days showed a dose-dependent sensitivity to starvation (p < 0.01; n = 75 flies per condition).

Figure S2. Interactions of lithium with DR and GSK-3.

(A) Lithium increased lifespan under semi-starvation (0.2x yeast; n = 160 flies per condition). Doses from 1 to 5 mM lithium significantly extended lifespan (p < 0.001). 10 mM lithium treatment did not significantly change survival (p > 0.05).

(B) Doses of 1 and 2.5 mM lithium extended lifespan under semi-starvation (0.5x yeast; p < 0.05), while 10 mM lithium significantly shortened lifespan (p < 0.05). N = 160 flies per condition.

(C) All doses of lithium tested increased lifespan under the yeast concentration that maximized lifespan (1.0x yeast). $p < 0.001$ for 1 and 2.5 mM lithium versus control, and $p < 0.01$ for 5 and 10 mM; n = 160 flies per condition.

(D) All lithium concentrations tested extended lifespan under fully fed conditions (2.0x yeast; $p < 0.001$).

(E) Ubiquitous expression of a constitutively active *sgg* (*sgg(S9A)*) shortened lifespan ($p < 0.001$), and this shortening was almost completely reversed by 25 mM lithium ($p < 0.001$ for induced not treated and $p < 0.05$ for induced treated vs control). N = 150 flies per condition.

(F) A second RNAi line against *sgg* significantly extended lifespan ($p < 0.01$) when expressed ubiquitously using the actGS driver induced 2 days post-eclosion. N = 150 flies per condition.

Figure S3. Bioinformatics analyses of lithium-treated flies and interaction with IIS down-regulation.

(A) Top 10 of most significantly changed GO categories of down-regulated genes after lithium treatment.

(B) Venn diagrams and heatmap showing the overlap of genes differentially changed by lithium and the expression of an insulin receptor dominant negative (Alic et al., 2011). The only significant overlap over the Bonferroni corrected *p* value ($p < 0.0001$) was for genes down-regulated by IIS down-regulation and genes up-regulated by lithium ($p = 9.42^{-6}$).

(C) Venn diagrams and heatmap showing the overlap of genes differentially changed by lithium and expression of an insulin receptor dominant negative in a *dfoxo* null background (Alic et al., 2011). No significant overlap over the Bonferroni corrected p value ($p < 0.0001$) was detected in any direction.

Figure S4. Bioinformatic analyses of the interaction of lithium and DHR96.

Venn diagrams and heatmap showing the overlap of genes differentially changed by lithium and over-expression of *DHR96* (King-Jones et al., 2006). We detected a significant overlap over the Bonferroni corrected p value ($p < 0.0001$) for differentially expressed genes by both treatments. However, genes down-regulated by DHR96 significantly overlapped with those up-regulated by lithium treatment ($p = 9.23^{-10}$). No other significant overlap was detected in any direction.

Figure S5. Interactions of lithium transcriptional response with phenobarbital treatment and *cncC* over-expression.

(A) Genes up-regulated by *cncC* over-expression (Misra et al., 2011) overlapped with those up-regulated by lithium treatment ($p = 2.06 \times 10^{-7}$, Fisher's exact test). Down-regulated genes by c*ncC* over-expression (Misra et al., 2011) and lithium did not significantly overlap ($p = 0.452$, Fisher's exact test).

(B) Lithium and phenobarbital treatment (Misra et al., 2011) up-regulated an overlapping set of genes ($p = 6.31 \times 10^{-18}$, Fisher's exact test), but did not change a similar set of genes ($p = 0.0352$; statistical value was considered when $p < 0.0001$, Bonferroni correction for multiple comparisons) at the down-regulated level (Misra et al., 2011).

(C) GO category classification for up-regulated genes involved in detoxification commonly changed by lithium treatment and *cncC* over-expression.

(D) Pretreatment with 1 to 10 mM lithium significantly protected ($p < 0.05$) flies against the xenobiotic chloroquine. N = 75 flies per condition

(E) Over-expression of the S9A *sgg* mutant significantly reduced survival when stressed with phenobarbital ($p < 0.05$, log rank test). N= 75 flies per condition.

(F) Over-expression of *sgg(S9A)* significantly reduced *keap1* mRNA levels ($p < 0.05$), while non-significant trends were detected for *cncC*, *gstD2* and *MRP* mRNA levels ($p > 0.05$).

(G) RNAi-mediated down-regulation of *sgg* protected against paraquat ($p < 0.001$). N = 75 flies per condition.

Figure S6. Interaction of lithium with Keap1.

(A) Heterozygous loss of *keap1* ($keap1^{EY5}$) significantly protected against toxic concentrations of lithium ($p < 0.001$), and the combination of genetic and pharmacological activation of CncC further protected flies ($p < 0.001$ for 25 and 50 mM lithium treated mutants vs mutant or the same doses in the control flies). N = 75 flies per condition.

(B) The $keap1^{EY5}$ mutant significantly protected against paraquat-induced stress compared with control flies ($p < 0.01$), and potentiated the effect on lifespan of lithium pre-treatment ($p < 0.001$). N = 75 flies per condition.

Figure S7. Interaction of lithium with autophagy.

(A) 15 days of lithium treatment at several concentrations did not induce autophagy as measured by Atg8 (LC3) levels. There was a slight but non-significant reduction in the levels of Atg8**.**

(B) Lithium extended lifespan of flies with heterozygous loss of atg1. N= 120 flies per condition.

Supplemental Experimental Procedures

Fly Stocks and Husbandry

Stocks and experiments were kept at 25˚C and 65% humidity on a 12:12 light:dark cycle using standard sugar/yeast (SY) medium (15gL^{-1} agar, 50gL^{-1} sucrose, 100gL^{-1} autolyzed yeast, 100 gL^{-1} nipagin and 3 mL L^{-1} propionic acid). *daGS* was a gift from Veronique Monnier (Tricoire et al., 2009). The following stocks were obtained from Bloomington *Drosophila* Stock Center: *UAS-sgg*, *UAS-sgg(S9A)*. *UAS-sgg-RNAi w*as obtained from Vienna *Drosophila* Resource Center. *UAS-sgg-RNAi(2)* is part of the Transgenic RNAi project at Harvard Medical School and obtained from Bloomington. *actGS* (Poirier et al., 2008), *atg1*$^{-/+}$ (*atg*^{1}) (Lee et al., 2007) and all previously mentioned lines were backcrossed at least 6 times into the *w*^{Dah} background. *keap1*^{EY5}, *UAS-keap1*, *Gst-D-eGFP*, and *UAS-cncC-RNAi* were obtained from the Bohmann laboratory (Sykiotis and Bohmann, 2008). The *keap1* deletion (*keap1*^{Del}) was generated by P-element mediated male recombination using the P-element insertion line, Keap1[EY02632]. The resulting lines were screened by PCR for deletions that removed Keap1 coding sequence. Keap1-gDNA2 TTACTATTCCACCGTGTTGTG, Keap1-gDNA3 CCGCTCATTCCGTATGAAG. All *keap1*/*cncC* fly lines were backcrossed at least 6 times (except for the *yw; UAS-cncC-RNAi* that was used as a hybrid) into the *w*^{1118} background and then crossed into *w*^{Dah} females for experimental analyses. Gene induction (Osterwalder et al., 2001) was obtained by supplementing food with RU486 (Sigma) at 200 µM (stock prepared at 100 mM in 100% ethanol).

Lifespan Experiments

'Egg squirt' protocols were undertaken to ensure that all experimental flies were raised at similar larval densities (~300 eggs per bottle containing 70 mL of food). Flies were allowed to lay eggs for less than 24 hours on grape medium plates, with live yeast paste to encourage egg-laying. The eggs were collected from the plate by washing with phosphate buffered

saline (PBS) solution and collected into falcon tubes. The eggs were then allowed to settle to the bottom of the tube. Using a 100 µL Gilson pipette ~15-18 µL of egg suspension was dispensed into 200 mL glass bottles containing 70 mL SY medium. Following eclosion flies were transferred to fresh food for a 48 hr mating period. Under CO_2 anesthesia flies were divided by sex with 15-20 flies per vial. Flies were transferred to fresh vials of food three times a week throughout life. The number of dead flies found during each transfer was recorded. Lifespan curves were analyzed using a log-rank test. Maximum lifespans were calculated as the median of the last surviving 10% of the population.

Behavioral measurements

Feeding behavior was assessed by analyzing the proboscis extension of 10 vials of five flies each. The feeding behaviour assay relies on the fly proboscis extension (Wong et al., 2009). The number of flies engaged in proboscis extension was recorded and plotted as a function of observed versus estimated. To assess fecundity female flies were let lay eggs for a period of ~24 hours in vials containing standard medium. Vials containing eggs were frozen and kept at -20°C for a short time until eggs were counted (Grandison et al., 2009). Locomotor ability was assessed through the negative geotaxis paradigm which consists of a climbing assay in which 10-15 flies were placed in a 35 cm column (1.5 cm diameter) with a conic bottom end. Flies were tapped down and observed during 45 seconds. The column was separated into three areas: top, middle and bottom by two lines; one was 10 cm from top and the other was 3 cm from bottom). After 45 seconds flies located in each of these three sections were recorded into scoring sheets. Each column was evaluated three times to minimize trial error and a minimum of three vials per genotype/condition was evaluated in order to be able to perform statistical analyses. The recorded scoring showed the mean number of flies in total (ntot), at top (ntop), and at bottom (nbottom), which allowed obtaining a performance index. The performance index (PI) was calculated as ½ (ntot + ntop – nbottom / ntot). The performance index was plotted against time per each genotype and

condition. Results were analysed with two-way ANOVA and Tukey post-hoc (Rogers et al., 2012; Kerr et al., 2011).

Stress Assays

Phenobarbital, chloroquine and paraquat were obtained from Sigma. All were dissolved directly into the sugar/agar medium similarly prepared as the normal fly medium without yeast. No LiCl treatment was provided during the stress period. For starvation assays flies were maintained on a pure 1.5% agar medium.

Triglyceride and Storage Carbohydrate Measurements

To quantify triglyceride levels a colorimetric assay kit by Thermo Scientific was used (Bjedov et al., 2010; Kinghorn et al., 2015); 6 replicas of 5 female flies were homogenized in 0.05% Tween 20 and incubated for 5 minutes at 70ºC. Samples were then centrifuged for 5 minutes at 7000 rpm. The supernatant was transferred to fresh Eppendorfs and centrifuged for 10 minutes at maximum speed. For each sample, 175 µL was transferred to a fresh Eppendorf. 10 µL of each sample was dispensed into a well on a 96-well plate, with each sample in triplicate. 200 µL of Thermo Infinity Triglycerides solution was added to each well and the plate was left to incubate at 37ºC for 10 mins, after which time the absorbance in each well was measured at 574 nm. The lipid standards were treated in the same way as the samples all throughout and were prepared as 7 serial dilutions in 0.05% Tween 20. These were 2, 1, 0.5, 0.25, 0.125, 0.0625 and 0 µg/µL triglyceride. Samples were normalized to protein levels using the Thermo Scientific bicinchoninic acid protein (BCA) assay. Whole fly glycogen and trehalose were measured in once-mated females after 15 days of treatment as previously described (Broughton et al., 2005; Slack et al., 2011).

Western blot analysis

10 flies were homogenized in 2X Laemmli loading buffer (100 mM Tris 6.8, 20% glycerol, 4% SDS) containing 5% β-mercaptoethanol and then boiled for 5 min. Approximately 40 µg of protein extract was loaded per lane. Proteins were separated on SDS polyacrylamide gels and transferred to a nitrocellulose membrane. Membranes were blocked in 5% milk TBST for 1 hour at room temperature, after which they were probed for primary antibodies diluted in 5% BSA TBST overnight at 4°C. The following primary antibodies were used: GFP (Cell Signaling Technologies #2955, 1:1000), anti-phospho-GSK3 (Cell Signaling Technologies #9331, 1:500), anti-GSK-3 (US Biologicals #G8170, 1:1000), anti-Atg-8 (custom made, 1:1000), β-actin (Abcam #ab4801, 1:5000). Appropriate HRP-conjugated secondary antibodies (Abcam) were used (1:12,000). Blots were developed using the ECL detection system and analyzed using ImageJ program (US National Institutes of Health).

RT-qPCR

Total RNA was extracted from 15 flies using Trizol (GIBCO) according to the manufacturer's instructions. The concentration of total RNA purified for each sample was measured using an *Eppendorf biophotometer*. 2 µg of total RNA was then subjected to DNA digestion using DNAse I (Ambion), immediately followed by reverse transcription using the Superscript II system (Invitrogen) with oligo(dT) primers. Quantitative PCR was performed using the PRISM 7000 sequence-detection system (Applied Biosystems), SYBR Green (Molecular Probes), ROX Reference Dye (Invitrogen), and Hot Star Taq (Qiagen, Valencia, CA) by following manufacturer's instructions. Each sample was analysed in triplicate using a*ct5c* as reference gene. Primers used were: *gstD2*-F CATCGCCGTCTATCTGGTGGA, *gstD2*-R GGCATTGTCGTACCACCTGG, *keap1-F* CAAGGAGTCGGAGATGTCG, *keap1-R* GTAGAGGATGCGTGACATGG, *cncC-F* GAGGTGGAAATCGGAGATGA, *cncC-R* CTGCTTGTAGAGCACCTCAGC, *MRP*-F ACTTTACGCCCTGCTT, *MRP*-R TCACGTTCAGCTTGTTCCAC, a*ct5C*-F GCAGCAACTTCTTCGTCACA, a*ct5C*-R CATCAGCCAGCAGTCGTCTA.

Microarray analyses

Once-mated w^{1118} female flies were pre-treated for 10 days with 10 mM LiCl and snap frozen in liquid nitrogen, after which heads and thoraces were separated. RNA extraction was performed as per manufacturer's guidelines using Trizol. RNA was DNase treated and checked for quality by BioRad Experion. RNA was processed to cRNA, labeled and hybridized to a single-color Affymetrix GeneChip Drosophila Genome 2.0 Array, following manufacturer's protocol. These experiments were performed by the Max Planck-Genome-centre Cologne, Germany (http://mpgc.mpipz.mpg.de/home/).

Differential expression analysis

Raw data (cel files) were processed to correct for probe-sequence biases, by using bioconductor's package gcrma (http://www.bioconductor.org) in R (http://www.r-project.org). Affymetrix's MicroArray Suite 5.0 (bioconductor's package affy) was used to determine present target transcripts (Schuster et al., 2007). Transcripts were deemed present if the p-value was <0.111 and absent otherwise. The raw data were summarized and normalized by using Robust Multichip Average (rma function, part of bioconductor's package affy (Gautier et al., 2004)). In order to identify differentially expressed genes a linear model was fitted and differential expression was assessed using the empirical Bayes moderated *t*-statistic as implemented in R's limma package (Ritchie et al., 2015). P-values were adjusted for multiple hypotheses testing by applying the Benjamini and Hochberg correction for false discovery rate. Summarized probe-sets were mapped to transcripts using R's package "drosophila2.db". Transcripts not mapping to any known or predicted genes were excluded from further analysis.

The raw data for the wild-type vs. wild-type+Lithium(10mM) are deposited in ArrayExpress (http://www.ebi.ac.uk/arrayexpress) with identifier E-MTAB-3809. The following freely available microarray datasets were used: control vs. phenobarbital (E-GEOD-30087); control

vs. *hsp70*-CncC (E-GEOD-30087); *daGal4* vs. *daGal4 > UAS-InR*^{DN} and *dfoxo*$^{\Delta/\Delta}$ *daGal4* vs. *dfoxo*$^{\Delta/\Delta}$ *daGal4 > UAS-InR*^{DN} (E-TABM-757); control vs. *hs-DHR96* (E-GEOD-5097).

Statistical significance of differential expression gene sets

Statistical significance of overlaps of genes in two microarray experiments was determined using fisher's exact test. To account for multiple hypotheses testing a p-value cut-off of $\leq 1.0 \times 10^{-05}$ was used. After correcting for multiple hypothesis testing in the wild-type vs. wild-type+Lithium(10mM) (Benjamini and Hochberg correction for false discovery rate as described above), there were only two genes that were above the significance threshold of 0.05, thus we used the top 300 genes within each experiment to calculate significance of overlap. These genes could be viewed as top up or down-regulated genes among all of the genes part of an experiment.

Gene Ontology analysis

The Wilcoxon rank sum test, as implemented in Catmap (Breslin et al., 2004), was used to perform functional analysis, that is significant enrichment of Gene Ontology categories. FlyBase (http://flybase.org) gene identifiers were mapped to Gene Ontology identifiers (FlyBase version FB2014_01). Ranks of genes were based on the p-value derived from the Bayes *t*-statistic for differential expression. To account for multiple hypothesis testing, an enrichment of GO terms was deemed statistically significant if the p-value derived from the wilcoxon rank sum test was $\leq 1.0 \times 10^{-05}$. Gene lists were sorted by log-fold change and p-value. For all microarray experiments two sets of lists were derived; a gene list comprising most differentially up-regulated (log-fold change > 0) genes at the top of the list and most differentially down-regulated genes (log-fold change < 0) at the bottom of the list (termed up-to-down) and *vice verse* (termed down-to-up). If a GO category was found to be statistically significant in the up-to-down list, this GO was referred to as up-regulated, i.e. a large enough proportion of the genes that are part of this GO category were found to be up-regulated or at

the top of the list. If a GO category was found to be statistically significant in the down-to-up list, this GO was referred to as down-regulated, i.e. a large enough proportion of the genes that are part of this GO category were found to be down-regulated or at the top of the list.

Supplemental References

Alic, N., Andrews, T. D., Giannakou, M. E., Papatheodorou, I., Slack, C., Hoddinott, M. P., Cochemé, H. M., Schuster, E. F., Thornton, J. M., and Partridge, L. (2011). Genome-wide dFOXO targets and topology of the transcriptomic response to stress and insulin signalling. Mol. Syst. Biol. *7*, 502.

Bjedov, I., Toivonen, J. M., Kerr, F., Slack, C., Jacobson, J., Foley, A., and Partridge, L. (2010). Mechanisms of life span extension by rapamycin in the fruit fly Drosophila melanogaster. Cell Metab. *11*, 35–46.

Breslin, T., Edén, P., and Krogh, M. (2004). Comparing functional annotation analyses with Catmap. BMC Bioinformatics *5*, 193.

Broughton, S. J., Piper, M. D. W., Ikeya, T., Bass, T. M., Jacobson, J., Driege, Y., Martinez, P., Hafen, E., Withers, D. J., Leevers, S. J., et al. (2005). Longer lifespan, altered metabolism, and stress resistance in Drosophila from ablation of cells making insulin-like ligands. Proc. Natl. Acad. Sci. U. S. A. *102*, 3105–3110.

Gautier, L., Cope, L., Bolstad, B. M., and Irizarry, R. A. (2004). Affy - Analysis of Affymetrix GeneChip data at the probe level. Bioinformatics *20*, 307–315.

Grandison, R. C., Wong, R., Bass, T. M., Partridge, L., and Piper, M. D. W. (2009). Effect of a standardised dietary restriction protocol on multiple laboratory strains of Drosophila melanogaster. PLoS One *4*, e4067.

Kerr, F., Augustin, H., Piper, M. D. W., Gandy, C., Allen, M. J., Lovestone, S., and Partridge, L. (2011). Dietary restriction delays aging, but not neuronal dysfunction, in Drosophila models of Alzheimer’s disease. Neurobiol. Aging *32*, 1977–1989.

Kinghorn, K. J., Castillo-Quan, J. I., Bartolome, F., Angelova, P. R., Li, L., Pope, S., Cocheme, H. M., Khan, S., Asghari, S., Bhatia, K. P., et al. (2015). Loss of PLA2G6 leads to elevated mitochondrial lipid peroxidation and mitochondrial dysfunction. Brain *138*, 1801–1816.

King-Jones, K., Horner, M. A., Lam, G., and Thummel, C. S. (2006). The DHR96 nuclear receptor regulates xenobiotic responses in Drosophila. Cell Metab. *4*, 37–48.

Lee, S. B., Kim, S., Lee, J., Park, J., Lee, G., Kim, Y., Kim, J.-M., and Chung, J. (2007). ATG1, an autophagy regulator, inhibits cell growth by negatively regulating S6 kinase. EMBO Rep. *8*, 360–365.

Misra, J. R., Horner, M. A., Lam, G., and Thummel, C. S. (2011). Transcriptional regulation of xenobiotic detoxification in Drosophila. Genes Dev. *25*, 1796–1806.

Osterwalder, T., Yoon, K. S., White, B. H., and Keshishian, H. (2001). A conditional tissue-specific transgene expression system using inducible GAL4. Proc. Natl. Acad. Sci. U. S. A. *98*, 12596–12601.

Poirier, L., Shane, A., Zheng, J., and Seroude, L. (2008). Characterization of the Drosophila

gene-switch system in aging studies: a cautionary tale. Aging Cell *7*, 758–770.

Ritchie, M. E., Phipson, B., Wu, D., Hu, Y., Law, C. W., Shi, W., and Smyth, G. K. (2015). limma powers differential expression analyses for RNA-sequencing and microarray studies. Nucleic Acids Res. *43*, e47.

Rogers, I., Kerr, F., Martinez, P., Hardy, J., Lovestone, S., and Partridge, L. (2012). Ageing increases vulnerability to aβ42 toxicity in Drosophila. PLoS One *7*, e40569.

Schuster, E. F., Blanc, E., Partridge, L., and Thornton, J. M. (2007). Correcting for sequence biases in present/absent calls. Genome Biol. *8*, R125.

Slack, C., Giannakou, M. E., Foley, A., Goss, M., and Partridge, L. (2011). dFOXO-independent effects of reduced insulin-like signaling in Drosophila. Aging Cell *10*, 735–748.

Sykiotis, G. P., and Bohmann, D. (2008). Keap1/Nrf2 signaling regulates oxidative stress tolerance and lifespan in Drosophila. Dev. Cell *14*, 76–85.

Tricoire, H., Battisti, V., Trannoy, S., Lasbleiz, C., Pret, A. M., and Monnier, V. (2009). The steroid hormone receptor EcR finely modulates Drosophila lifespan during adulthood in a sex-specific manner. Mech. Ageing Dev. *130*, 547–552.

Wong, R., Piper, M. D. W., Wertheim, B., and Partridge, L. (2009). Quantification of food intake in Drosophila. PLoS One *4*, e6063.
